# Hepatic Gene Expression Profiling Reveals Key Pathways Involved in Leptin-Mediated Weight Loss in *ob/ob* Mice

**DOI:** 10.1371/journal.pone.0012147

**Published:** 2010-08-16

**Authors:** Ashok Sharma, Shoshana M. Bartell, Clifton A. Baile, Bo Chen, Robert H. Podolsky, Richard A. McIndoe, Jin-Xiong She

**Affiliations:** 1 Center for Biotechnology and Genomic Medicine, School of Medicine, Medical College of Georgia, Augusta, Georgia, United States of America; 2 Animal & Dairy Science, University of Georgia, Athens, Georgia, United States of America; 3 Department of Medicine, School of Medicine, Medical College of Georgia, Augusta, Georgia, United States of America; 4 Department of Pathology, School of Medicine, Medical College of Georgia, Augusta, Georgia, United States of America; Georgia Institute of Technology, United States of America

## Abstract

**Background:**

Leptin, a cytokine-like protein, plays an important role in the regulation of body weight through inhibition of food intake and stimulation of energy expenditure. Leptin circulates in blood and acts on the brain, which sends downstream signals to regulate body weight. Leptin therapy has been successful in treating leptin deficient obese patients. However, high levels of leptin have been observed in more common forms of obesity indicating a state of leptin resistance which limits the application of leptin in the treatment of obesity. If the central effect of leptin could be by-passed and genes which respond to leptin treatment could be regulated directly, new therapeutic targets for the treatment of obesity may be possible. The purpose of this study was to identify genes and subsequent pathways correlated with leptin-mediated weight loss.

**Methodology/Principal Findings:**

We utilized microarray technology to compare hepatic gene expression changes after two types of leptin administration: one involving a direct stimulatory effect when administered peripherally (subcutaneous: SQ) and another that is indirect, involving a hypothalamic relay that suppresses food intake when leptin is administered centrally (intracerebroventricular: ICV). We identified 214 genes that correlate with leptin mediated weight loss. Several biological processes such as mitochondrial metabolic pathways, lipid metabolic and catabolic processes, lipid biosynthetic processes, carboxylic acid metabolic processes, iron ion binding and glutathione S-transferases were downregulated after leptin administration. In contrast, genes involved in the immune system inflammatory response and lysosomal activity were found to be upregulated. Among the cellular compartments mitochondrion (32 genes), endoplasmic reticulum (22 genes) and vacuole (8 genes) were significantly over represented.

**Conclusions/Significance:**

In this study we have identified key molecular pathways and downstream genes which respond to leptin treatment and are involved in leptin-mediated weight loss. Many of these genes have previously been shown to be associated with obesity; however, we have also identified a number of other novel target genes. Further investigation will be required to assess the possible use of these genes and their associated protein products as therapeutic targets for the treatment of obesity.

## Introduction

Leptin, a cytokine-like protein containing 167 amino acids that is predominantly expressed in adipocytes, was discovered in 1994 by positional cloning of the obese (*ob*) gene [1]. Leptin plays a very important role in regulation of body weight and fat deposition through the inhibition of food intake and stimulation of energy expenditure [2]. Leptin-deficient (*ob/ob*) mice and leptin receptor-deficient (*db/db*) mice are severely obese, infertile and insulin resistant, have increased food intake, reduced body temperature and decreased energy expenditure, immune function and bone formation [3]–[5].

The liver has an integral role in lipid metabolism and also has significant energetic demands; therefore, studies have suggested that leptin's effect on metabolism may be mediated by the liver. Leptin-deficient mice have significant abnormalities in macronutrient metabolism, which can be corrected by leptin administration. A recent study has shown that acute leptin infusion rapidly reverses hepatic steatosis and plasma dyslipidemia induced by a high sucrose diet in rats [6], and the preservation of hepatic leptin action after a high sucrose diet is associated with the maintenance of low adiposity and plasma leptin concentrations.

Leptin therapy has been used to treat selected cases of both congenital and acquired leptin deficiencies in humans. Leptin usually works as a negative-feedback signal regulating the mass of the adipose tissue. However, high levels of leptin have been observed in obese humans and rodents, suggesting the development of leptin resistance in these cases [7]–[10]. An important component in leptin resistance appears to be the development of defective leptin uptake into the brain, downregulation of leptin receptors and decreased leptin sensitivity. The molecular mechanism by which leptin reduces body weight has been partially elucidated. There is a need to have a precise understanding of the metabolic pathways affected by leptin deficiency or leptin resistance. Therefore, understanding the mechanisms by which leptin and its targets influence energy balance could lead to new therapeutic targets for obesity.

The purpose of this study was to identify the genes which respond to leptin treatment and subsequent pathways correlated with leptin-mediated weight loss. We utilized microarray technology to compare two types of leptin administration: one involving a direct stimulatory effect when administered peripherally (subcutaneous: SQ) and another that is indirect, involving a hypothalamic relay that suppresses food intake when leptin is administered centrally (intracerebroventricular: ICV). We report here the impact of central and peripheral administration of leptin on food intake, body weight and body fat composition in *ob/ob* mice. We also report hepatic gene expression changes caused by central versus peripheral leptin administration.

## Materials and Methods

### Animals and design

Leptin deficient (*ob/ob*) female mice (11 wk old) on the C57BL6 background were obtained from Jackson Laboratory (Bar Harbor, ME). These mice were housed 2 per cage in shoebox cages with food and water available *ad libitum*. Their health, body weight and food intake were monitored and recorded weekly for 4 wk as they adapted to the pellet diet (LabDiet 5010, PMI Nutrition International). At 15 wk of age, the mice were divided into four treatment groups such that the mean body weight between treatments was not statistically different. Two groups of ten mice each were subjected to the subcutaneous (SQ) treatment (vehicle and leptin) and two groups of ten mice each were subjected to the intracerebroventricular (ICV) treatment (vehicle and leptin). Surprisingly, we observed negligible weight loss (ranging from −5.42% to 1.49%) in four animals of leptin-ICV treatment group, in which the cannula may not have been in place or treatment did not work. Therefore, these animals were removed from the analyses while evaluating effects of leptin administration on body weight, food intake and insulin levels. However, we thought that it would be interesting to determine if the gene expression profile of these animals were different from the animals showing weight loss. Therefore, for microarray analyses, we sub divided the LEP-ICV group into two groups: (a) LEP_ICV group: treatment worked (weight loss range: 25%–38%) and (b) LEP_ICV*: treatment did not work (weight loss −5.42% to 1.49%). This resulted in total five groups: VEH-SQ, LEP-SQ, VEH-ICV, LEP-ICV, and LEP-ICV*. To minimize the cost of the experiment, we randomly selected mice (n = 4) from these five groups for microarray profiling.

### Leptin Administration

Mice assigned to ICV treatments were surgically prepared with unilateral cannulas directed towards the right lateral cerebral ventricle as previously described [11]. Osmotic mini-pumps were surgically placed SQ to provide continuous infusion of control and test solutions for 12 days. For ICV treatments, polyethylene tubing filled with 6µl artificial cerebrospinal fluid (aCSF) was connected from the mini-pump to the ICV cannula. All mice were allowed to recover for 2 days after surgery; during this time 6µl of saline or 6µl of aCSF were infused prior to the treatment infusion. Leptin was continuously delivered subcutaneously (SQ; Control: 6 µl/d saline; Leptin group: 10.0 µg/d leptin) or into the lateral cerebral ventricle (ICV) (Control: 6 µl/d aCSF; Leptin group: 1.5 µg/d leptin) via osmotic pumps for 12 days. Body weight and food intakes were measured daily. Water was available *ad libitum*. The mice were sedated with CO_2_, sacrificed by guillotine prior to the collection of blood and tissues. Serum samples were stored at −80°C until assayed. Insulin, was determined using the Lumine×100™ instrumentation and a multiplex assay kit (Mouse Endocrine Immunoassay Panel catalog # MENDO-75K, protocol - http://www.lincoresearch.com/protocols/mendo-75k.html) manufactured by LINCO Research, Inc (St. Charles, MO). Brains of mice assigned to ICV treatments were sectioned to confirm proper cannula placement. All surgical and experimental procedures in this study were conducted in accordance with the NIH Guidelines and were approved by the Animal Care and Use Committee for The University of Georgia prior to initiating the studies under the Animal Use Proposal # A2007-10224-0.

### Microarray hybridization

Total RNA was isolated using TRI REAGENT (Sigma), according to the manufacturer's protocol. Purified RNA (200 ng) was further used for cRNA amplification and labeling with biotin using Target Amp cDNA synthesis kit (Epicenter catalog no. TAB1R6924). Approximately 750ng of labeled cRNA was hybridized to the MouseRef-8 v2.0 Expression BeadChips as per manufacturer's instructions. MouseRef-8 BeadChip targets 25,600 RefSeq transcripts and enables the interrogation of eight samples in parallel. After hybridization, arrays were washed, stained with Cy3-conjugated streptavidin, and scanned. Image data were analyzed with BeadStudio V2.0. Raw intensity data from BeadStudio were exported for further statistical analyses. The microarray data are MIAME compliant and have been deposited in NCBI Gene Expression Omnibus and are accessible through GEO Series accession number GSE20878.

### Differential gene expression analysis

Microarray data were normalized using the *lumi* package in R, using the variance stabilizing transformation (VST) of the package and robust spline normalization (RSN). Differential expression analyses were conducted using the LIMMA (Linear Models for Microarray Analysis) package from the Bioconductor project [12]. LIMMA uses an empirical Bayes approach that uses the variability in all genes for testing for signficiant differences, with this approach resulting in more stable inferences for a relatively small number of arrays [12], [13]. We used LIMMA to test for a linear association between gene expression levels and amount of weight loss (grams), using the amount of weight loss in a linear model and testing for the significance of the regression coefficient for weight loss. We used the false discovery rate (FDR) adjust for multiple testing [14]. B-statistics (the log of the odds of a gene showing either any association with weight loss) were calculated for each gene. Duplicate genes, when present, were removed and their expression levels averaged across the duplicates. We used a combination of “B statistic” and absolute value of slope (logFC) for selecting the differentially expressed genes. B statistic is the log odds of a gene showing either positive or negative correlation with weight loss (also known as a lod score) and logFC determines the magnitude of change in expression with weight loss. We used a cut-off of B>0 and logFC>0.02 (which corresponds to ∼2-fold or greater change in expression from lowest weight loss to highest weight loss point in graph). A total of 221 probes representing 214 unique genes were found significantly correlated with weight loss.

### Cluster analysis

Cluster analysis was performed for grouping differentially expressed genes exhibiting similar expression patterns using the HPCluster program [15]. HPCluster is a two-stage algorithm: the first stage is based on BIRCH (Balanced Iterative Reducing and Clustering using Hierarchies), while the second stage is a conventional k-Means. With BIRCH, a tree of cluster features defining the partitioning of high dimensional space is generated, followed by a conventional k-Means clustering of each cluster feature obtained with BIRCH. Two hundred and fourteen genes could be grouped into four clusters based on the expression data.

### Gene ontology analysis

Associations of the 214 differentially expressed genes with biological processes, molecular functions and cellular compartments were annotated using the GOTree Machine [16]. The GOTree Machine uses the hypergeometric test to evaluate the significance of enrichment for each category by determining if the observed number of gene counts exceeded the expected counts. The “FDR” method was used for adjusting the p-values for multiple testing. The results were visualized as a directed acyclic graph (tree) in order to show the relationship among the enriched GO categories.

## Results

The present study was designed to identify hepatic genes and pathways correlated with leptin mediated weight loss. Leptin deficient *ob/ob* mice were continuously administered with leptin via ICV (1.5 µg/d) or SQ (10 µg/d) over the 12-day treatment period. Liver RNA was extracted and cDNA microarray technology was used to explore the change in global gene expression profiles after two types of leptin administration. The four animals with negligible weight loss (ranging from −5.42% to 1.49%) after leptin-ICV treatment animals were removed from the analyses while evaluating effects of leptin administration on body weight, food intake and insulin levels. However we profiled these animals for gene expression, and the data were included for Identification of genes correlated with weight loss.

### Effects of central and peripheral leptin administration on body weight and food intake

**Figure 1:**
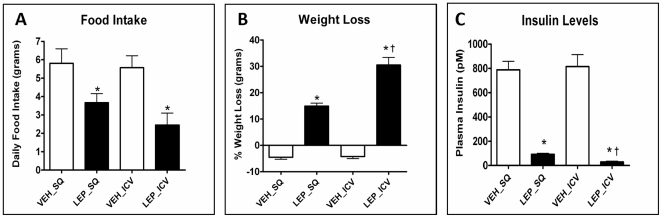
Effect of central (intracerebroventricular∶ICV) or peripheral (subcutaneous∶SQ) leptin treatment on food intake, weight loss, and serum insulin levels in ob/ob mice. Leptin deficient, *ob/ob* mice were continuously administered with leptin over 12-days using central or peripheral route of administration. Both types of leptin infusion significantly decrease food intake, weight, and serum insulin levels, as compared to their respective controls. The weight loss caused by central treatment is significantly greater than the peripheral treatment. Also, the decrease in the insulin level was more in the central group as compared to the peripheral group. VEH_SQ: vehicle subcutaneous treatment, LEP_SQ: leptin subcutaneous treatment, VEH_ICV: vehicle intracerebroventricular treatment, LEP_ICV: leptin intracerebroventricular treatment. Values are mean ± SEM; n = 9 Veh_SQ, n = 10 Lep_SQ, n = 9 Veh_ICV and n = 4 Lep_ICV; *P<0.05 leptin vs respective vehicle, † p<0.05 Lep_ICV vs Lep_SQ.

Both central and peripheral leptin administrations caused a significant reduction in food intake (FI) in leptin treated *ob/ob* mice as compared to vehicle treated *ob/ob* mice (SQ: 3.66±0.49 g (leptin) *vs* 5.80±0.79 g (vehile); ICV: 2.45±0.65 g (leptin) *vs* 5.57±0.65 g (vehile); Figure 1A). Similarly, there was a significant reduction in body weight (BW) in both ICV & SQ leptin groups as compared to their respective controls. Interestingly, percent weight loss in the ICV leptin-treated group was significantly greater than the weight loss in SQ leptin-treated group (SQ: 14.89±1.15 g (leptin) vs −4.50±0.76 (vehile); ICV: 30.51±2.87 g (leptin) vs −4.23±0.78 g (vehile); Figure 1B). Values are mean ± SEM.

### Leptin administration decreases serum insulin levels

The 12 days of leptin infusion in *ob/ob* mice, resulted in a significant decrease in serum insulin levels in both central and peripheral leptin treatment groups, as compared to their respective controls (SQ: 91.79±8.91 (leptin) vs 787.7±70.74 pM (vehile); ICV: 29.25±6.44 (leptin) vs 815.2±98.15 pM (vehile), Figure 1C). There was also a significant difference between the leptin-treated groups; the decrease in the insulin levels being greater in the ICV group as compared to the SQ group (Figure 1C). Values are mean ± SEM.

### Identification of genes correlated with leptin-mediated weight loss

**Figure 2:**
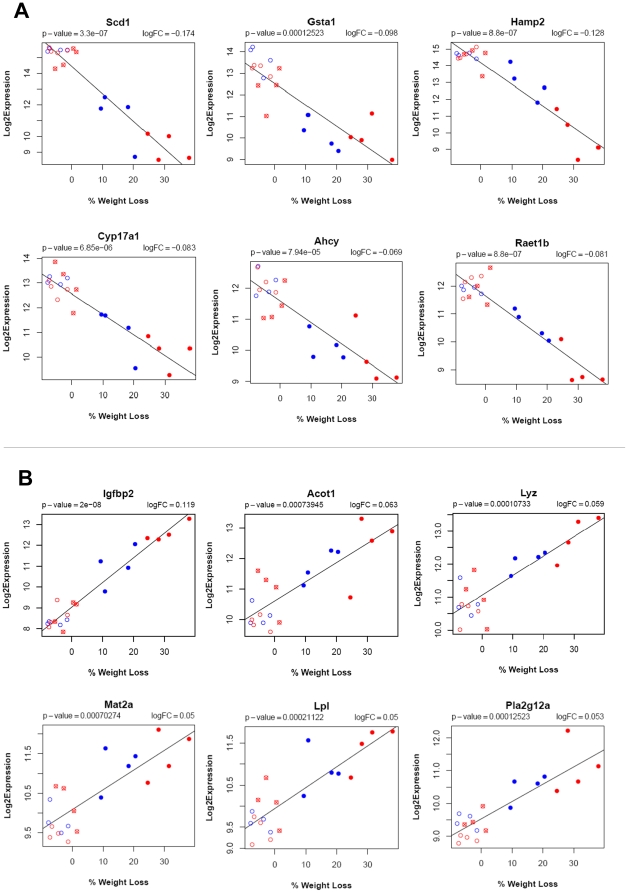
Regression plots of top six downregulated and top six upregulated genes. The regression analysis was performed using “*LIMMA*” to identify genes whose expression is significantly correlated with leptin mediated weight loss. Blue open circle is vehicle subcutaneous treatment; Blue filled circle is leptin subcutaneous treatment; Red open circle is vehicle intracerebroventricular treatment; Red filled circle is leptin intracerebroventricular treatment; Red crossed circle is leptin intracerebroventricular treatment but no weight loss.

We adopted microarray technology to compare the global gene expression profiles after the central (ICV) and peripheral (SQ) leptin treatments in *ob/ob* mice. C57BL6 mice were used for the baseline gene expression. Two hundred and twenty-one probes representing 214 unique genes were found to be significantly correlated with leptin mediated weight loss. We have listed the top 15 upregulated genes (with a two-fold or greater change in expression for each treatment) in Table 1 and 37 downregulated genes in Table 2. The plots of the regression analyses (expression vs weigh loss graphs) of the top six upregulated and top six downregulated genes mentioned above are shown in Figure 2. The phenotype data and weight loss of four ICV leptin treated animals (represented by red crossed circle) were very similar to the vehicle treated animals. Sectioning of the brain could not confirm placement of the cannula in two of these animals. As seen in the heatmap and correlation plots, the expression of these animals is very similar to the vehicle treated animals. Correlation plots of all 214 differentially expressed genes are available in supplementary material (File S1: Top-214 Correlation plots.pdf).

**Table 1:** Top 15 upregulated genes involved in leptin mediated weight loss.

Symbol	FC**_**SQ^a^	FC**_**ICV^b^	FC**_**ob/B6^c^	adj.P.Val^d^	Definition
**Igfbp2**	6.54	16.10	0.06	2.10E-08	insulin-like growth factor binding protein 2
**Acot1**	3.12	5.60	0.65	0.000739	acyl-CoA thioesterase 1
**Lyz**	2.33	4.93	0.41	0.000107	Lysozyme
**Mat2a**	2.55	4.10	0.37	0.000703	methionine adenosyltransferase II, alpha
**Lpl**	2.32	4.03	0.86	0.000211	lipoprotein lipase (Lpl)
**Pla2g12a**	2.04	4.57	0.66	0.000125	phospholipase A2, group XIIA
**Wbp5**	3.01	3.44	0.35	0.000535	WW domain binding protein 5
**C1qb**	2.32	3.22	0.28	0.000584	complement component 1, q subcomponent, beta polypeptide
**H2-Ab1**	2.52	2.88	0.50	0.001534	histocompatibility 2, class II antigen A, beta 1
**Hadhb**	2.04	3.17	0.99	0.000344	hydroxyacyl-Coenzyme A dehydrogenase
**Egfr**	2.58	2.48	0.16	0.001144	epidermal growth factor receptor
**Slc25a33**	2.60	2.43	0.38	0.000271	solute carrier family 25, member 33
**Slc40a1**	2.24	2.77	0.68	0.001166	solute carrier family 40 (iron-regulated transporter), member 1
**LOC100046120**	2.30	2.65	0.33	0.001791	s musculus similar to clusterin
**Rps11**	2.12	2.52	0.38	0.001243	ribosomal protein S11

a FC_SQ: fold change in gene expression after peripheral (subcutaneous) leptin administration as compared to vehicle subcutaneous treatment.

b FC_ICV: fold change in gene expression after central (intracerebroventricular) leptin administration as compared to vehicle intracerebroventricular treatment.

c FC_ob/B6: fold change in gene expression in vehicle treated *ob/ob* mice as compared to the expression in B6 mice.

d adj.P.Val: adj. p-value of regression analysis performed to test for a linear association between gene expression levels and amount of weight loss.

**Table 2:** Top 37 downregulated genes involved in leptin mediated weight loss.

Symbol	FC**_**SQ^a^	FC**_**ICV^b^	FC**_**ob/B6^c^	adj.P.Val^d^	Definition
**Scd1**	0.05	0.01	4.60	3.33E-07	stearoyl-Coenzyme A desaturase 1
**Gsta1**	0.09	0.11	41.28	0.000125	glutathione S-transferase, alpha 1
**Hamp2**	0.32	0.03	2.16	8.76E-07	hepcidin antimicrobial peptide 2
**Cyp17a1**	0.24	0.17	2.59	6.85E-06	cytochrome P450, family 17, subfamily a, polypeptide 1
**Ahcy**	0.25	0.18	4.50	7.94E-05	S-adenosylhomocysteine hydrolase
**Raet1b**	0.41	0.12	7.26	8.76E-07	retinoic acid early transcript beta
**Sardh**	0.27	0.17	3.86	0.001766	sarcosine dehydrogenase
**Gsta2**	0.23	0.22	8.24	0.000131	glutathione S-transferase, alpha 2
**Rdh16**	0.29	0.20	5.93	5.65E-06	retinol dehydrogenase 16
**Aass**	0.29	0.22	4.63	0.000942	aminoadipate-semialdehyde synthase
**Pla2g4f**	0.23	0.28	4.14	0.000178	phospholipase A2, group IVF
**Lrtm1**	0.32	0.20	2.05	6.44E-05	leucine-rich repeats and transmembrane domains 1
**Gstt3**	0.41	0.12	2.67	2.13E-07	glutathione S-transferase, theta 3
**Thrsp**	0.35	0.21	0.90	0.000931	thyroid hormone responsive SPOT14 homolog
**Bhmt**	0.35	0.23	5.59	0.000696	betaine-homocysteine methyltransferase
**Selenbp2**	0.32	0.30	1.76	0.000285	selenium binding protein 2
**Rcan2**	0.30	0.33	3.01	8.09E-05	regulator of calcineurin 2
**Mmd2**	0.29	0.34	2.79	0.000309	monocyte to macrophage differentiation-associated 2
**Gamt**	0.36	0.29	1.85	0.000344	guanidinoacetate methyltransferase
**Dmgdh**	0.31	0.34	3.82	0.000703	dimethylglycine dehydrogenase precursor
**Olig1**	0.41	0.25	6.69	2.86E-06	oligodendrocyte transcription factor 1
**Tmie**	0.48	0.18	4.48	0.000109	transmembrane inner ear
**Snhg11**	0.35	0.31	3.98	0.000314	small nucleolar RNA host gene 11
**Kcnk5**	0.45	0.22	1.99	0.000682	potassium channel, subfamily K, member 5
**Aes**	0.36	0.31	2.66	0.000315	amino-terminal enhancer of split
**Nnmt**	0.48	0.21	1.29	0.002523	nicotinamide N-methyltransferase
**9130409I23Rik**	0.35	0.35	3.35	0.001156	RIKEN cDNA 9130409I23 gene
**Slc38a4**	0.40	0.32	5.60	0.002091	solute carrier family 38, member 4
**Slc25a1**	0.44	0.30	1.81	2.96E-05	solute carrier family 25 (mitochondrial carrier, citrate transporter), member 1
**Uroc1**	0.48	0.28	2.84	6.63E-05	urocanase domain containing 1
**Hsd17b10**	0.42	0.36	3.47	0.000125	hydroxysteroid (17-beta) dehydrogenase 10
**Pls3**	0.46	0.33	4.45	0.002206	plastin 3
**Fam158a**	0.44	0.39	2.84	0.00198	family with sequence similarity 158, member A
**C530044N13Rik**	0.47	0.39	3.14	0.002232	RIKEN cDNA C530044N13 gene
**Slc44a1**	0.48	0.40	2.09	5.28E-05	solute carrier family 44, member 1
**Oprs1**	0.47	0.43	2.03	0.001192	opioid receptor, sigma 1
**Mfsd2**	0.45	0.48	2.59	0.001226	major facilitator superfamily domain containing 2

a FC_SQ: fold change in gene expression after peripheral (subcutaneous) leptin administration as compared to vehicle subcutaneous treatment.

b FC_ICV: fold change in gene expression after central (intracerebroventricular) leptin administration as compared to vehicle intracerebroventricular treatment.

c FC_ob/B6: fold change in gene expression in vehicle treated *ob/ob* mice as compared to the expression in B6 mice.

d adj.P.Val: adj. p-value of regression analysis performed to test for a linear association between gene expression levels and amount of weight loss.

### Cluster analysis of correlated genes

**Figure 3:**
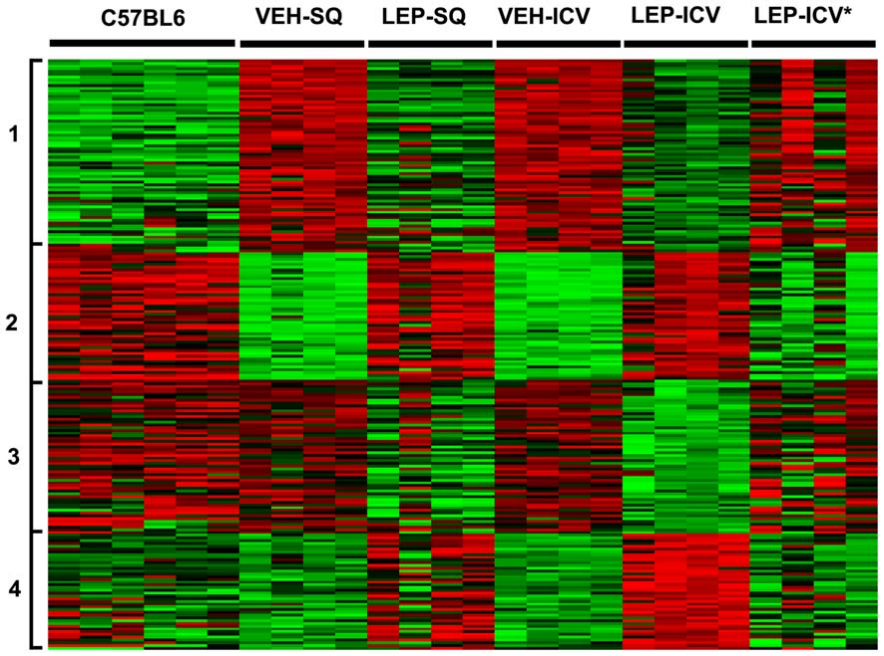
Heatmap of 214 differentially expressed genes showing gene expression levels across different samples. Each row represents one gene and each column represents one sample. Red indicates higher expression and green indicates lower expression. The global gene expression profiles were compared after the central and peripheral leptin treatments in *ob/ob* mice, while C57BL6 mice were used for the baseline gene expression. Cluster analysis on the gene expression data was performed to group the genes with similar expression patterns across experimental conditions. VEH-SQ: vehicle subcutaneous treatment, LEP-SQ: leptin subcutaneous treatment, VEH-ICV: vehicle intracerebroventricular treatment, LEP-ICV: leptin intracerebroventricular treatment, LEP-ICV*: represents four animals in which the cannula may not have been in place or treatment failed. While analyzing the phenotype data, we found that there was no weight loss in these four animals. Sectioning of the brain could not confirm placement of the cannula in these animals. The expression of these animals is very similar to the vehicle treated animals.

Next, we performed cluster analyses on the 214 genes significantly correlated with leptin mediated weight loss and found 4 clusters, each of which defined a unique pattern of expression (Figure 3). The four animals in which the cannula may not have been in place or treatment did not work are represented on the heatmap as LEP-ICV*. The expression pattern of these animals is similar to the vehicle groups. Seventy genes represented by cluster-1 are overexpressed in *ob/ob* mice as compared to B6 mice. After leptin replacement, the expression of these genes is normalized towards B6 mice. Similarly, 46 genes in cluster-2 are underexpressed in *ob/ob* as compared to B6 and leptin treatment could correct the expression back to normal. 56 genes of cluster-3 and 42 genes of cluster-4 have similar expression in B6 and *ob/ob* but these genes are downregulated (Cluster-3) or upregulated (Cluster-4) after leptin treatment. The complete spreadsheet with gene symbols and cluster assignment is available as supplementary material (File S2: 214_Gene_matrix.xls).

### Biological processes of genes regulated by leptin treatment

**Figure 4:**
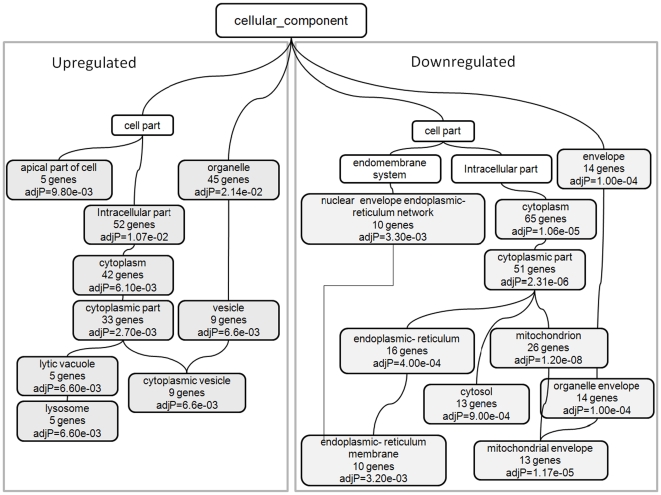
Gene Ontology tree representing the cellular compartments enriched by upregulated and downregulated set of genes. Gene ontology analysis was performed using the *GOTree Machine* software which performs the hypergeometric test to evaluate the significance of enrichment for each category by determining if the observed number of gene counts exceeded the expected counts. Among the cellular compartments mitochondrion and endoplasmic reticulum are enriched by downregulated genes and vacuole, vesicle, lysosome are significantly enriched by upregulated genes.

Using the GOTree Machine web application [16], the 214 differentially expressed genes were categorized as being associated with specific biological processes, molecular functions and cellular compartments. Statistically significant over represented biological processes were determined using the hypergeometric test. Among the cellular compartments, mitochondrion (32 genes), endoplasmic reticulum (22 genes) and vacuole (8 genes) were significantly over represented. To determine if there was a difference in enrichment of upregulated versus downregulated genes, the two groups (126 downregulated and 88 upregulated genes) were also mapped separately to GO categories. The results were visualized as a gene ontology tree in order to show the relationship among the enriched GO categories (Figure 4). Several biological processes such as cellular metabolic processes (101 genes), catabolic processes (36 genes), biosynthetic processes (28 genes), oxidation reduction (27 genes), response to chemical stimulus (35 genes), and inflammatory response (10 genes) were found to be significantly over represented. Among the molecular functions, oxidoreductase activity (26 genes), cofactor binding (14 genes), catalytic activity (89 genes), electron carrier activity (10 genes) and iron ion binding (12 genes) were significantly over represented. The complete gene ontology analyses results are available as supplementary material (File S3: Gene Ontology.pdf).

### Downregulated biological pathways after leptin administration in *ob/ob* mice

#### Mitochondrial metabolic pathways

The gene ontology analyses revealed that most significantly enriched cellular compartments among downregulated genes are mitochondrion and endoplasmic reticulum. Thirty-two genes could be mapped to mitochondrion, 26 genes were downregulated and 6 genes were upregulated after leptin treatment (Table 3). The most affected mitochondrial pathways are, ketone metabolic process, organic acid metabolic process, oxidation reduction, amino acid metabolic process, and nitrogen compound metabolic process. Among the molecular functions, electron carrier activity, catalytic activity, and coenzyme binding were most affected after leptin treatment. We found that overexpression of genes related to cellular metabolism is a characteristic of *ob/ob* mice, while leptin replacement normalizes this expression back towards B6. The livers of *ob/ob* mice are overloaded with large amounts of free fatty acids either through increased fatty acid biosynthesis or through decreased fatty acid oxidation. Leptin administration promotes a hypometabolic state and downregulates cellular metabolic activity. Surprisingly, this condition is very similar to hibernation [17]. The key to the survival under hibernation lays in an inherent ability to downregulate cellular metabolic rate to new hypometabolic steady states. There was a downregulation of most genes involved in amino acid metabolism, lipid, fatty acid and steroid metabolism and oxidation/reduction after leptin treatment in *ob/ob* mice. Mitochondrial metabolic pathways are major regulators of leptin-mediated weight loss.

**Table 3:** 32 mitochondrial genes involved in leptin signaling.

Symbol	FC**_**SQ^a^	FC**_**ICV^b^	FC**_**ob/B6^c^	adj.P.Val^d^	Definition
**Sardh**	0.27	0.17	3.86	0.001766	sarcosine dehydrogenase
**Aass**	0.29	0.22	4.63	0.000942	aminoadipate-semialdehyde synthase
**Gck**	0.58	0.21	2.96	0.000114	Glucokinase
**Abat**	0.53	0.25	2.30	7.01E-05	4-aminobutyrate aminotransferase
**Dmgdh**	0.31	0.34	3.82	0.000703	dimethylglycine dehydrogenase precursor
**Slc25a1**	0.44	0.30	1.81	2.96E-05	solute carrier family 25 (mitochondrial carrier, citrate transporter), member 1
**Hsd17b10**	0.42	0.36	3.47	0.000125	hydroxysteroid (17-beta) dehydrogenase 10
**Otc**	0.82	0.32	1.71	0.002029	ornithine transcarbamylase
**Cisd1**	0.53	0.38	2.35	0.001166	CDGSH iron sulfur domain 1
**Kynu**	0.62	0.46	1.98	3.95E-05	kynureninase (L-kynurenine hydrolase)
**Gcdh**	0.57	0.52	1.67	0.000211	glutaryl-Coenzyme A dehydrogenase
**Nipsnap1**	0.69	0.48	1.61	1.01E-06	4-nitrophenylphosphatase domain and non-neuronal SNAP25-like protein homolog 1 (C. elegans)
**Ndufs8**	0.62	0.51	1.47	0.001217	NADH dehydrogenase (ubiquinone) Fe-S protein 8
**Sfxn1**	0.76	0.55	1.77	1.61E-05	sideroflexin 1
**Dbi**	0.60	0.61	2.05	0.000292	diazepam binding inhibitor
**Cyp17a1**	0.24	0.17	2.59	6.85E-06	cytochrome P450, family 17, subfamily a, polypeptide 1
**Haao**	0.57	0.20	1.26	0.000502	3-hydroxyanthranilate 3,4-dioxygenase
**Prodh**	0.61	0.21	1.35	2.57E-05	proline dehydrogenase
**Gls2**	0.73	0.25	0.84	1.45E-03	glutaminase 2 (liver, mitochondrial)
**Ass1**	0.80	0.34	0.94	0.001664	argininosuccinate synthetase 1
**Mmab**	0.75	0.47	0.95	2.96E-05	methylmalonic aciduria (cobalamin deficiency) type B homolog (human)
**Macrod1**	0.95	0.50	0.64	0.001609	MACRO domain containing 1
**Cyb5r3**	0.60	0.56	1.04	0.000137	cytochrome b5 reductase 3
**Hsd3b2**	0.83	0.49	1.13	0.000502	hydroxy-delta-5-steroid dehydrogenase, 3 beta- and steroid delta-isomerase 2
**Ndufa3**	0.74	0.60	1.08	0.000687	NADH dehydrogenase (ubiquinone) 1 alpha subcomplex, 3
**Uqcrc1**	0.58	0.64	1.24	0.000902	ubiquinol-cytochrome c reductase core protein 1
**Hadhb**	2.04	3.17	0.99	0.000344	hydroxyacyl-Coenzyme A dehydrogenase/3-ketoacyl-Coenzyme A thiolase/enoyl-Coenzyme A hydratase (trifunctional protein), beta subunit
**Ung**	1.27	2.95	0.76	0.00021	uracil DNA glycosylase
**Mrps6**	1.20	1.91	0.92	0.002234	mitochondrial ribosomal protein S6
**Slc25a33**	2.60	2.43	0.38	0.000271	solute carrier family 25, member 33
**Hsp90ab1**	1.69	2.32	0.47	0.001625	heat shock protein 90kDa alpha (cytosolic), class B member 1
**Cyba**	1.20	2.00	0.68	7.33E-05	cytochrome b-245, alpha polypeptide

a FC_SQ: fold change in gene expression after peripheral (subcutaneous) leptin administration as compared to vehicle subcutaneous treatment.

b FC_ICV: fold change in gene expression after central (intracerebroventricular) leptin administration as compared to vehicle intracerebroventricular treatment.

c FC_ob/B6: fold change in gene expression in vehicle treated *ob/ob* mice as compared to the expression in B6 mice.

d adj.P.Val: adj. p-value of regression analysis performed to test for a linear association between gene expression levels and amount of weight loss.

#### Lipid metabolic process

We found 26 genes differentially expressed under lipid metabolic processes, out of which 19 genes were downregulated. Also, 12 of these genes could be mapped to lipid biosynthetic process, out of which 10 genes were downregulated. Leptin influences the rates of synthesis and degradation of lipids through its autocrine and paracrine actions. The molecular mechanism of leptin action in fatty acid metabolism and lipid biosynthesis has not yet been fully elucidated. We found that Scd1, Faah, Acot1, Rdh16 and Lss are downregulated by leptin treatment. The microsomal enzyme, stearoyl-CoA desaturase-1 (Scd1) catalyzes the biosynthesis of monounsaturated fatty acids from saturated fatty acids. In our study we found that leptin-deficient *ob/ob* mice have very high levels (∼5-fold) of Scd1 gene expression as compared to B6 control mice and after leptin replacement in *ob/ob* mice, the Scd1 gene expression is extremely downregulated (∼20-fold in SQ and ∼100-fold in ICV) as compared to their respective controls. Therefore, Scd1 appears to be a major player in the leptin mediated lipid biosynthesis and fatty acid metabolism. This finding is consistent with other studies which have shown that *ob/ob* mice with mutations in Scd1 gene are significantly less obese than *ob/ob* controls and have markedly increased energy expenditure. A significant proportion of metabolic effects of leptin are the result of inhibition of this enzyme [18]. On the other hand the Scd1^−/−^ mice in SV129 background are lean, resistant to diet-induced obesity, have increased insulin sensitivity, and increased metabolic rate [19].

#### Carboxylic acid metabolic process

Twenty-four downregulated genes including Gck, Faah, Otc, Arg1, Gls2, Ass1, Prodh and Aass, could be mapped to carboxylic acid metabolic pathways. Glucokinase (GcK) regulates rate-limiting reactions in glycolysis. Expression of Gck is regulated in the liver in response to fasting and feeding. We found that Gck is 2-fold and 5-fold downregulated after leptin administration. Hepatocyte nuclear factor 4 alpha (HNF4alpha) plays an important role in transcriptional regulation of GcK gene [20] and many genes expressed in the liver. We found that HNF-4 has a downward trend after leptin administration, but could not reach to statistical significance. HNF4alpha is also critical for urea homeostasis by direct regulation of the Otc gene [21]. Otc is a key enzyme in the urea cycle to detoxify ammonium produced from amino acid catabolism. Here, we found that Otc was also significantly downregulated after leptin replacement.

#### Iron ion binding

In this study, we found that 12 genes could be annotated to the iron ion binding group. The 9 downregulated genes were Cisd1, Haao, Cyp17a1, Dpyd, Hpd, Scd1, Cyp2c29, Ndufs8, and Sfxn1 and 3 upregulated genes of this group were Cyba, Hpx and, Slc40a1. There is a well established link between obesity and iron metabolism. *Ob/ob* mice have higher iron absorption as compared to lean mice [22]. There is evidence in the literature that insulin resistance is associated with hepatic iron overload. A recent study found a role of Hepacidin expression in metabolic syndrome and hepatic iron overload associated with insulin resistance [23]. Interestingly, in our study, we also found that expression of hepcidin antimicrobial peptide 2 (Hamp2) was 3-fold and 28-fold downregulated after leptin treatment.

#### Glutathione S Transferases

Glutathione is a major endogenous anti-oxidant for the cell. We found that three of the glutathione S-transferases were significantly overexpressed in *ob/ob* mice as compared to B6 (Gsta1: 41-fold, Gsta2: 8-fold, Gstt3: 3-fold). Also, leptin replacement caused the downregulation of Gsta1, Gsta2, Gsta3, Gstt1 and Gstt3 in *ob/ob* mice. Reactive oxygen species (ROS) production by mitochondria plays a critical role in many physiological processes and therefore over quenching of ROS in *ob/ob* mice may result in pathological conditions. A recent study has shown that mice lacking one of the key enzymes involved in the elimination of physiological ROS, glutathione peroxidase 1 (Gpx1), were protected from high-fat-diet-induced insulin resistance [24].

#### Genes involved in adipocyte proliferation and differentiation

It is well known that adipocyte proliferation and differentiation plays a critical role in obesity. Leptin causes alterations in the expression of many genes which inhibit adipocyte differentiation. The expression of Igfbp2 gene, which negatively regulates the biological activity of IGF1, was 6× (SQ) and 16× (ICV) upregulated after leptin replacement. Igfbp2 is almost 17× downregulated in *ob/ob* mice as compared to B6. Another mitochondrial gene, Cisd1 was 2.4× upregulated in *ob/ob* mice as compared to B6 and corrected back to normal by leptin. This protein encodes for MitoNEET protein which exists in low levels in preadipocytes, and its expression increases exponentially in differentiated adipocytes. Leptin has a mitogenic potential in liver cancer cells, which is mediated through the induction of Mat2a and Mat2b genes [25]. In this study, we found that expression of Mat2a was induced after both types of leptin treatment.

### Upregulated biological processes after leptin administration in *ob/ob* Mice

#### Lysosomal activity

We found that the cellular compartments enriched by upregulated genes were lysosome (5 genes), vacuole (6 genes), and vesicle (9 genes). Lysosomes contain enzymes which destroy organelles that have been damaged. Also, when adequate amount of food is not available for the cell, the organelle degradation and lysosomal activity is increased. A recent study has identified the previously unknown role of autophagy in regulating intracellular lipid stores [26]. Lysosomes continuously use portions of lipid droplets and process them for energy production during nutrient deprivation [26].

#### Immune system and inflammatory response

Leptin has structural similarities with the family of long-chain helical cytokines, including IL-6 and IL-12 and also has been found to play a critical regulatory role within the immune system that affects the course of inflammation [27]. Various studies have shown that immune cells respond to pharmacological doses of leptin. In this study we found that the immune system process and inflammatory response were enriched in the upregulated group of genes. The genes which were upregulated in this group are; C1qb, Cd44, Ccl4, Nupr1, Cyba, Fcer1g, Rmcs2, Fcer1g, Rps19, Plscr1, Ednrb, Cd44, Ung, Ctnnb1, Lmo2, and Hpx. A recent study has shown the function of leptin within T-cell polarization [28]. Utilizing the model of oxazolone-induced colitis, authors found that *ob/ob* mice were protected, whereas wild-type and leptin-reconstituted *ob/ob* mice developed colitis. This protection of Th1- as well as in Th2-dependent inflammation was associated with decreased expression of T-bet and GATA-3, in naive *ob/ob* T cells [28]. In this study, we also found that epidermal growth factor receptor (EGFR) expression was upregulated almost 2.5 times after leptin replacement in *ob/ob* mice. It is known that EGFR mediates both chemotaxis and proliferation in monocytes and macrophages and treatment with EGFR inhibitor decreases the protein expression of TNF-alpha and IL-6, and reduces subclinical inflammation in HFD-fed mice [29].

### Comparison of two types of gene expression changes after leptin treatment in *ob/ob* mice

Cluster analysis revealed that 214 differentially expressed genes could be sub-divided into two major groups: (A) 116 genes of Cluster-1 and Cluster-2, which have different expression in ob/ob when compared to B6, and leptin treatment normalizes the expression in ob/ob towards B6; (B) 98 genes of Cluster-3 and Cluster-4, which have similar expression in ob/ob and B6 but are downregulated or upregulated after leptin treatment. These two groups of genes were separately analyzed and were mapped to cellular compartments and biological functions (Table S1). The enriched cellular compartments in both groups were mitochondrion (group A: 19 genes; group B: 14 genes), cytosol (group A: 10 genes; group B: 10 genes) and endoplasmic reticulum (group A: 11 genes; group B: 11 genes). However, the plasma membrane (6 genes) and vacuole/lysosome (5 genes) were only enriched in group B. We have listed the comparison of enriched biological processes of each group in Table S1. Both groups are enriched in many metabolic processes whereas major catabolic processes are mainly enriched in group A. We also found that steroid metabolic process is only enriched in Group B and the genes involved in this process are Gpsn2, Cyb5r3, Lss, Cyp17a1, Pcsk9, Mvd, Hsd3b2.

#### Network Analysis

**Figure 5:**
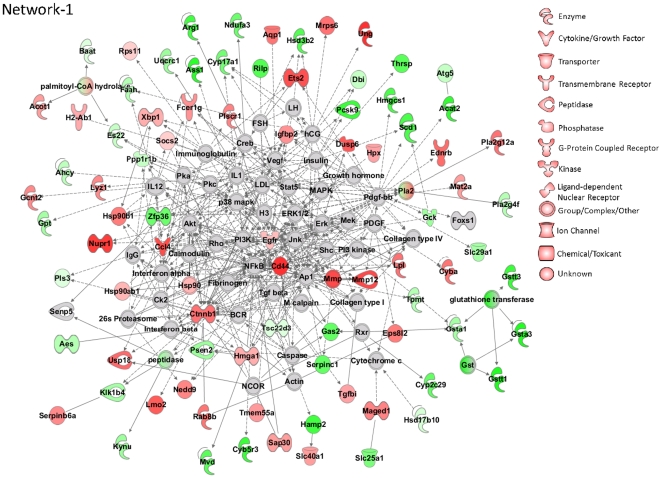
Top scoring network obtained from network analysis of differentially expressed genes. Ingenuity Pathway Analysis software was used to construct and visualize molecular interaction networks.

Differentially expressed genes were further analyzed using Ingenuity Pathway Analysis software (IPA; Ingenuity Systems, Mountain View, CA; www.ingenuity.com) to construct and visualize molecular interaction networks. Three high scoring networks could be identified. Network-1 (Figure 5) is enriched in lipid metabolism and cell-cell signaling. EGFR (which is upregulated after leptin treatment) is one of the central hub genes of this network. Also a lot of genes involved in this network are connected through important kinases. Expression of these kinases does not change after leptin replacement, but these might be mediators for the signal transduction between the genes which respond to leptin treatment. The transcriptional regulators in the network are Ets2, Xbp1, Sap30, Nupr1, Hmga1, Zfp36, Maged1 and Cttnb1. Network-2 and 3 are available in supplementary material (File S4: Networks.pdf). Network-2 is enriched in lipid metabolism and cell cycle and involves many cytokines. Network-3 is enriched in cell morphology and most of the genes in this network are connected through Hnf4a, Ifng and Htt.

## Discussion

Despite the fact that leptin has shown exciting results in the mouse models, the success rate of leptin for the reduction of body weight in humans is very low. This is due mainly to the fact that a very small fraction of the obese human population is leptin deficient, and the most common forms of obesity are associated with high leptin levels [9]. In such cases, most obese patients are leptin resistant and fail to respond effectively to exogenous leptin [8]. Several defects may contribute to the leptin resistant state, including defective leptin transport across the blood-brain barrier, reduced leptin receptor expression, defects in leptin signal transduction, or the induction of feedback inhibitors [10] [7]. However, if leptin resistance could be by-passed and genes/pathways which respond to leptin treatment could be regulated directly, new therapeutic strategies for the treatment of obesity may be possible. In this study, we determined the global hepatic gene expression profiles after peripheral and central leptin treatment. We found that the effect of central treatment on weight loss was significantly better than peripheral treatment; which confirms that leptin action on weight loss is primarily controlled by a central pathway via hypothalamic relay. We found 214 genes significantly correlated with leptin-mediated weight loss.

Obesity results from the imbalance of energy intake and energy utilization. When energy intake exceeds energy expenditure, excess energy is stored as triglycerides in lipid droplets. Conversely during nutrient deprivation, stored triglycerides are hydrolyzed into fatty acids and converted back to energy. Therefore, metabolic pathways are key regulators of energy balance. In this study, we found that genes which belong to metabolic, catabolic and biosynthetic pathways in mitochondria are highly expressed in *ob/ob* mice when compared with the B6 baseline expression. However, leptin replacement results in the downregulation of these pathways back to normal. The Stearoyl-CoA Desaturase 1 (Scd1), which is a lipogenic enzyme involved in fatty acid synthesis and oxidation, is overexpressed in the livers of *ob/ob* mice as compared to B6 and is downregulated after leptin replacement. This is consistent with the results of previous studies which have shown that Scd1 deficient mice are protected against obesity and insulin resistance [18], [19]. Also in humans, genetic variations in the Scd1 gene were found to be associated with body fat deposition and insulin sensitivity [30]. Some recent studies have used pharmacological inhibition of Scd1 for treatment of obesity and insulin resistance [31], [32]. Glucokinase (GcK) phosphorylates glucose to produce glucose-6-phosphate and therefore regulates glucose metabolism. We found that expression of this gene is negatively correlated with leptin-mediated weight loss in *ob/ob* mice. Long-term overexpression of Gck increases hepatic lipogenesis and circulating lipids, which leads to insulin resistance [33].

Weight loss also has been linked to GH-IGF1 axis in the literature, which has a critical role in the proliferation and differentiation of adipocytes [34]. IGF-binding proteins (IGFBPs) modulate bioavailability of IGF. In this study we found that Igfbp2 was highly underexpressed in livers of *ob/ob* mice but was normalized after leptin replacement. Recent studies have shown that overexpression of Igfbp2 protects against the development of obesity and improves insulin sensitivity [35]. Treatment with recombinant Igfbp2 impairs 3T3-L1 differentiation and hence adipogenesis [35]. Elevated insulin and body fat have been shown to be associated with decreased Igfbp1 and Igfbp2 levels cross-sectionally [36]. A recent study observed markedly lower expression of Igfbp2 in liver from morbidly obese women [37]. Such studies provide an impetus for investigating the effects of Igfbp2 for treatment of obesity. However, studies have also shown that Igfbp2 is overexpressed in a wide spectrum of cancers and IGF-independent effects of Igfbp2 are emerging [38]. Igfbp2 expression was significantly higher in breast cancer tissue compared with benign breast tissue and Igfbp2 inhibition attenuated the associated aggressive phenotype of breast cancer cells both in vitro and in vivo [39]. Overexpression of Igfbp2 has also been correlated with glioblastoma [40], [41] and lymph node metastasis in patients with invasive breast carcinomas [42]. These findings suggest that further studies are required to evaluate IGF-dependent and -independent functions of Igfbp2 before choosing Igfbp2 as a therapeutic target for treatment of obesity.

We found that Cisd1, the mitochondrial gene which encodes for the MitoNEET protein, is upregulated in livers of ob/ob mice as compared to B6 and could be normalized by leptin. MitoNEET is an iron-containing outer mitochondrial membrane protein that regulates oxidative capacity and is involved in the control of maximal mitochondrial respiratory rates [43]. This protein exists in low levels in preadipocytes, and its expression increases exponentially in differentiated adipocytes. Also, MitoNEET has been identified as a target for the thiazolidinedione class of diabetes drugs that may contribute to lipid lowering and/or antidiabetic actions [44].

Interestingly, the expression of five glutathione S-transferases (Gsta1, Gsta2, Gsta3, Gstt1, and Gstt3) were significantly downregulated after leptin administration. In an earlier study it was shown that Gsta3 expression is markedly induced during adipose conversion, which is virtually undetectable in confluent 3T3-L1 cells under basal conditions [45]. Inhibition of the 3T3-L1 adipogenic program demonstrated that Gsta3 expression is associated specifically with acquisition of the adipocytic phenotype.

One important finding of this study was the upregulation of hepatic lysosomal pathways, including vesicles and vacuole, after leptin treatment. This finding is consistent with a recent study which has identified the previously unknown role of autophagy in regulating intracellular lipid stores [26]. Starvation causes the induction of autophagy, which delivers intracellular proteins and organelles sequestered in double-membrane vesicles (autophagosomes) to lysosomes for degradation and use as an energy source. It was found that inhibition of autophagy in cultured hepatocytes and mouse liver increased triglyceride storage in lipid droplets.

In conclusion, this study identified key molecular pathways and downstream target genes involved in the leptin-mediated weight loss. Many of these genes have previously been shown to be associated with obesity; however, we also identified a number of novel target genes. Further detailed studies will be required to evaluate the possible use of these genes and the associated protein products as therapeutic targets for the treatment of obesity.

## Supporting Information

File S1 Top-214 Correlation plots.pdf. Regression plots of 214 genes significantly associated with leptin mediated weight loss. We used LIMMA to test for a linear association between gene expression levels and amount of weight loss (grams).
(0.93 MB PDF)

File S2 214_Gene_matrix.xls. Normalized gene expression data of 214 genes significantly associated with leptin mediated weight loss.
(0.52 MB XLS)

File S3 Gene Ontology.pdf. Biological processes, molecular functions and cellular compartments of hepatic genes regulated by leptin treatment.
(0.24 MB PDF)

File S4 Networks.pdf. Top molecular networks and their biological functions affected by leptin treatment. Network analysis was performed using Ingenuity Pathway Analysis software.
(1.17 MB PDF)

Table S1 Differentially expressed genes could be sub-divided into two major groups: 116 genes of Cluster-1 and Cluster-2 (group-A), and 98 genes of Cluster-3 and Cluster-4 (group-B). These two groups of genes were separately analyzed and were mapped to cellular compartments and biological functions.
(0.08 MB DOC)
