# Supplementary material for: Hepatic Gene Expression Profiling Reveals Key Pathways Involved in Leptin-Mediated Weight Loss in ob/ob Mice
Source: PLoS One. 2010 Aug 16;5(8):e12147. doi: 10.1371/journal.pone.0012147 (PMC2922341; doi:10.1371/journal.pone.0012147)
Supplement: File S1 — Top-214 Correlation plots.pdf. Regression plots of 214 genes significantly associated with leptin mediated weight loss. We used LIMMA to test for a linear association between gene expression levels and amount of weight loss (grams). (0.93 MB PDF) [file pone.0012147.s001.pdf]

Supplementary data: Regression plots of 214 genes significantly associated with leptin mediated weight loss.

We used LIMMA to test for a linear association between gene expression levels and amount of weight loss (grams), using the amount of weight loss in a linear model and testing for the significance of the regression coefficient for weight loss. We used the false discovery rate (FDR) adjust for multiple testing. B-statistics (the log of the odds of a gene showing either any association with weight loss) were calculated for each gene. Duplicate genes, when present, were removed and their expression levels averaged across the duplicates. A total of 221 probes representing 214 unique genes were found significantly correlated with weight loss.

Regression plots of 214 are shown below. Blue open circle is vehicle subcutaneous treatment (VEH_SQ); Blue filled circle is leptin subcutaneous treatment (LEP_SQ); Red open circle is vehicle intracerebroventricular treatment (VEH_ICV); Red filled circle is leptin intracerebroventricular treatment (LEP_ICV); Red crossed circle represents four animals in which the cannula may not have been in place or treatment did not work (LEP_ICVN). While analyzing the phenotype data, we found that there was no weight loss in these four animals. Sectioning of the brain could not confirm placement of the cannula in these animals. The expression of these animals is very similar to the vehicle treated animals.

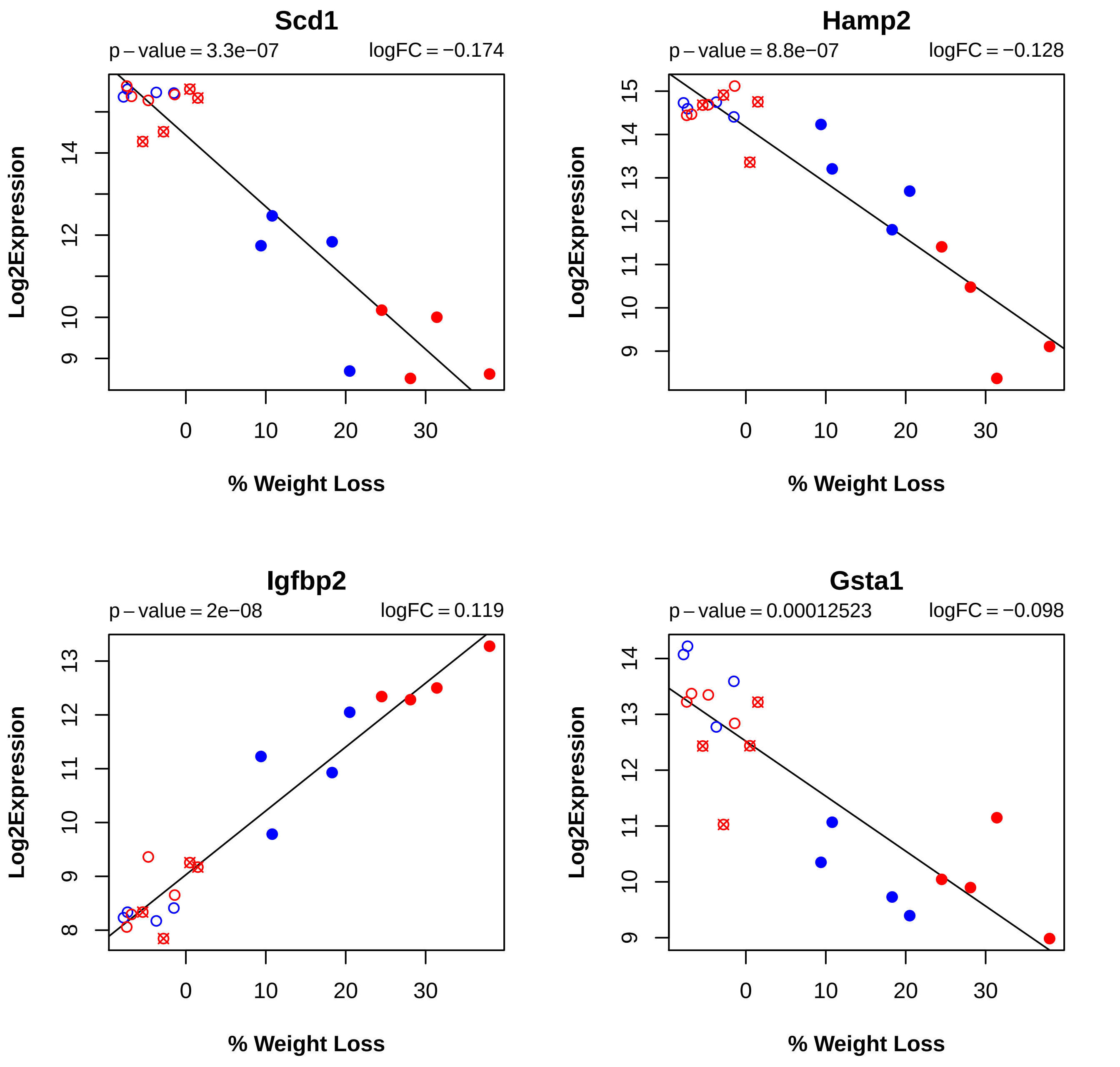

Scd1
p − value = 3.3e−07
logFC = −0.174
Log2Expression
% Weight Loss
Hamp2
p − value = 8.8e−07
logFC = −0.128
Log2Expression
% Weight Loss
Igfbp2
p − value = 2e−08
logFC = 0.119
Log2Expression
% Weight Loss
Gsta1
p − value = 0.00012523
logFC = −0.098
Log2Expression
% Weight Loss

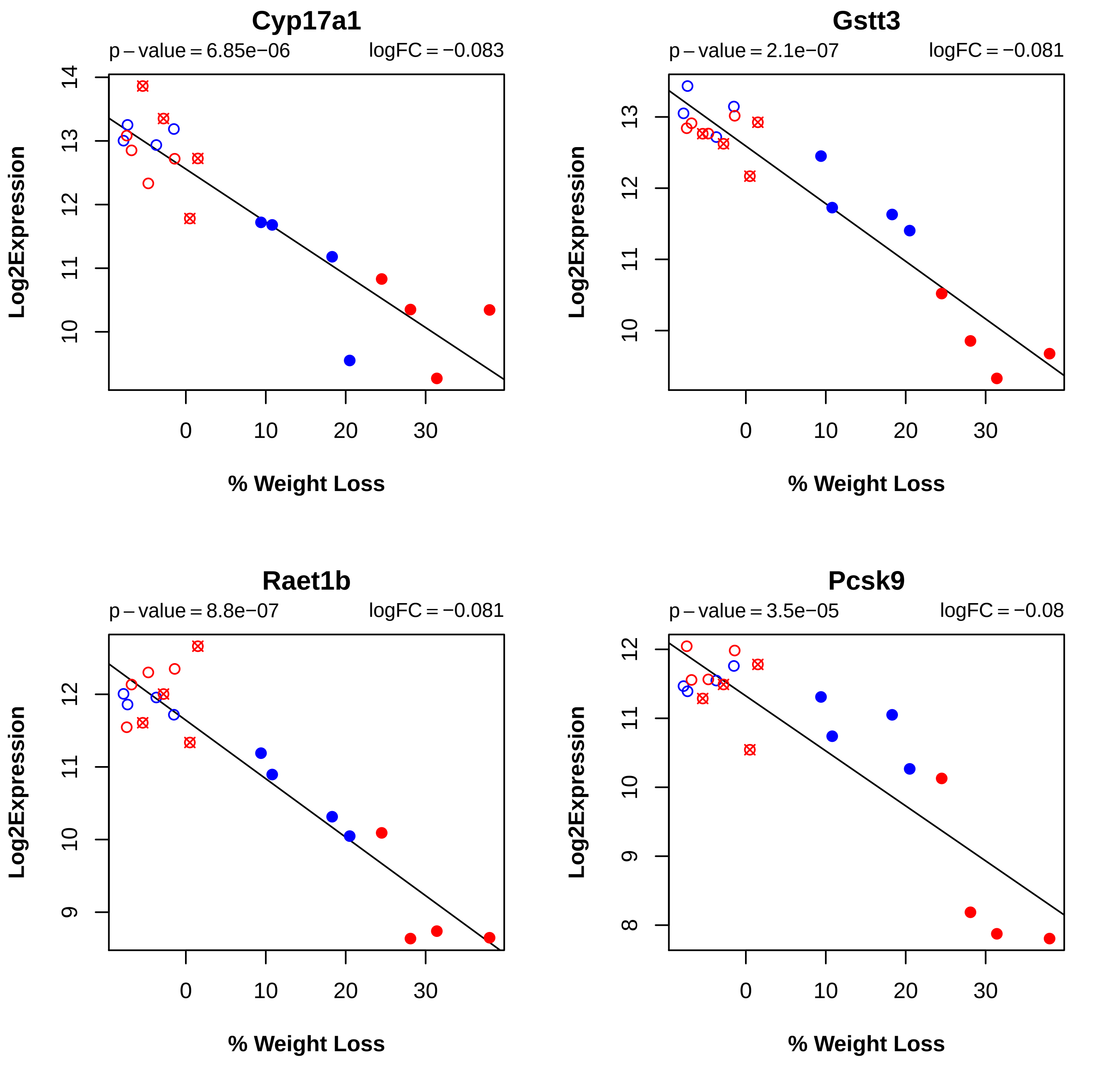
Cyp17a1
p − value = 6.85e−06
logFC = −0.083
Gstt3
p − value = 2.1e−07
logFC = −0.081
Raet1b
p − value = 8.8e−07
logFC = −0.081
Pcsk9
p − value = 3.5e−05
logFC = −0.08
Log2Expression
% Weight Loss
0
10
20
30

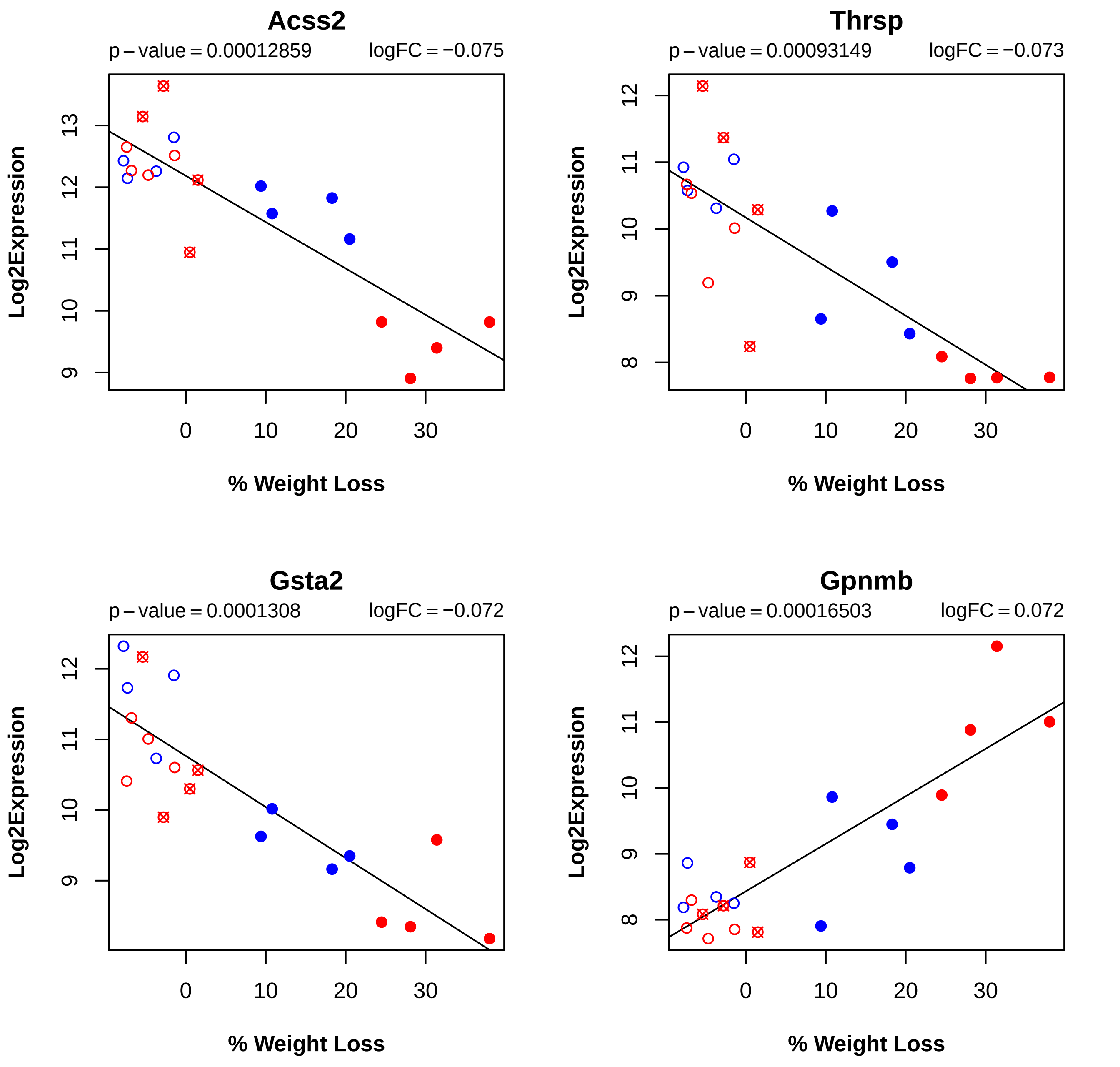

Acss2
p − value = 0.00012859
logFC = −0.075
Log2Expression
% Weight Loss
Thrsp
p − value = 0.00093149
logFC = −0.073
Log2Expression
% Weight Loss
Gsta2
p − value = 0.0001308
logFC = −0.072
Log2Expression
% Weight Loss
Gpnmb
p − value = 0.00016503
logFC = 0.072
Log2Expression
% Weight Loss

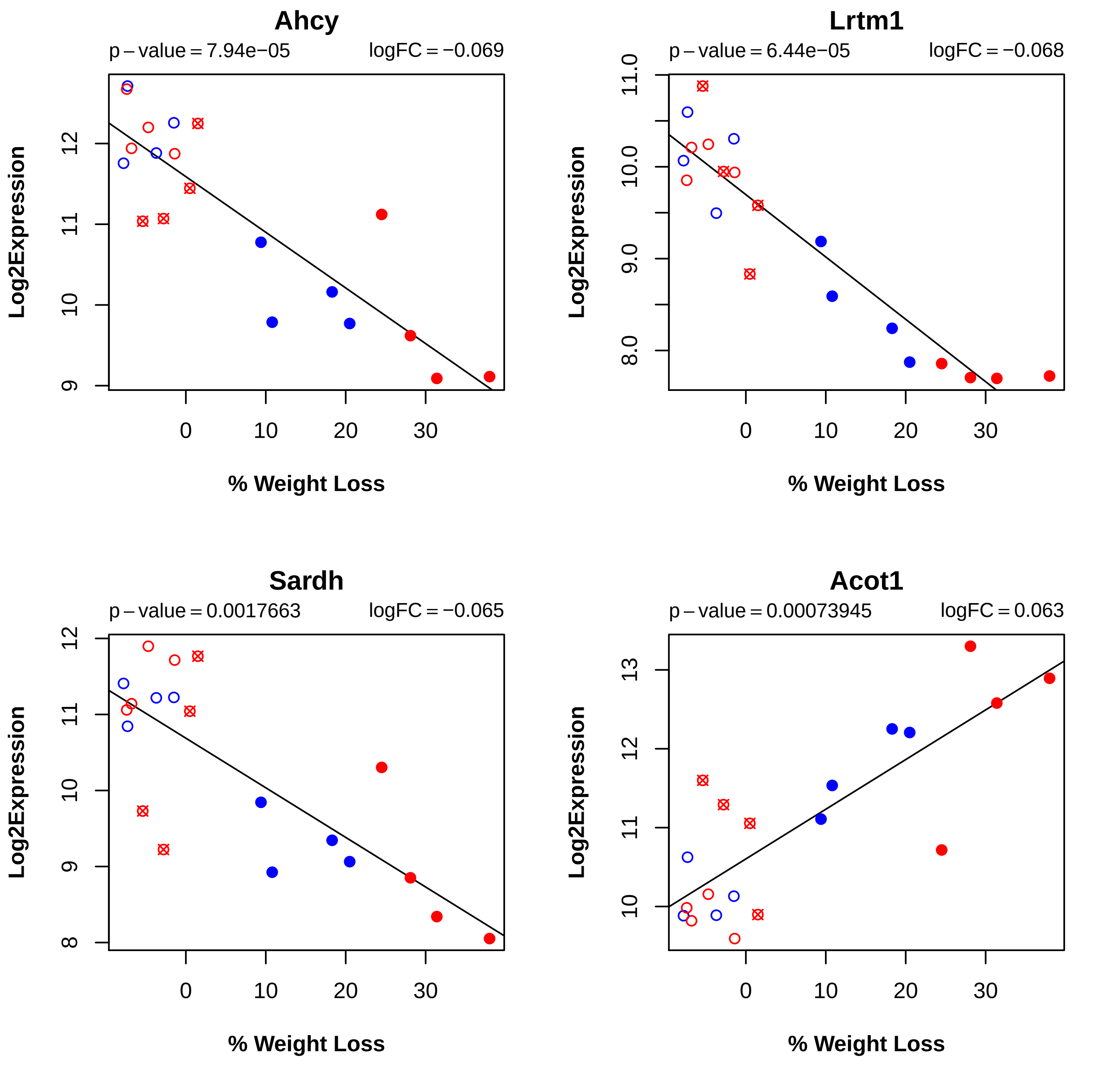

Ahcy
p – value = 7.94e−05
logFC = −0.069
Log2Expression
% Weight Loss
Lrtm1
p – value = 6.44e−05
logFC = −0.068
Log2Expression
% Weight Loss
Sardh
p – value = 0.0017663
logFC = −0.065
Log2Expression
% Weight Loss
Acot1
p – value = 0.00073945
logFC = 0.063
Log2Expression
% Weight Loss

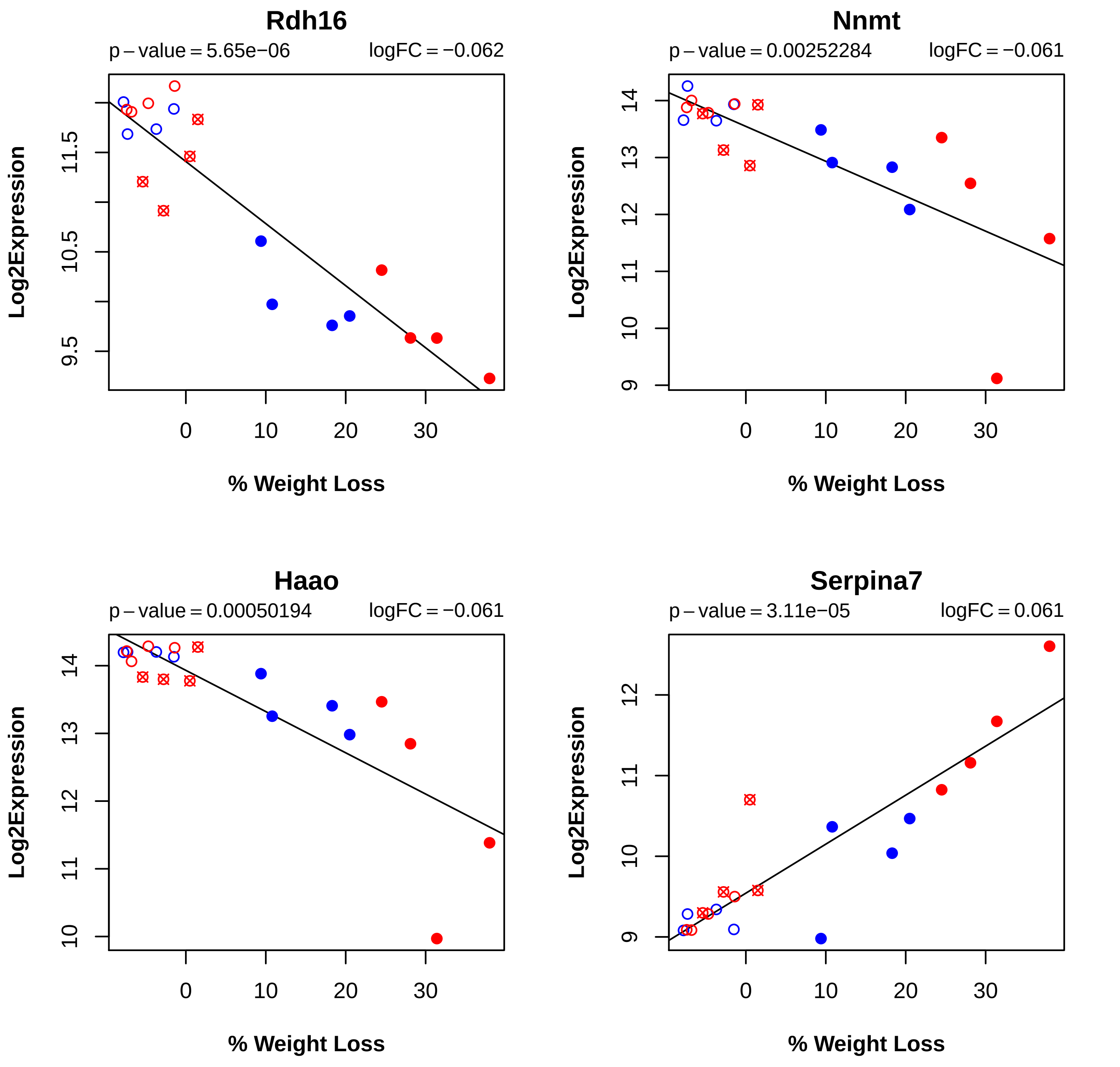

Rdh16
p – value = 5.65e−06
logFC = −0.062
Log2Expression
9.5
10.5
11.5
0
10
20
30
% Weight Loss
Nnmt
p – value = 0.00252284
logFC = −0.061
Log2Expression
9
10
11
12
13
14
0
10
20
30
% Weight Loss
Haao
p – value = 0.00050194
logFC = −0.061
Log2Expression
10
11
12
13
14
0
10
20
30
% Weight Loss
Serpina7
p – value = 3.11e−05
logFC = 0.061
Log2Expression
9
10
11
12
0
10
20
30
% Weight Loss

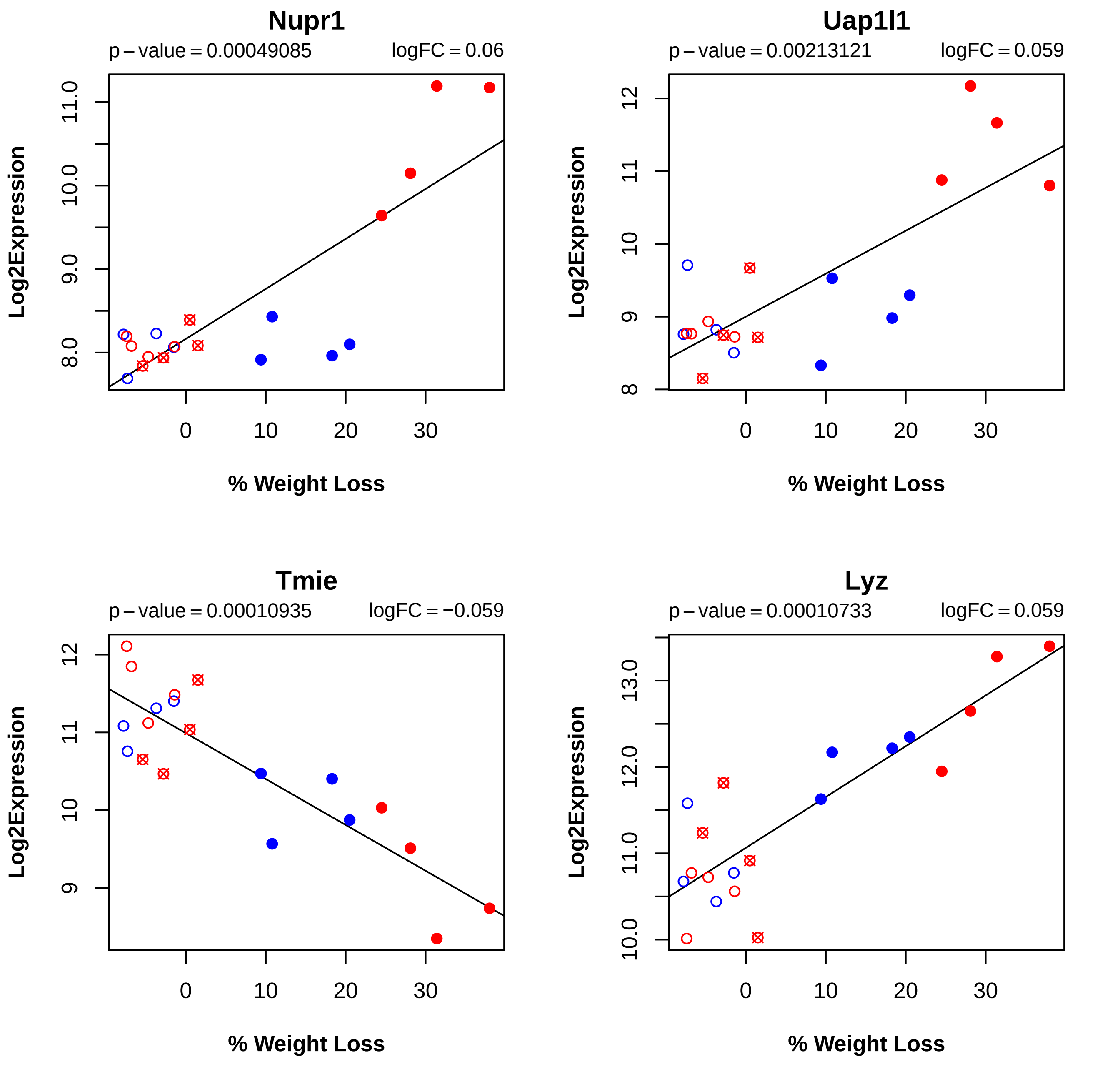

Nupr1
p – value = 0.00049085
logFC = 0.06
Log2Expression
% Weight Loss
8.0
9.0
10.0
11.0
0
10
20
30
Uap1l1
p – value = 0.00213121
logFC = 0.059
Log2Expression
% Weight Loss
8
9
10
11
12
0
10
20
30
Tmie
p – value = 0.00010935
logFC = −0.059
Log2Expression
% Weight Loss
9
10
11
12
0
10
20
30
Lyz
p – value = 0.00010733
logFC = 0.059
Log2Expression
% Weight Loss
10.0
11.0
12.0
13.0
0
10
20
30

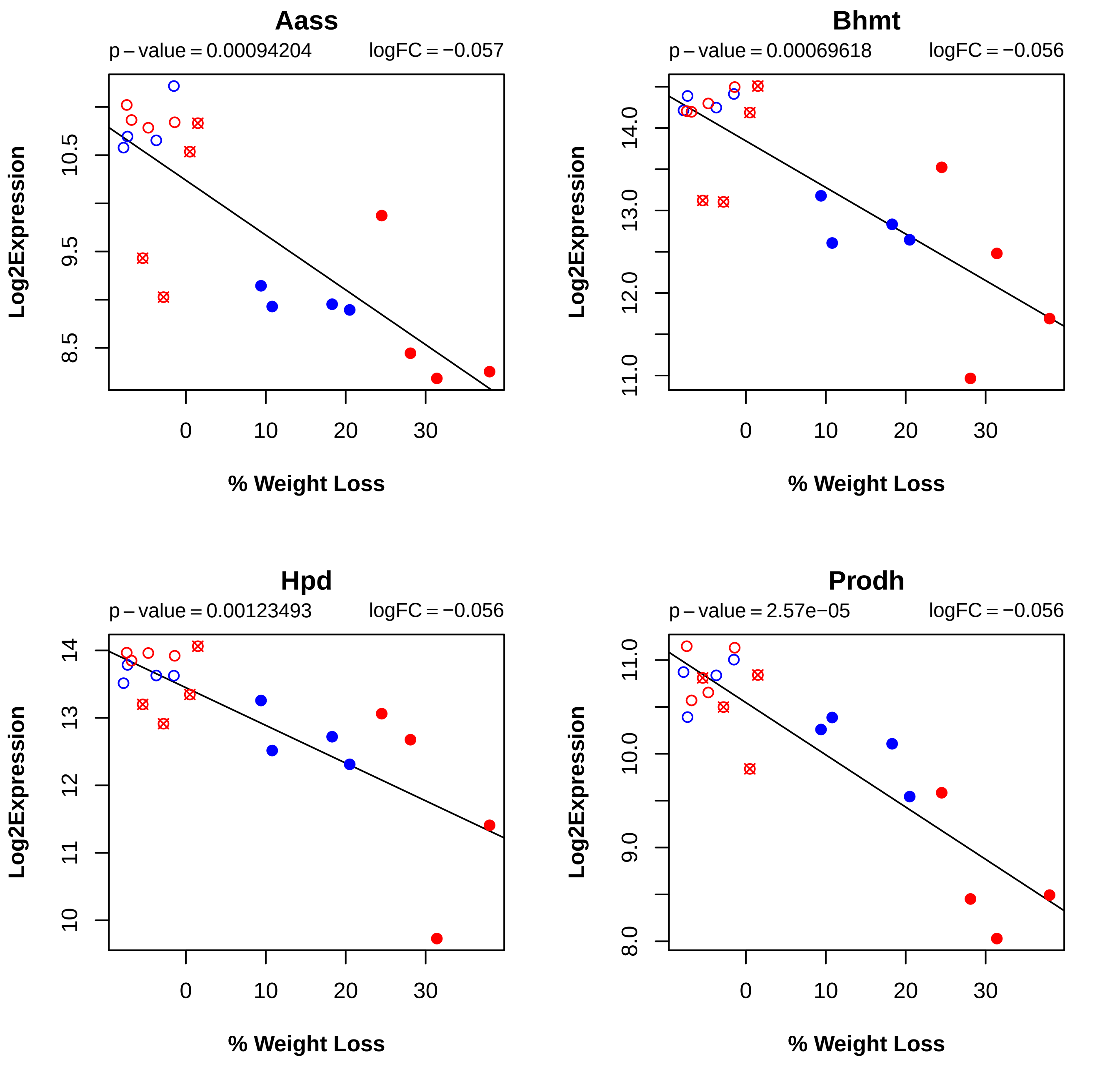
Aass
p − value = 0.00094204
logFC = −0.057
Log2Expression
% Weight Loss
Bhmt
p − value = 0.00069618
logFC = −0.056
Log2Expression
% Weight Loss
Hpd
p − value = 0.00123493
logFC = −0.056
Log2Expression
% Weight Loss
Prodh
p − value = 2.57e−05
logFC = −0.056
Log2Expression
% Weight Loss

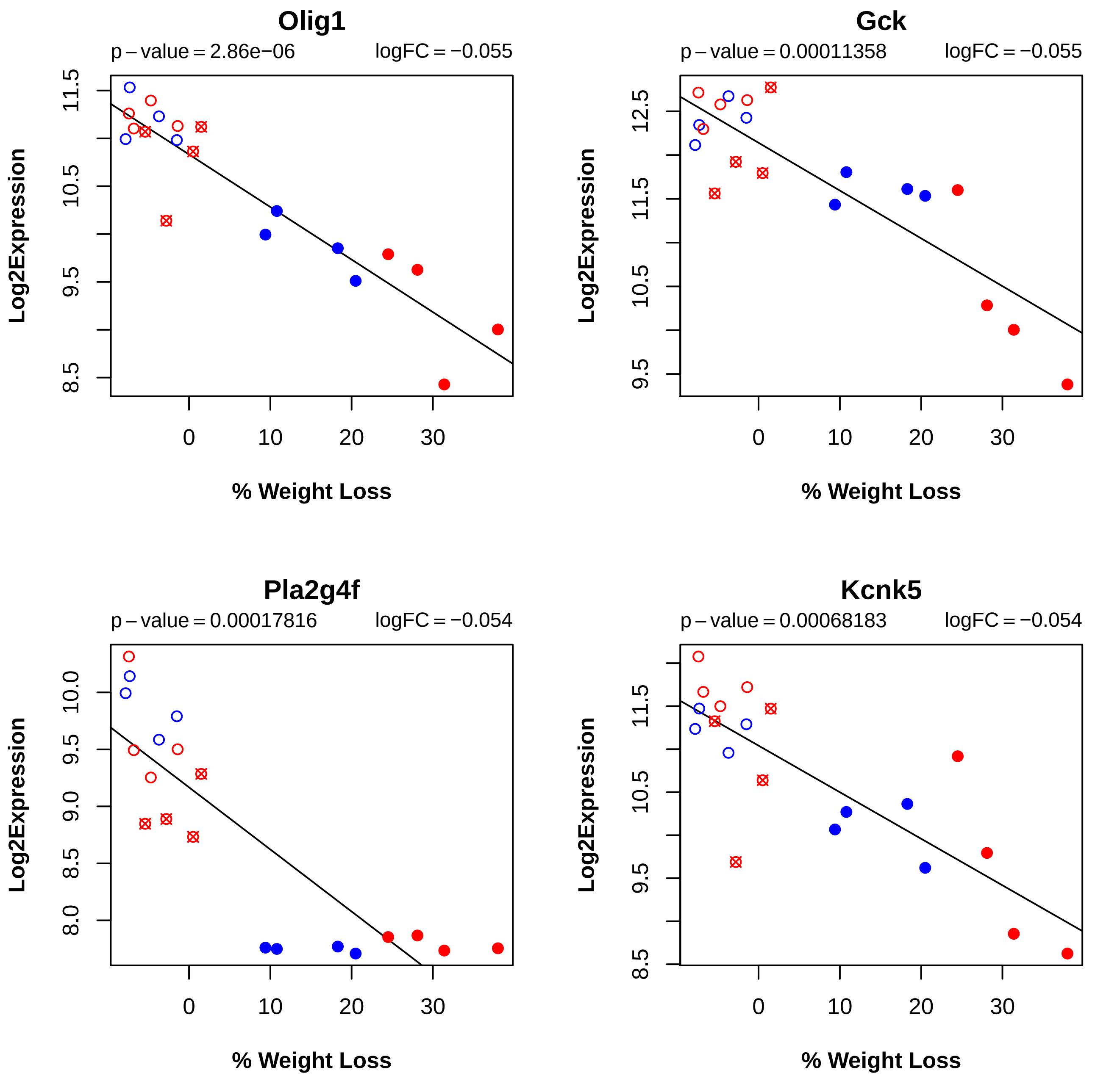
Olig1
p – value = 2.86e−06
logFC = −0.055
Log2Expression
11.5
10.5
9.5
8.5
0
10
20
30
% Weight Loss
Gck
p – value = 0.00011358
logFC = −0.055
Log2Expression
12.5
11.5
10.5
9.5
0
10
20
30
% Weight Loss
Pla2g4f
p – value = 0.00017816
logFC = −0.054
Log2Expression
10.0
9.5
9.0
8.5
8.0
0
10
20
30
% Weight Loss
Kcnk5
p – value = 0.00068183
logFC = −0.054
Log2Expression
11.5
10.5
9.5
8.5
0
10
20
30
% Weight Loss

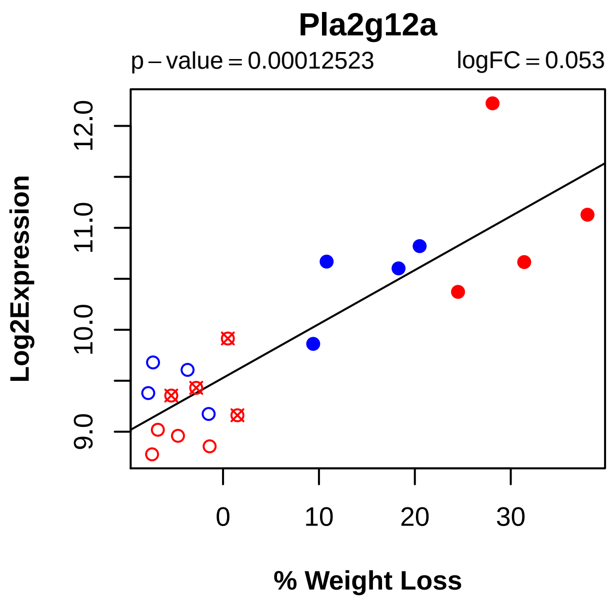

Pla2g12a
p – value = 0.00012523
logFC = 0.053
Log2Expression
% Weight Loss

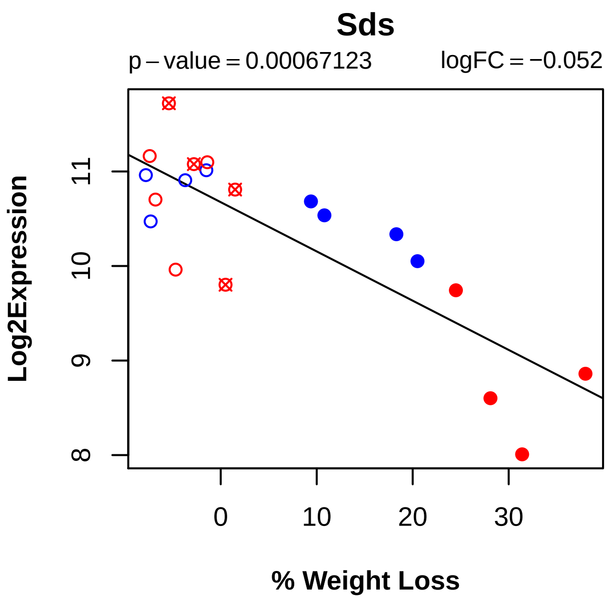

Sds
p – value = 0.00067123
logFC = −0.052
Log2Expression
% Weight Loss

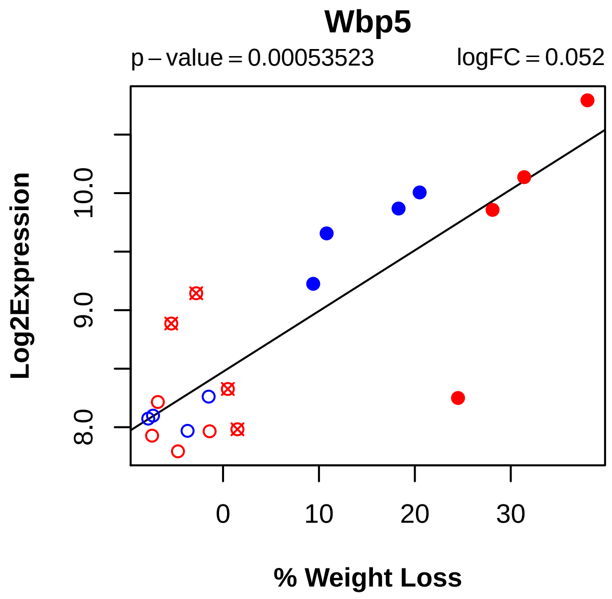

Wbp5
p – value = 0.00053523
logFC = 0.052
Log2Expression
% Weight Loss

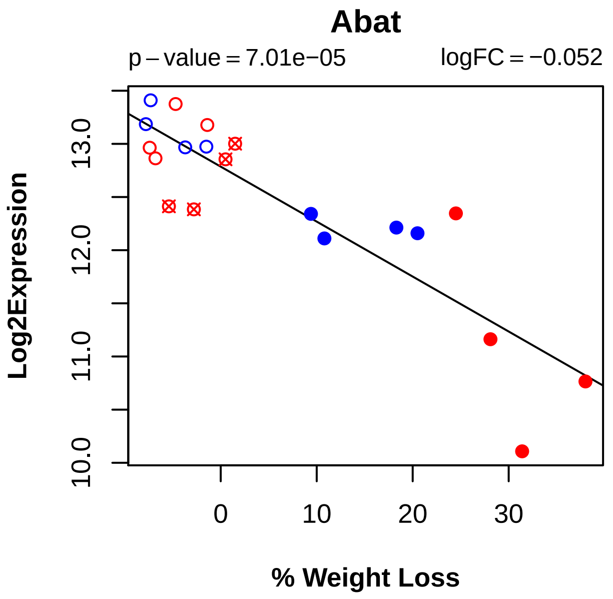

Abat
p – value = 7.01e−05
logFC = −0.052
Log2Expression
% Weight Loss

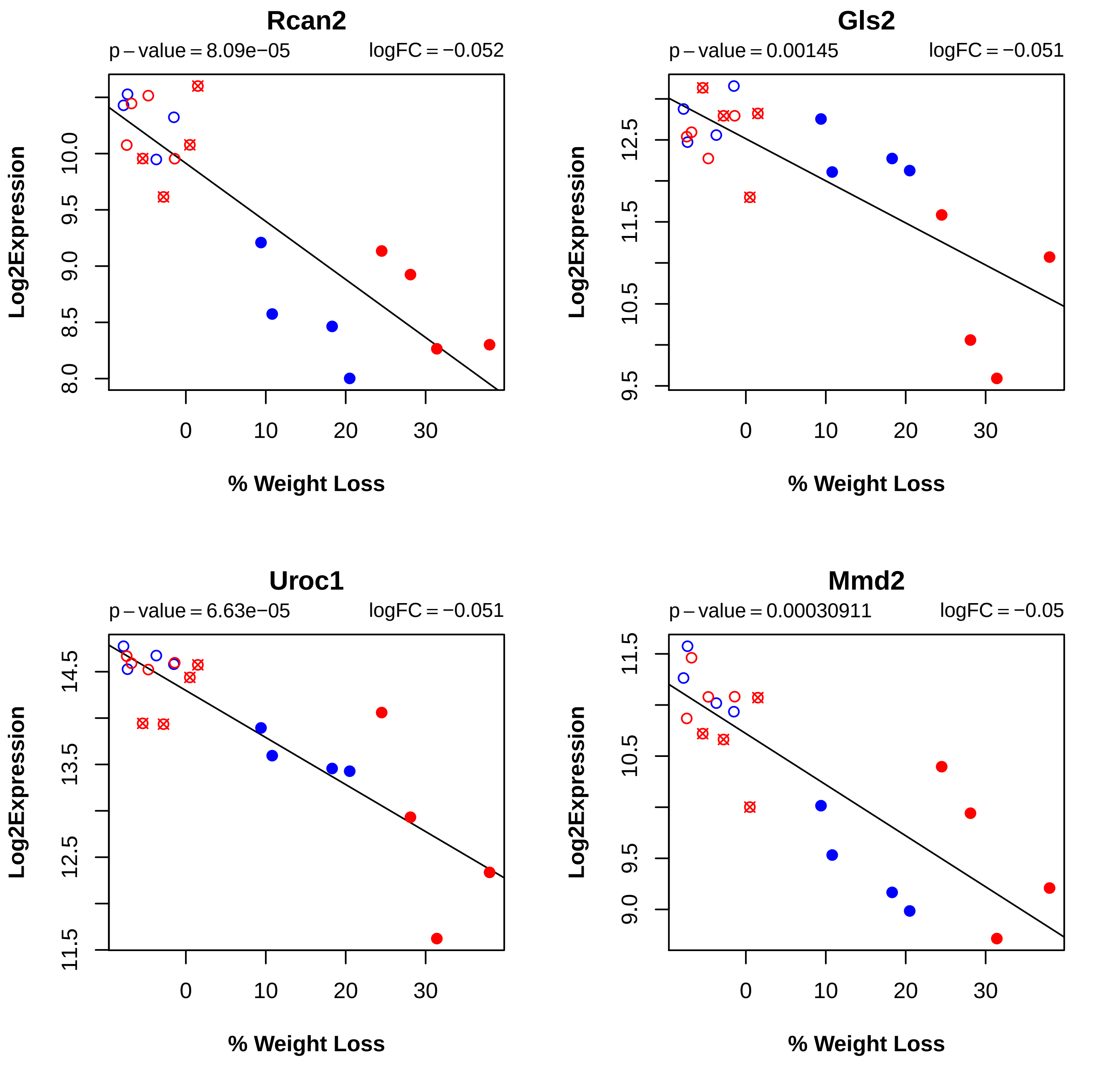
Rcan2
p – value = 8.09e−05
logFC = −0.052
Log2Expression
% Weight Loss
Gls2
p – value = 0.00145
logFC = −0.051
Log2Expression
% Weight Loss
Uroc1
p – value = 6.63e−05
logFC = −0.051
Log2Expression
% Weight Loss
Mmd2
p – value = 0.00030911
logFC = −0.05
Log2Expression
% Weight Loss

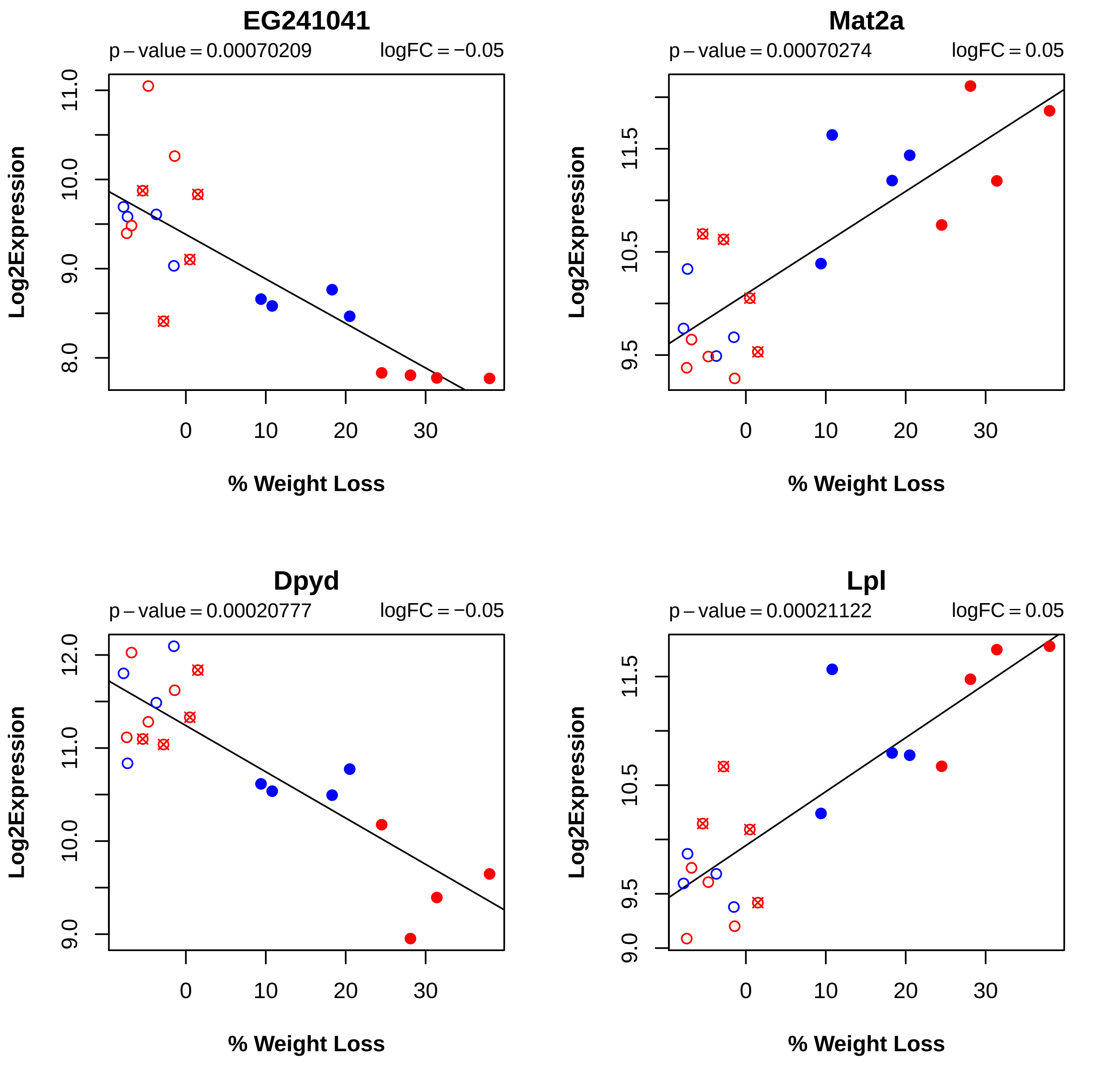

EG241041
p − value = 0.00070209
logFC = −0.05
Log2Expression
% Weight Loss
Mat2a
p − value = 0.00070274
logFC = 0.05
Log2Expression
% Weight Loss
Dpyd
p − value = 0.00020777
logFC = −0.05
Log2Expression
% Weight Loss
Lpl
p − value = 0.00021122
logFC = 0.05
Log2Expression
% Weight Loss

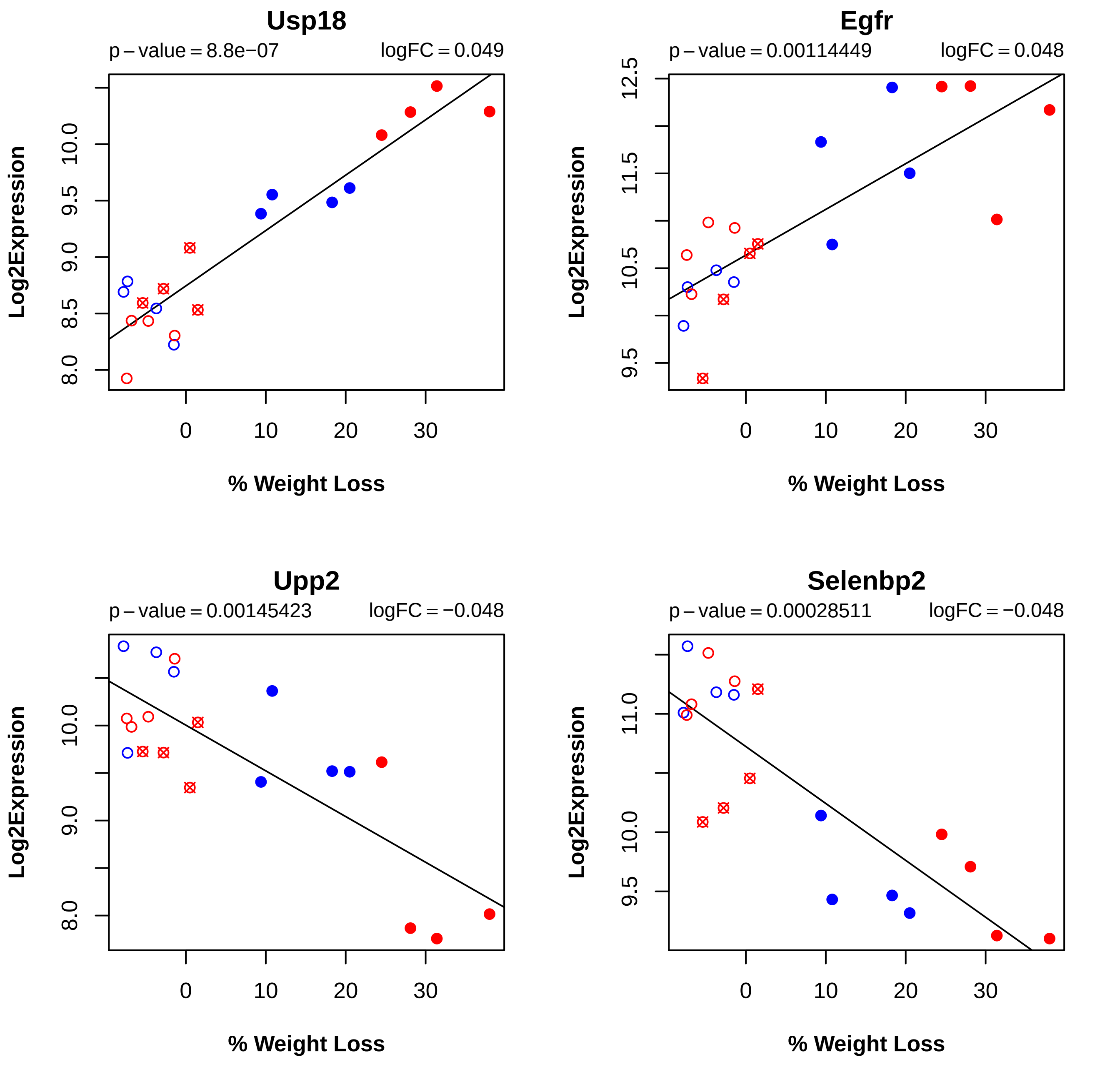
Usp18
p − value = 8.8e−07
logFC = 0.049
Log2Expression
% Weight Loss
Egfr
p − value = 0.00114449
logFC = 0.048
Log2Expression
% Weight Loss
Upp2
p − value = 0.00145423
logFC = −0.048
Log2Expression
% Weight Loss
Selenbp2
p − value = 0.00028511
logFC = −0.048
Log2Expression
% Weight Loss

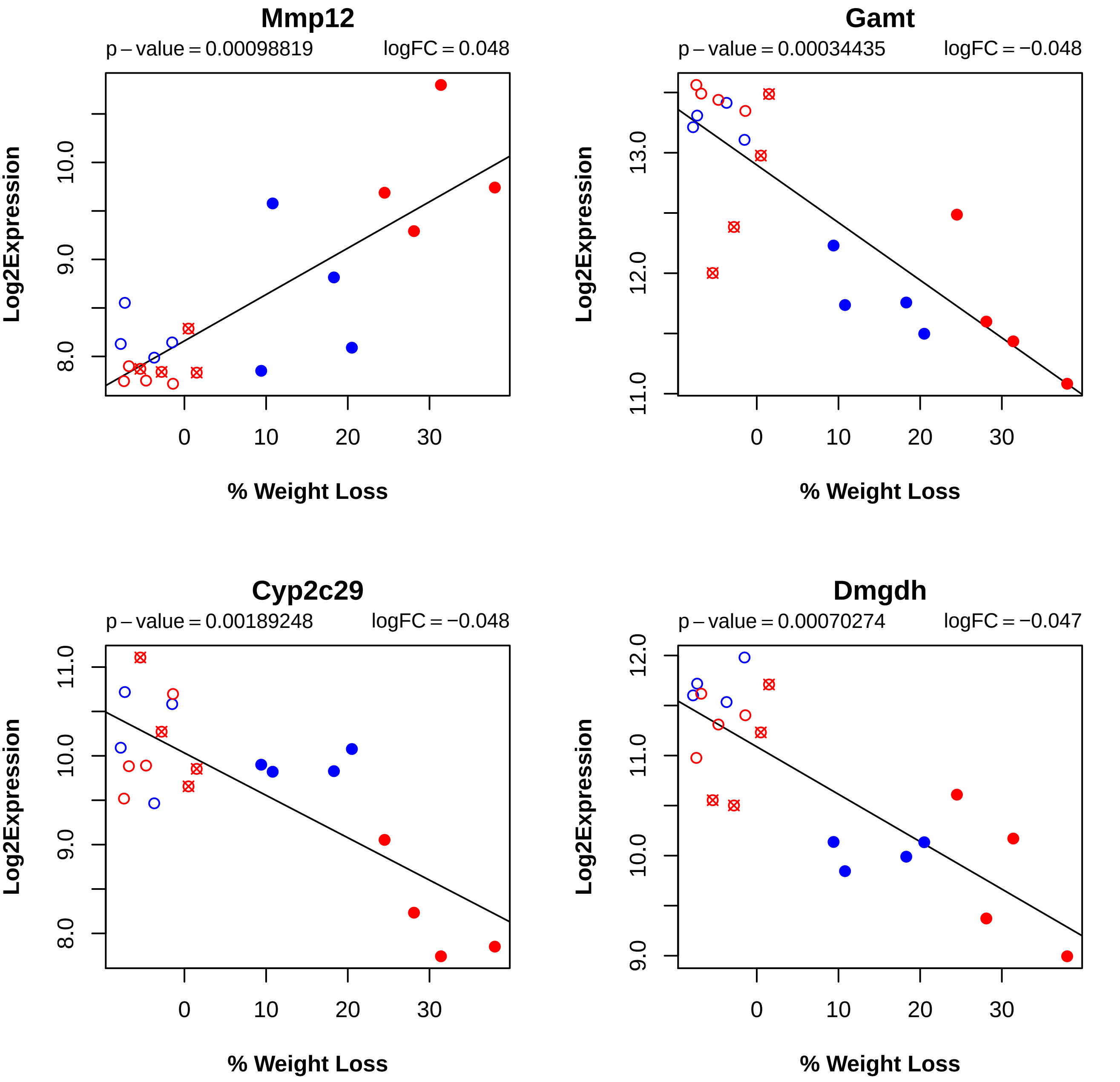
Mmp12
p – value = 0.00098819
logFC = 0.048
Log2Expression
% Weight Loss
Gamt
p – value = 0.00034435
logFC = −0.048
Log2Expression
% Weight Loss
Cyp2c29
p – value = 0.00189248
logFC = −0.048
Log2Expression
% Weight Loss
Dmgdh
p – value = 0.00070274
logFC = −0.047
Log2Expression
% Weight Loss

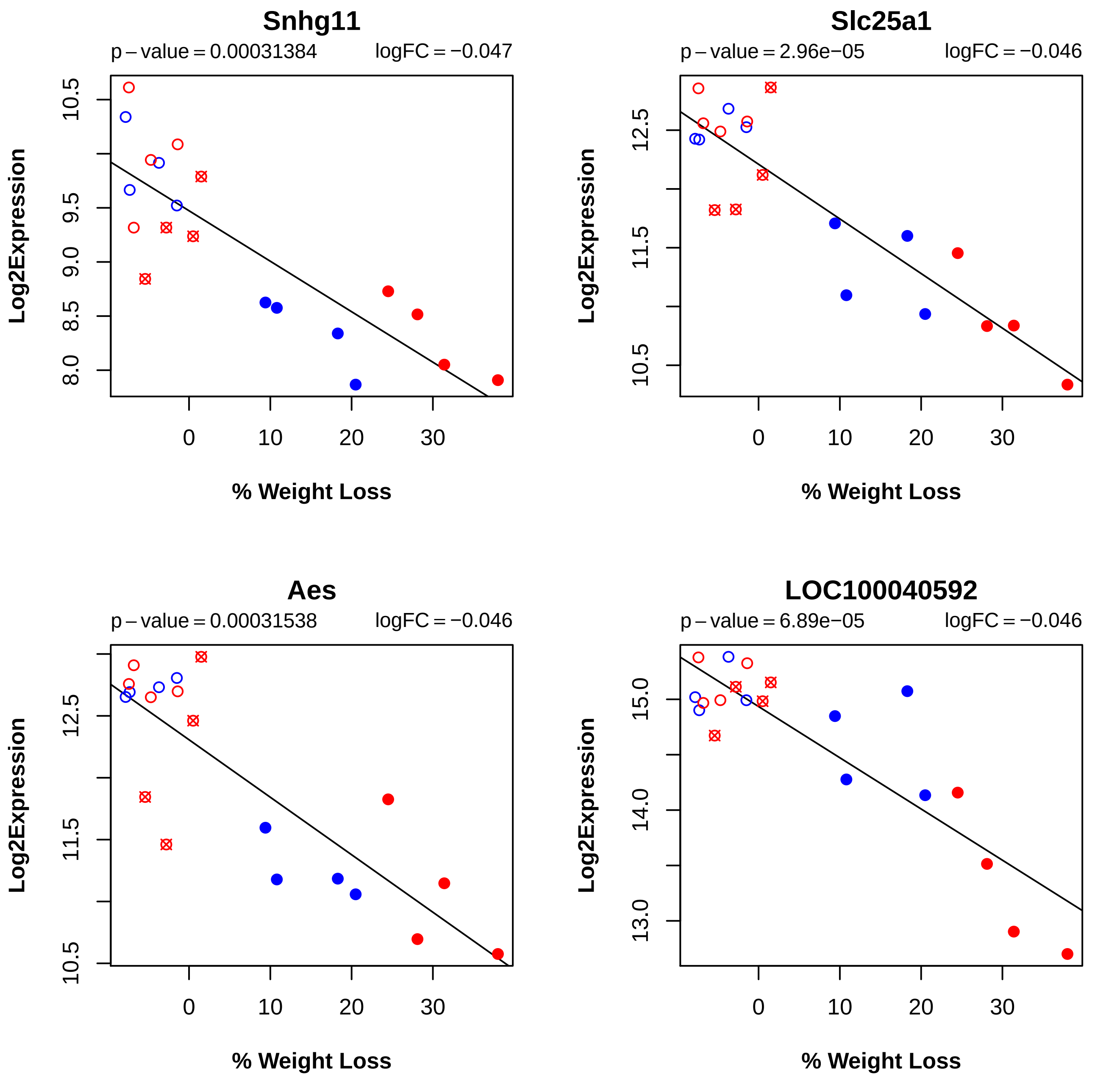
Snhg11
p – value = 0.00031384
logFC = −0.047
Log2Expression
% Weight Loss
Slc25a1
p – value = 2.96e−05
logFC = −0.046
Log2Expression
% Weight Loss
Aes
p – value = 0.00031538
logFC = −0.046
Log2Expression
% Weight Loss
LOC100040592
p – value = 6.89e−05
logFC = −0.046
Log2Expression
% Weight Loss

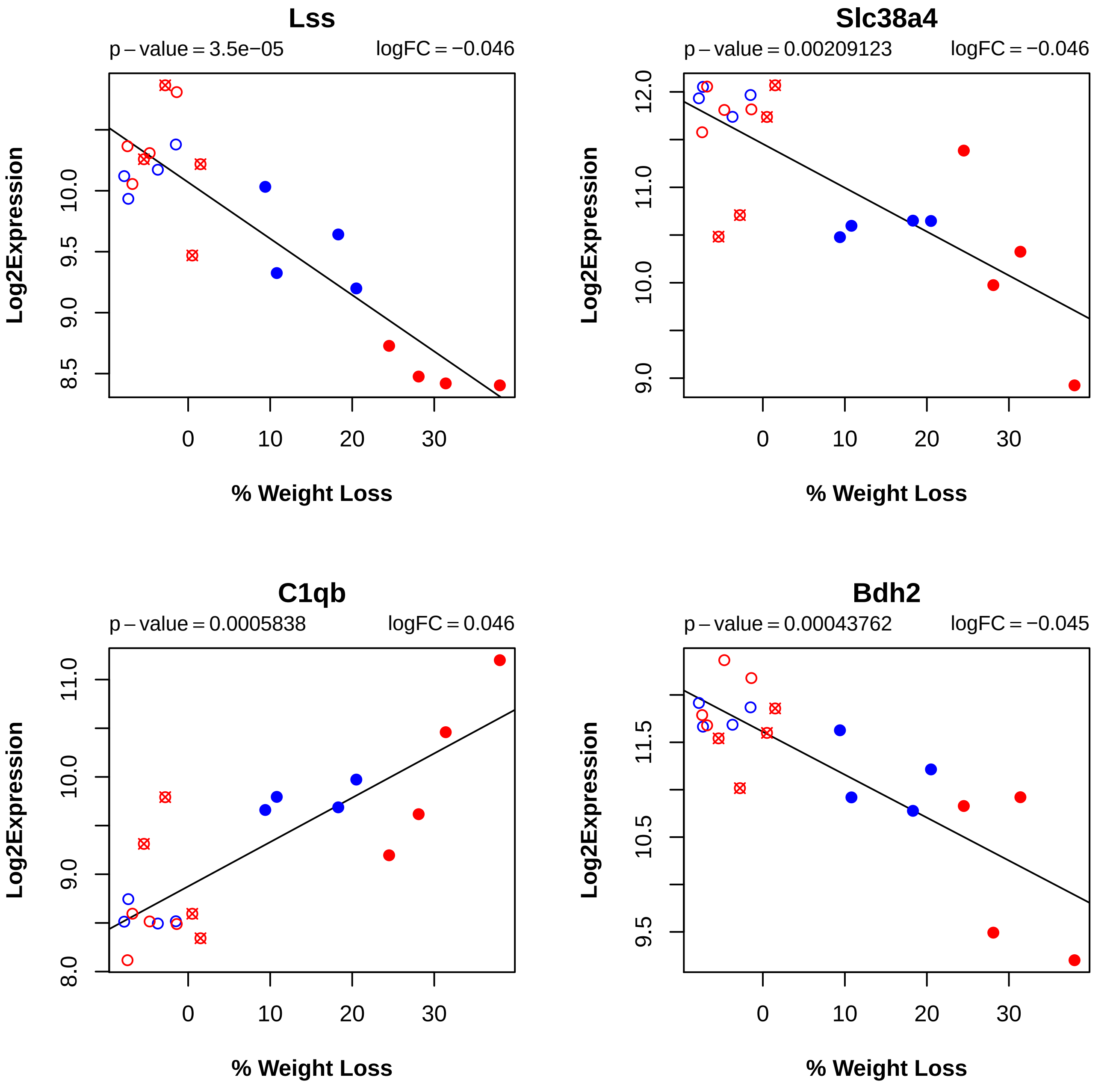
Lss
p – value = 3.5e−05
logFC = −0.046
Log2Expression
8.5
9.0
9.5
10.0
0
10
20
30
% Weight Loss
Slc38a4
p – value = 0.00209123
logFC = −0.046
Log2Expression
9.0
10.0
11.0
12.0
0
10
20
30
% Weight Loss
C1qb
p – value = 0.0005838
logFC = 0.046
Log2Expression
8.0
9.0
10.0
11.0
0
10
20
30
% Weight Loss
Bdh2
p – value = 0.00043762
logFC = −0.045
Log2Expression
9.5
10.5
11.5
0
10
20
30
% Weight Loss

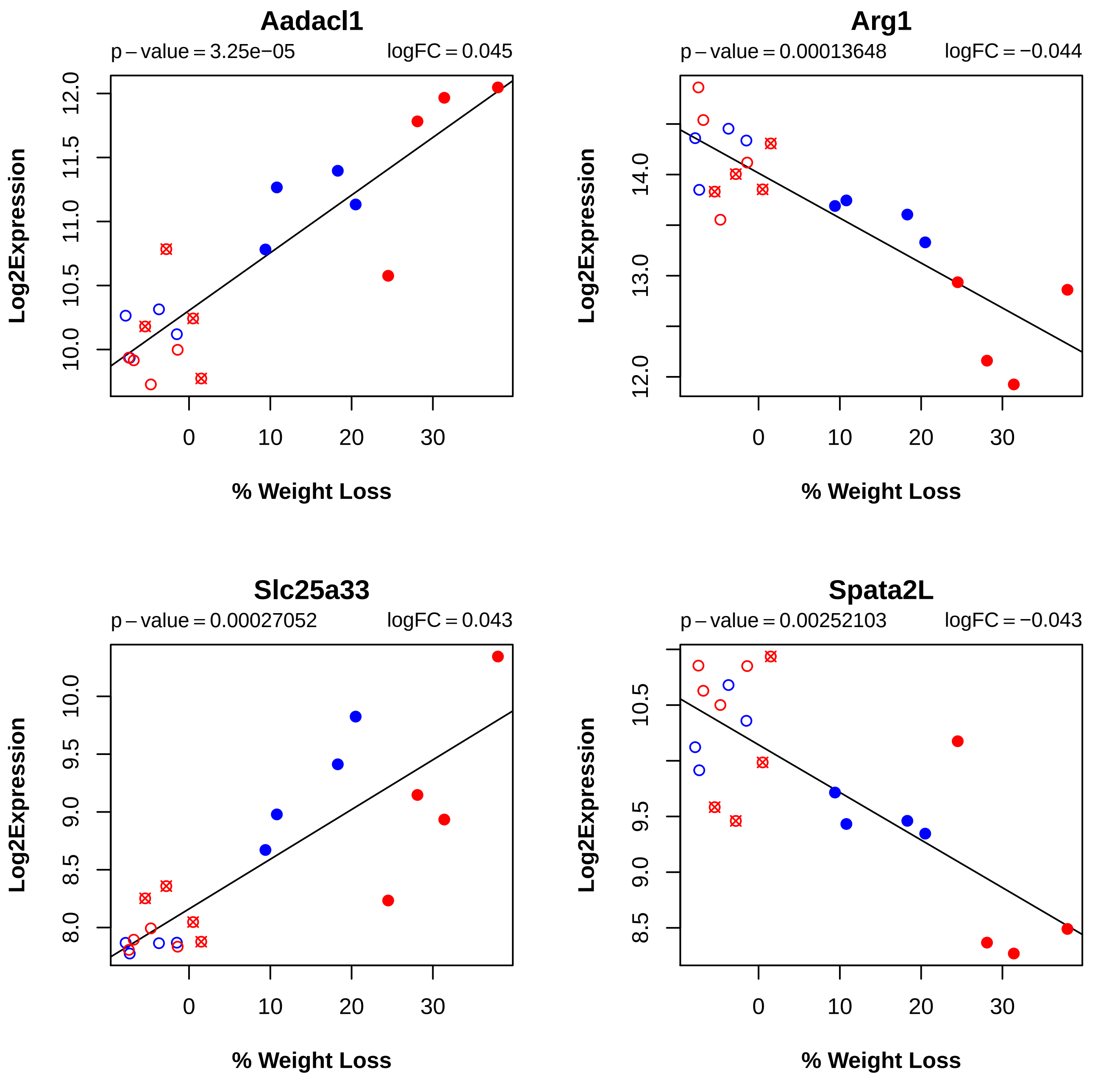
Aadacl1
p – value = 3.25e−05
logFC = 0.045
Log2Expression
% Weight Loss
Arg1
p – value = 0.00013648
logFC = −0.044
Log2Expression
% Weight Loss
Slc25a33
p – value = 0.00027052
logFC = 0.043
Log2Expression
% Weight Loss
Spata2L
p – value = 0.00252103
logFC = −0.043
Log2Expression
% Weight Loss

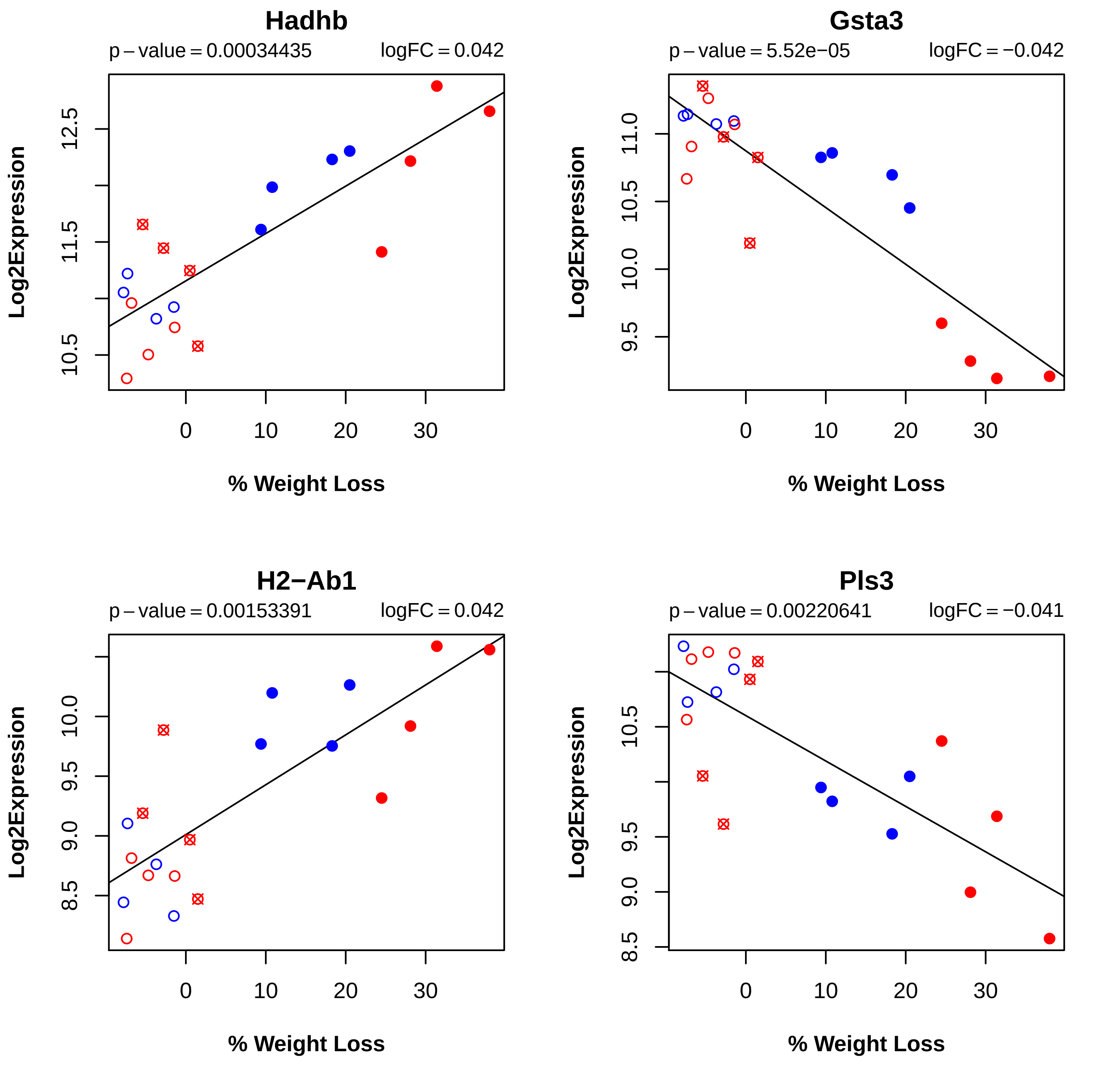

Hadhb
p − value = 0.00034435
logFC = 0.042
Log2Expression
% Weight Loss
Gsta3
p − value = 5.52e−05
logFC = −0.042
Log2Expression
% Weight Loss
H2−Ab1
p − value = 0.00153391
logFC = 0.042
Log2Expression
% Weight Loss
Pls3
p − value = 0.00220641
logFC = −0.041
Log2Expression
% Weight Loss

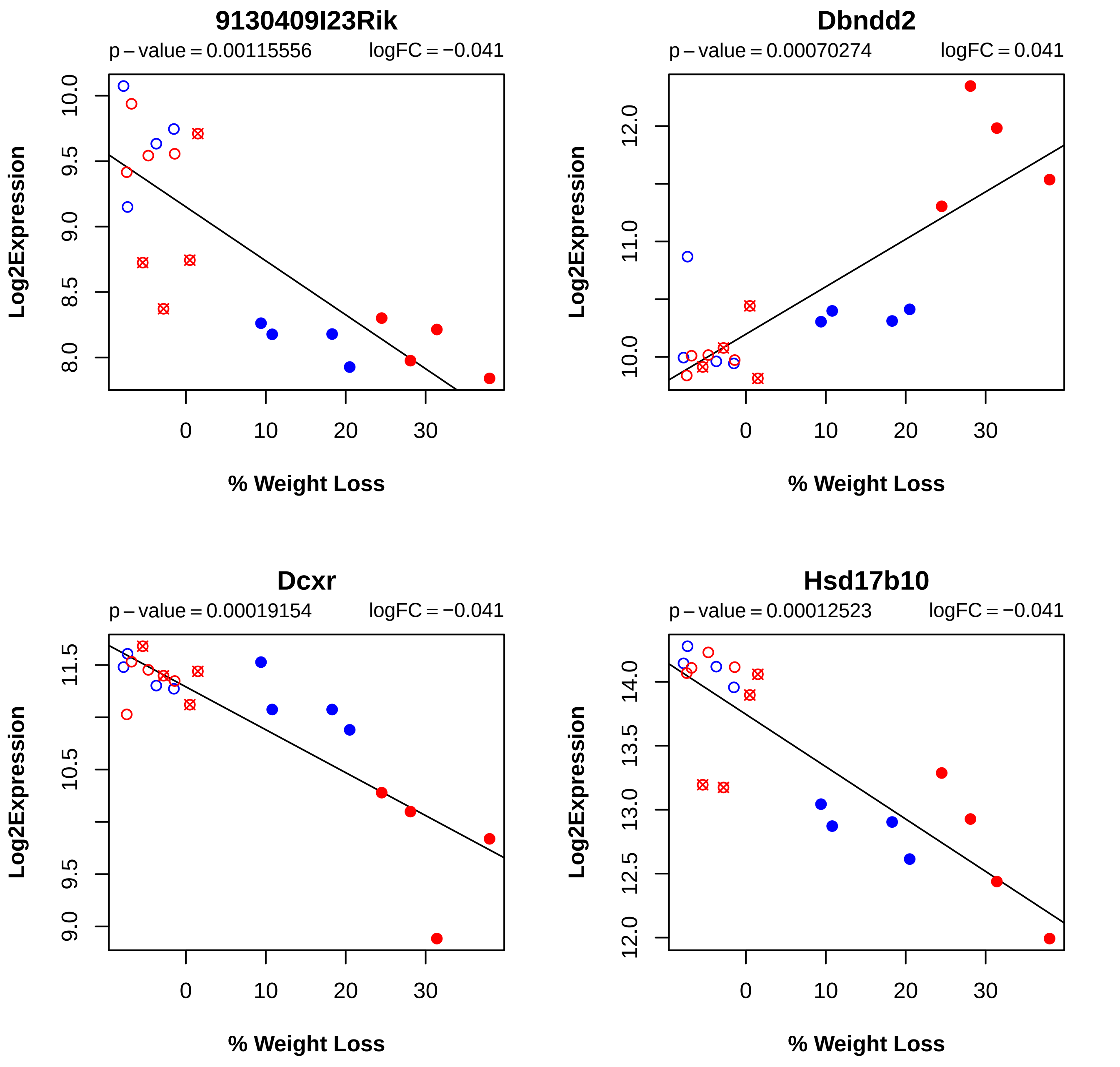
9130409I23Rik
p – value = 0.00115556
logFC = −0.041
Dbndd2
p – value = 0.00070274
logFC = 0.041
Dcxr
p – value = 0.00019154
logFC = −0.041
Hsd17b10
p – value = 0.00012523
logFC = −0.041
Log2Expression
% Weight Loss

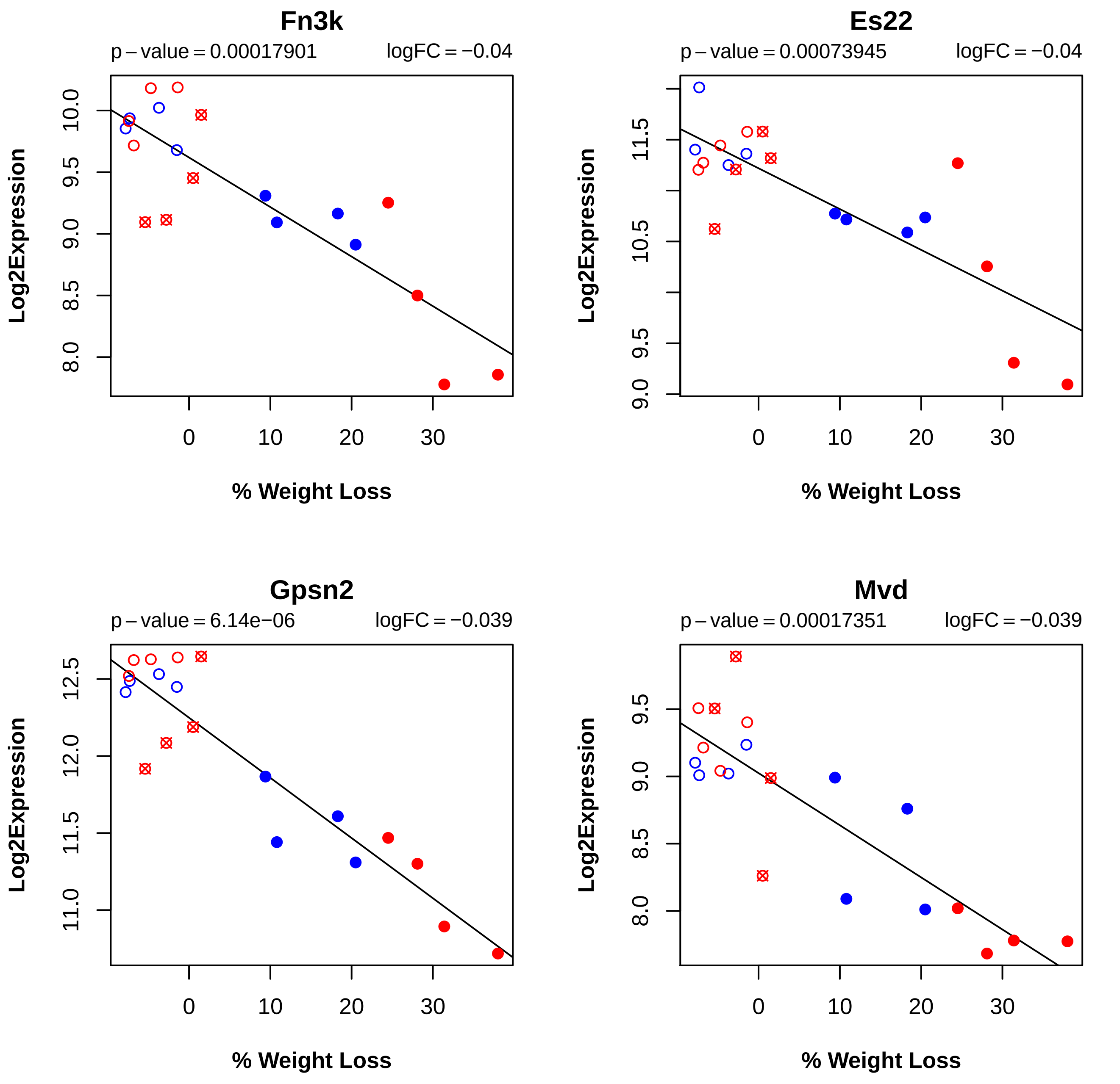
Fn3k
p – value = 0.00017901
logFC = −0.04
Log2Expression
% Weight Loss
Es22
p – value = 0.00073945
logFC = −0.04
Log2Expression
% Weight Loss
Gpsn2
p – value = 6.14e−06
logFC = −0.039
Log2Expression
% Weight Loss
Mvd
p – value = 0.00017351
logFC = −0.039
Log2Expression
% Weight Loss

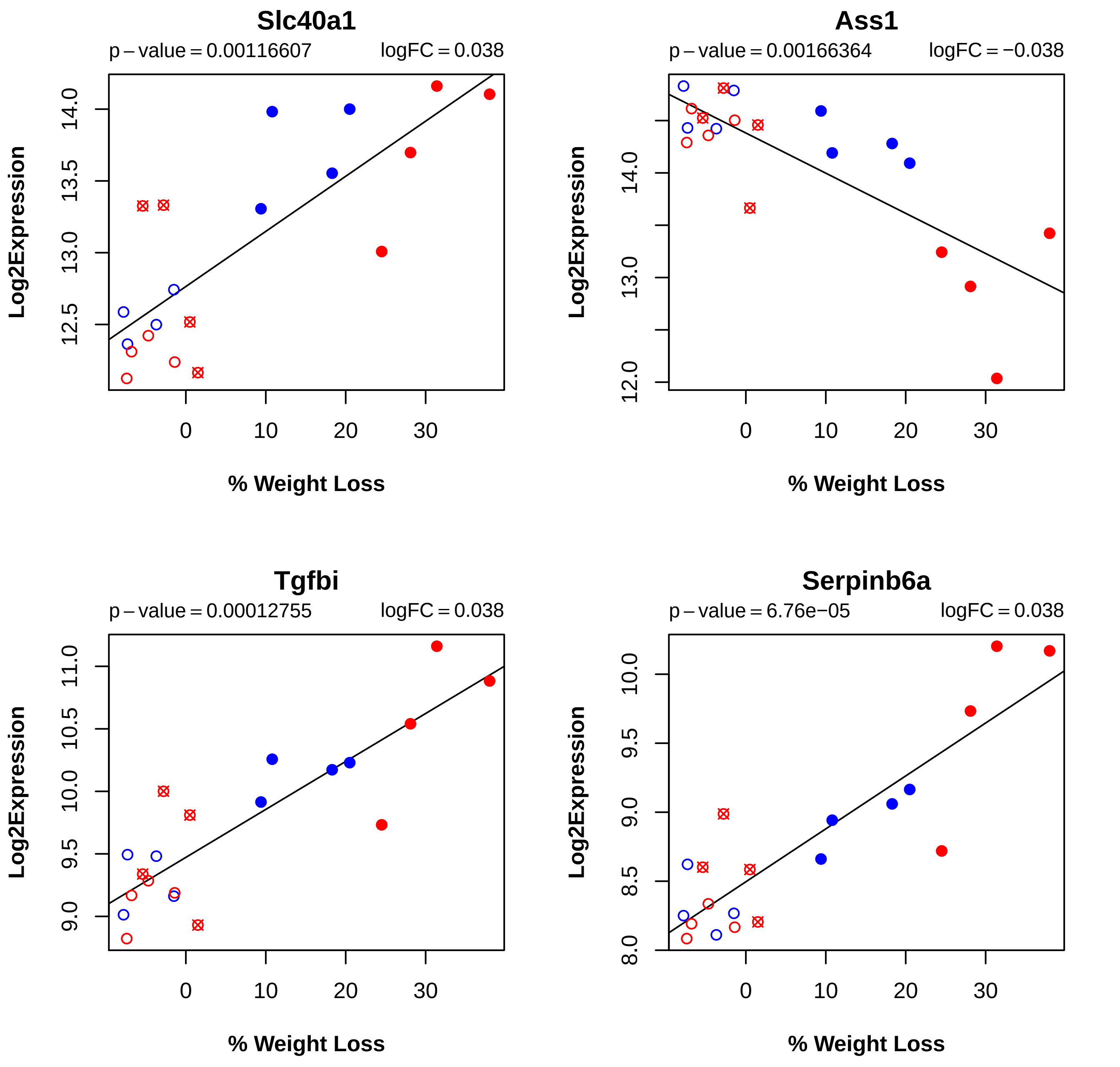

Slc40a1
p − value = 0.00116607
logFC = 0.038
Log2Expression
% Weight Loss
Ass1
p − value = 0.00166364
logFC = −0.038
Log2Expression
% Weight Loss
Tgfbi
p − value = 0.00012755
logFC = 0.038
Log2Expression
% Weight Loss
Serpinb6a
p − value = 6.76e−05
logFC = 0.038
Log2Expression
% Weight Loss

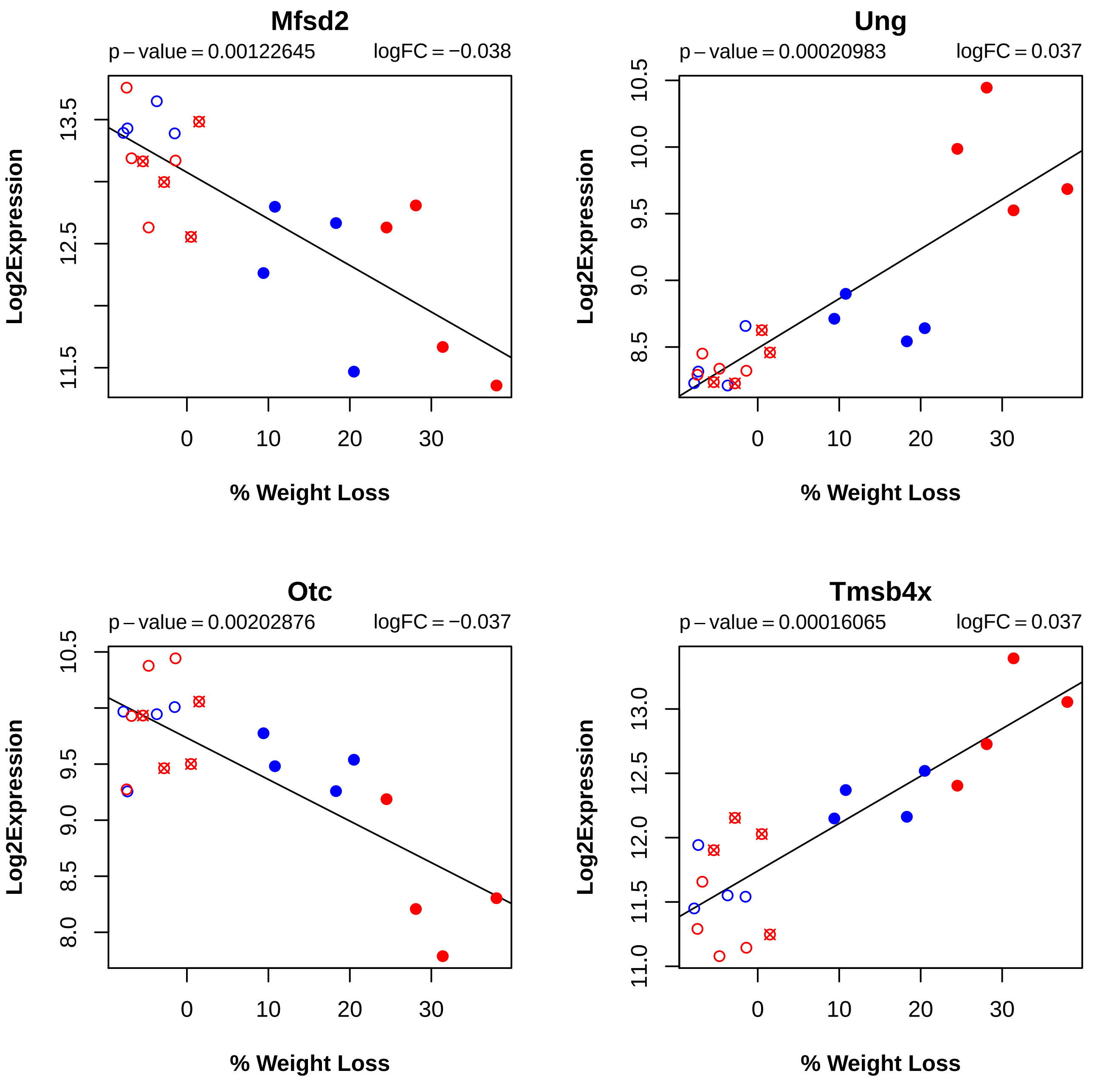

Mfsd2
p – value = 0.00122645
logFC = −0.038
Log2Expression
% Weight Loss
13.5
12.5
11.5
0
10
20
30
Ung
p – value = 0.00020983
logFC = 0.037
Log2Expression
% Weight Loss
10.5
10.0
9.5
9.0
8.5
0
10
20
30
Otc
p – value = 0.00202876
logFC = −0.037
Log2Expression
% Weight Loss
10.5
9.5
9.0
8.5
8.0
0
10
20
30
Tmsb4x
p – value = 0.00016065
logFC = 0.037
Log2Expression
% Weight Loss
13.0
12.5
12.0
11.5
11.0
0
10
20
30

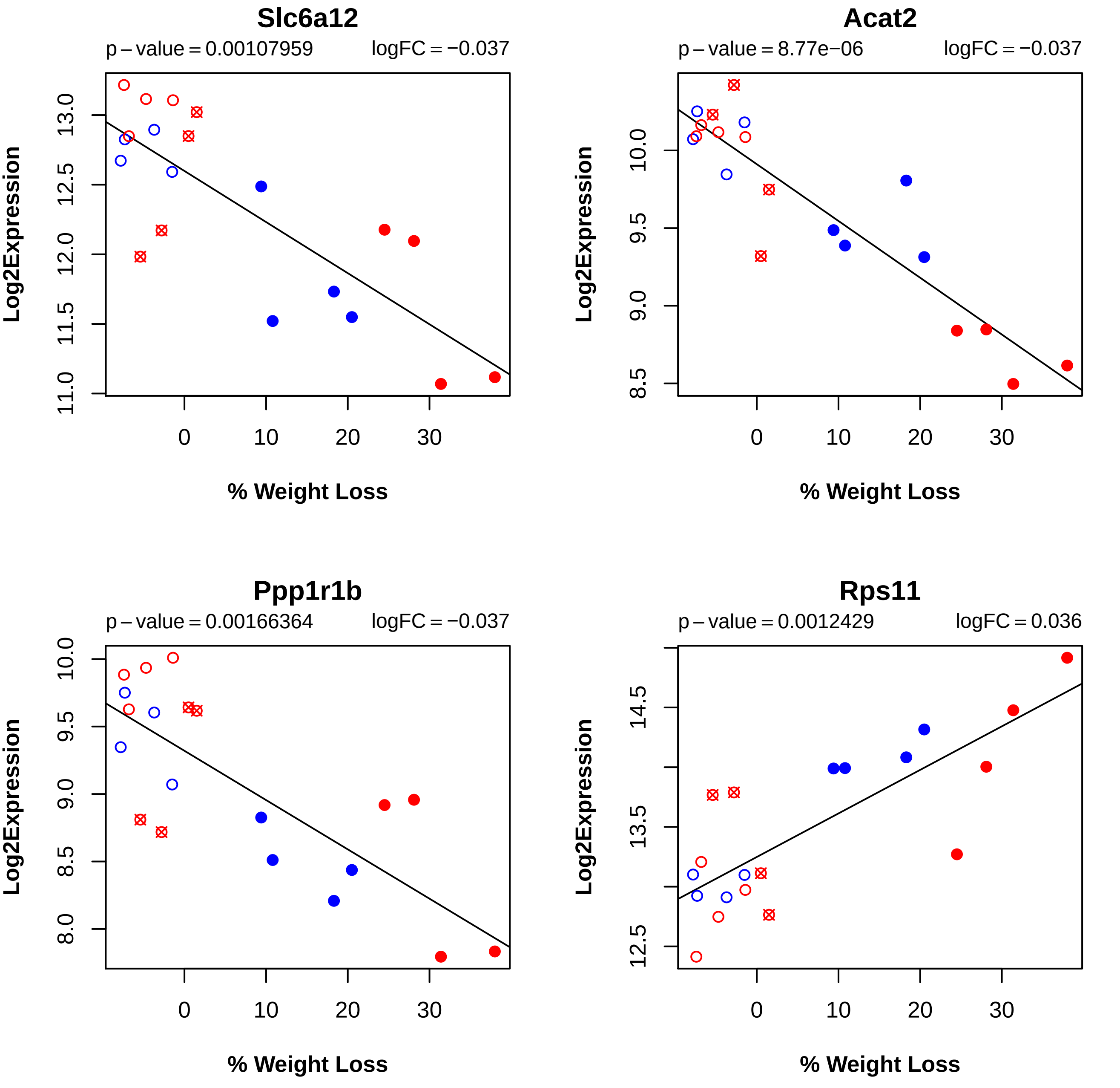
Slc6a12
p − value = 0.00107959
logFC = −0.037
Log2Expression
% Weight Loss
Acat2
p − value = 8.77e−06
logFC = −0.037
Log2Expression
% Weight Loss
Ppp1r1b
p − value = 0.00166364
logFC = −0.037
Log2Expression
% Weight Loss
Rps11
p − value = 0.0012429
logFC = 0.036
Log2Expression
% Weight Loss

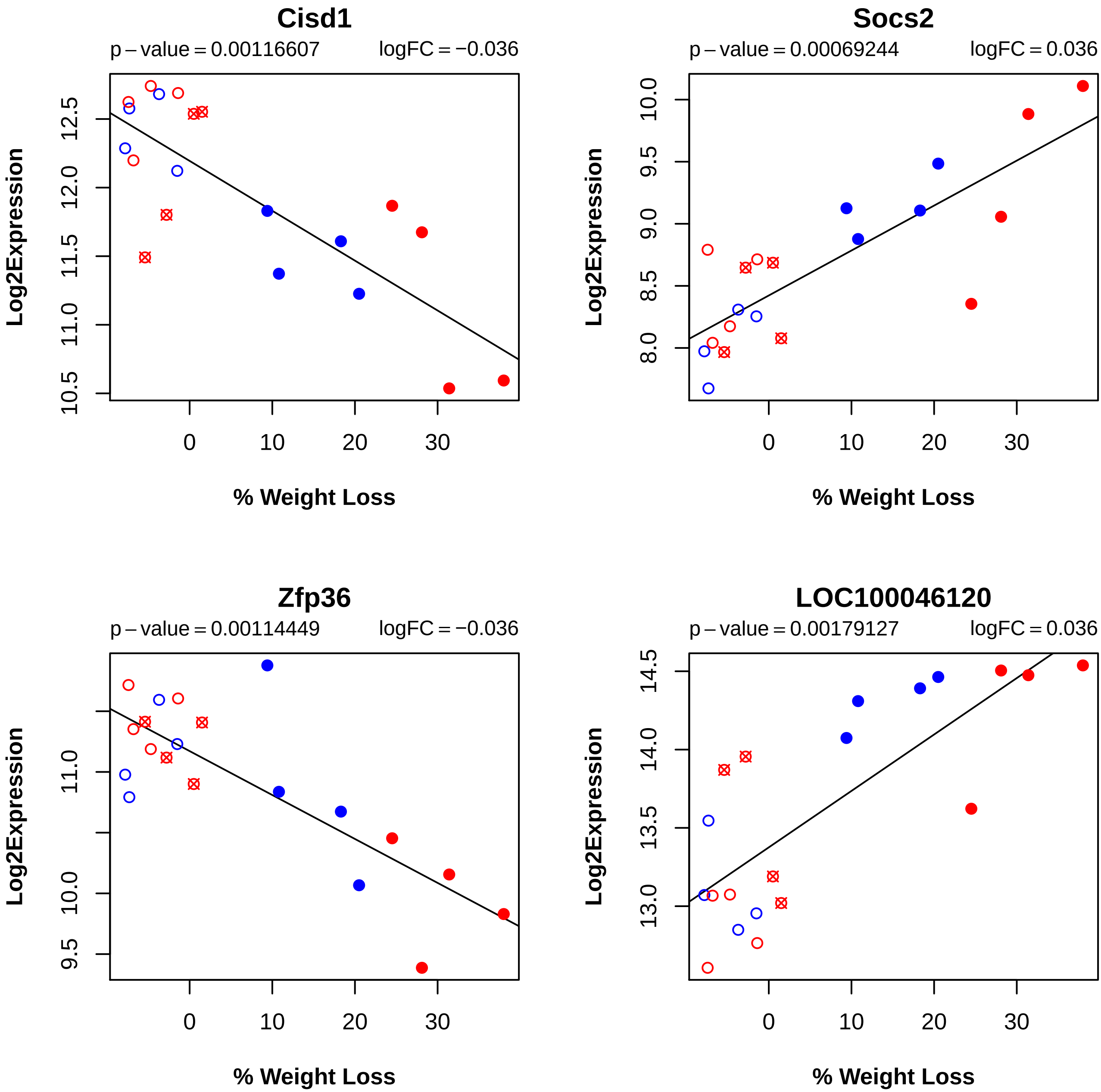

Cisd1
p – value = 0.00116607
logFC = −0.036
Log2Expression
% Weight Loss
Socs2
p – value = 0.00069244
logFC = 0.036
Log2Expression
% Weight Loss
Zfp36
p – value = 0.00114449
logFC = −0.036
Log2Expression
% Weight Loss
LOC100046120
p – value = 0.00179127
logFC = 0.036
Log2Expression
% Weight Loss

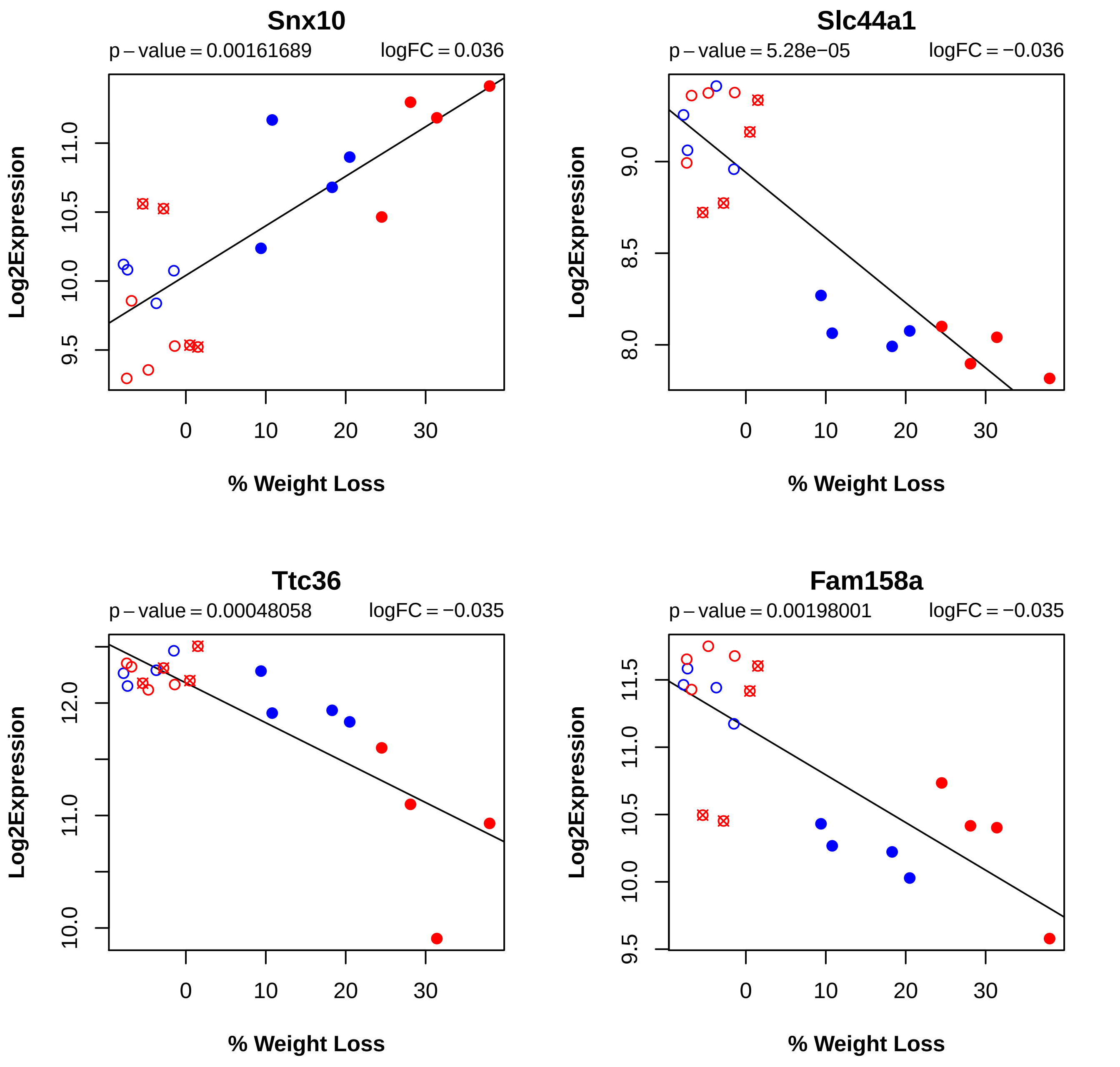

Snx10
p – value = 0.00161689
logFC = 0.036
Log2Expression
9.5
10.0
10.5
11.0
0
10
20
30
% Weight Loss
Slc44a1
p – value = 5.28e−05
logFC = −0.036
Log2Expression
8.0
8.5
9.0
0
10
20
30
% Weight Loss
Ttc36
p – value = 0.00048058
logFC = −0.035
Log2Expression
10.0
11.0
12.0
0
10
20
30
% Weight Loss
Fam158a
p – value = 0.00198001
logFC = −0.035
Log2Expression
9.5
10.0
10.5
11.0
11.5
0
10
20
30
% Weight Loss

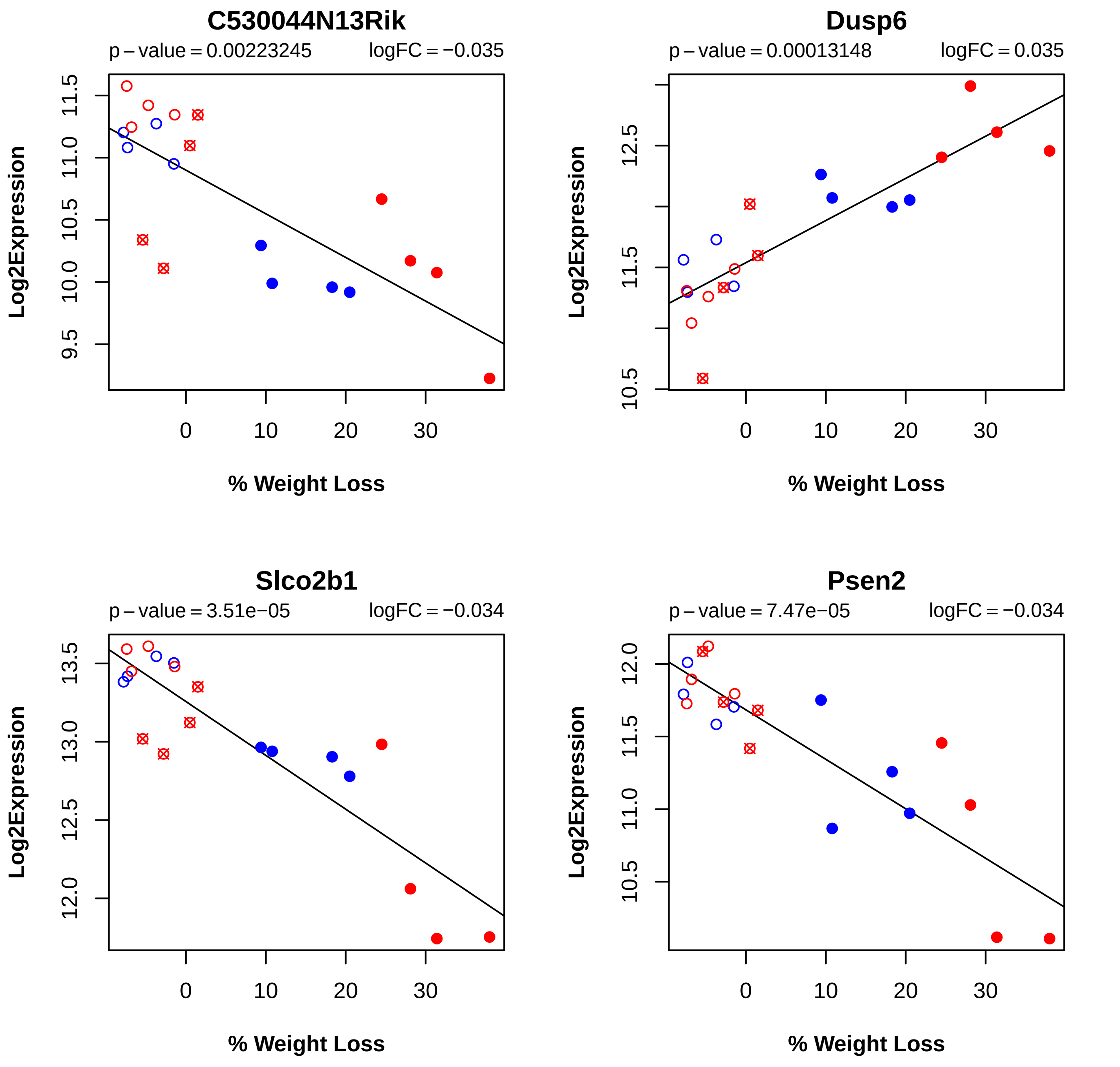
C530044N13Rik
p − value = 0.00223245
logFC = −0.035
Log2Expression
% Weight Loss
Dusp6
p − value = 0.00013148
logFC = 0.035
Log2Expression
% Weight Loss
Slco2b1
p − value = 3.51e−05
logFC = −0.034
Log2Expression
% Weight Loss
Psen2
p − value = 7.47e−05
logFC = −0.034
Log2Expression
% Weight Loss

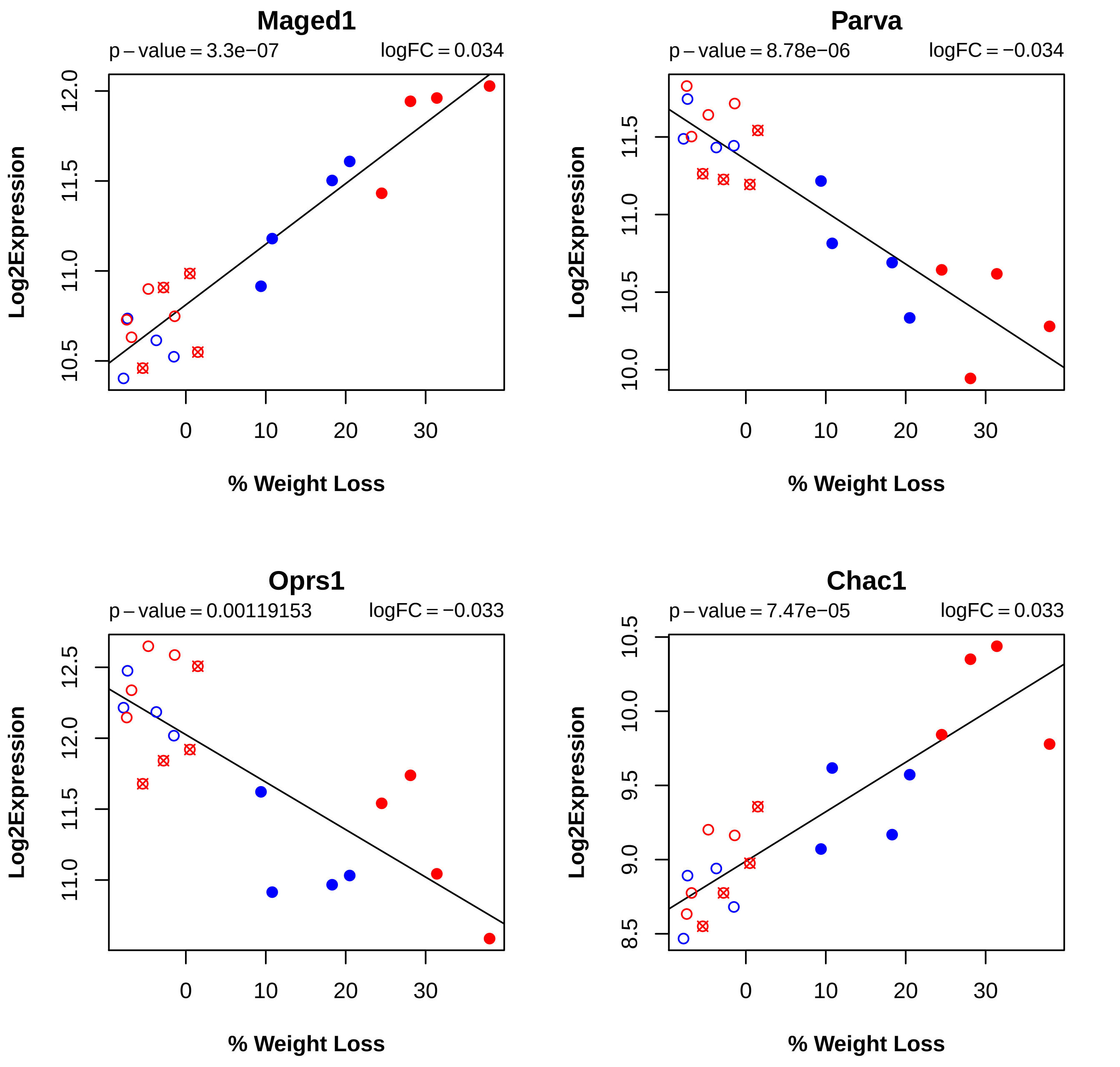
Maged1
p – value = 3.3e−07
logFC = 0.034
Log2Expression
% Weight Loss
Parva
p – value = 8.78e−06
logFC = −0.034
Log2Expression
% Weight Loss
Oprs1
p – value = 0.00119153
logFC = −0.033
Log2Expression
% Weight Loss
Chac1
p – value = 7.47e−05
logFC = 0.033
Log2Expression
% Weight Loss

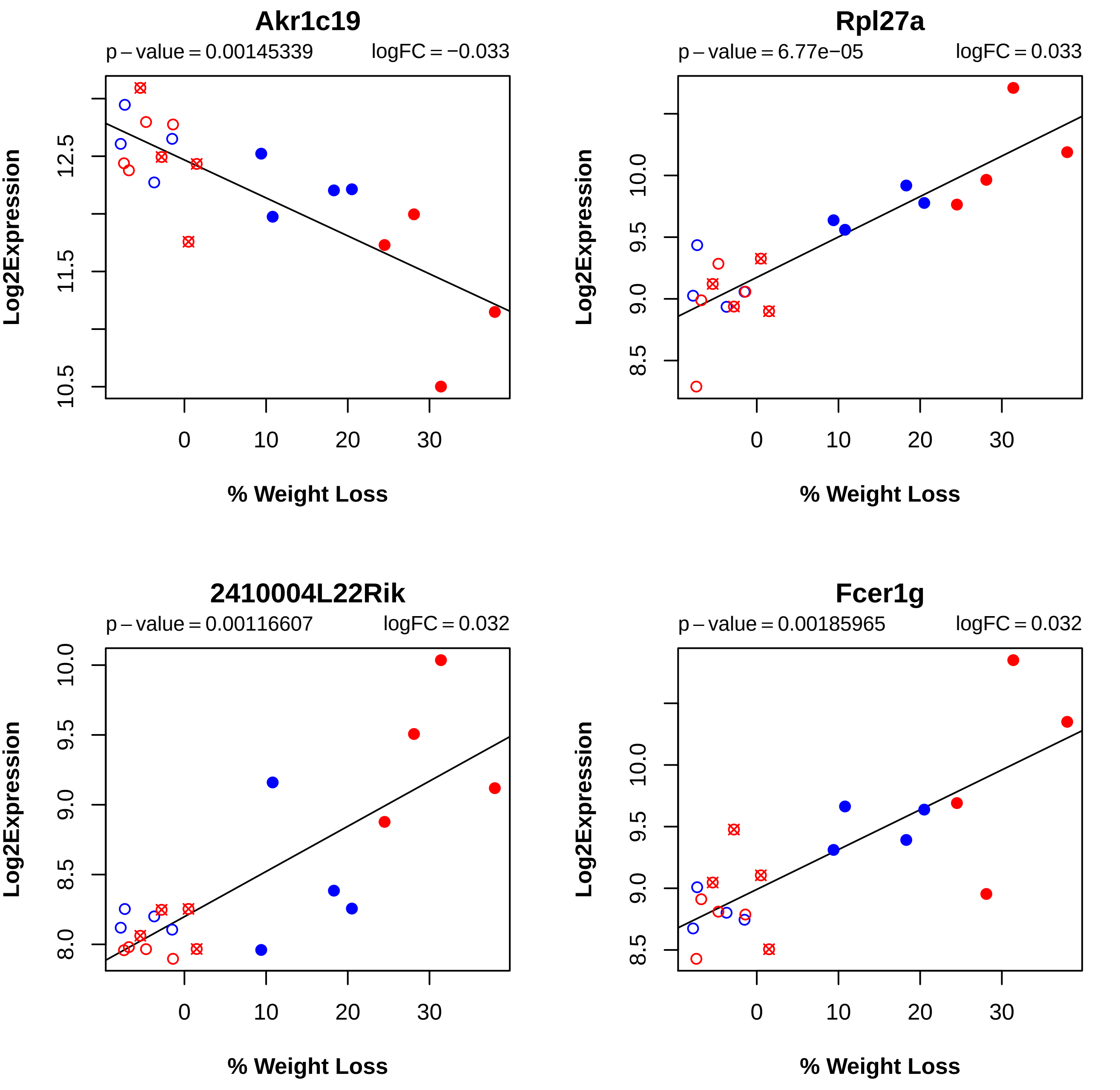

Akr1c19
p − value = 0.00145339
logFC = −0.033
Log2Expression
% Weight Loss
Rpl27a
p − value = 6.77e−05
logFC = 0.033
Log2Expression
% Weight Loss
2410004L22Rik
p − value = 0.00116607
logFC = 0.032
Log2Expression
% Weight Loss
Fcer1g
p − value = 0.00185965
logFC = 0.032
Log2Expression
% Weight Loss

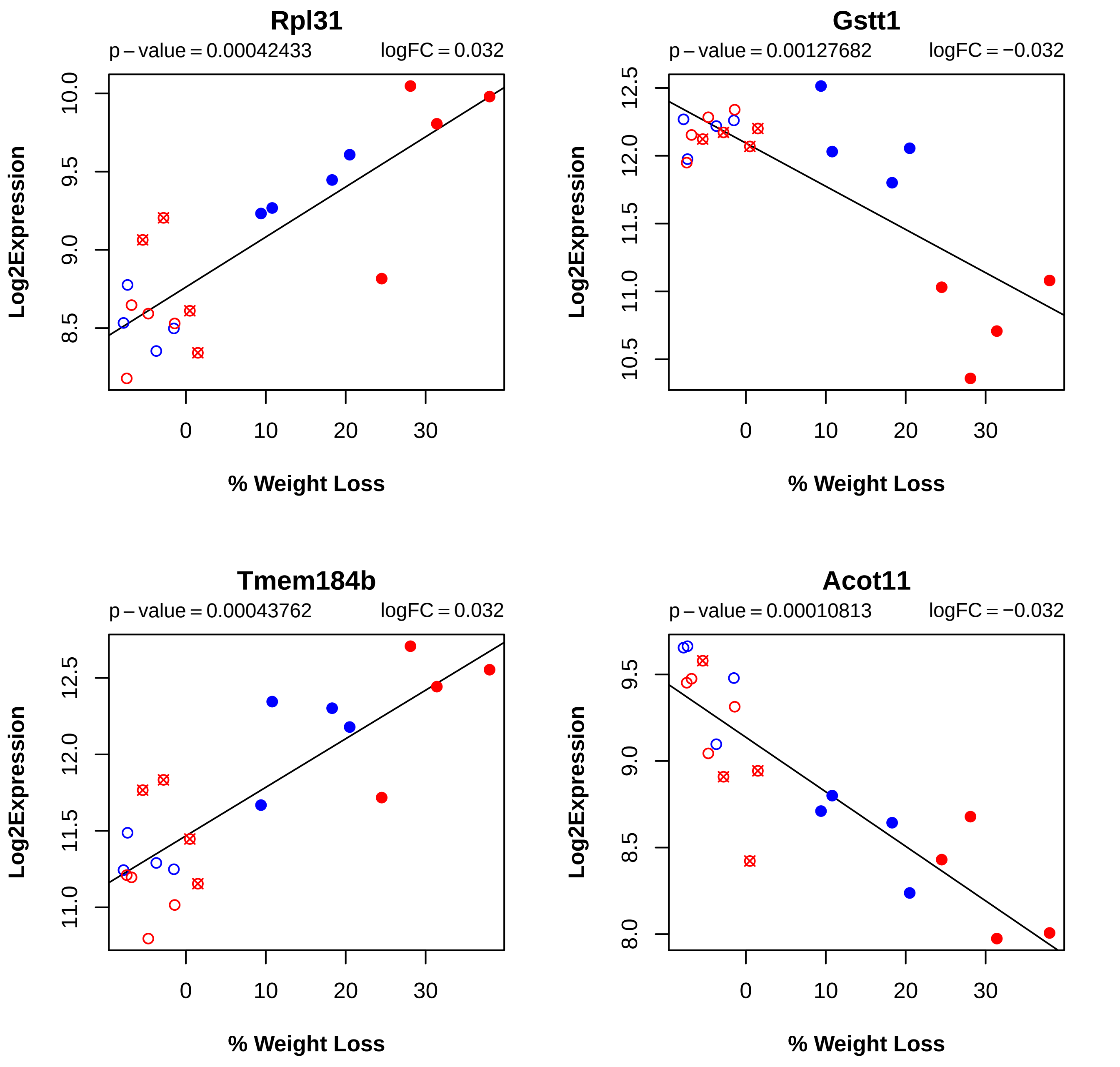

Rpl31
p – value = 0.00042433
logFC = 0.032
Log2Expression
% Weight Loss
Gstt1
p – value = 0.00127682
logFC = −0.032
Log2Expression
% Weight Loss
Tmem184b
p – value = 0.00043762
logFC = 0.032
Log2Expression
% Weight Loss
Acot11
p – value = 0.00010813
logFC = −0.032
Log2Expression
% Weight Loss

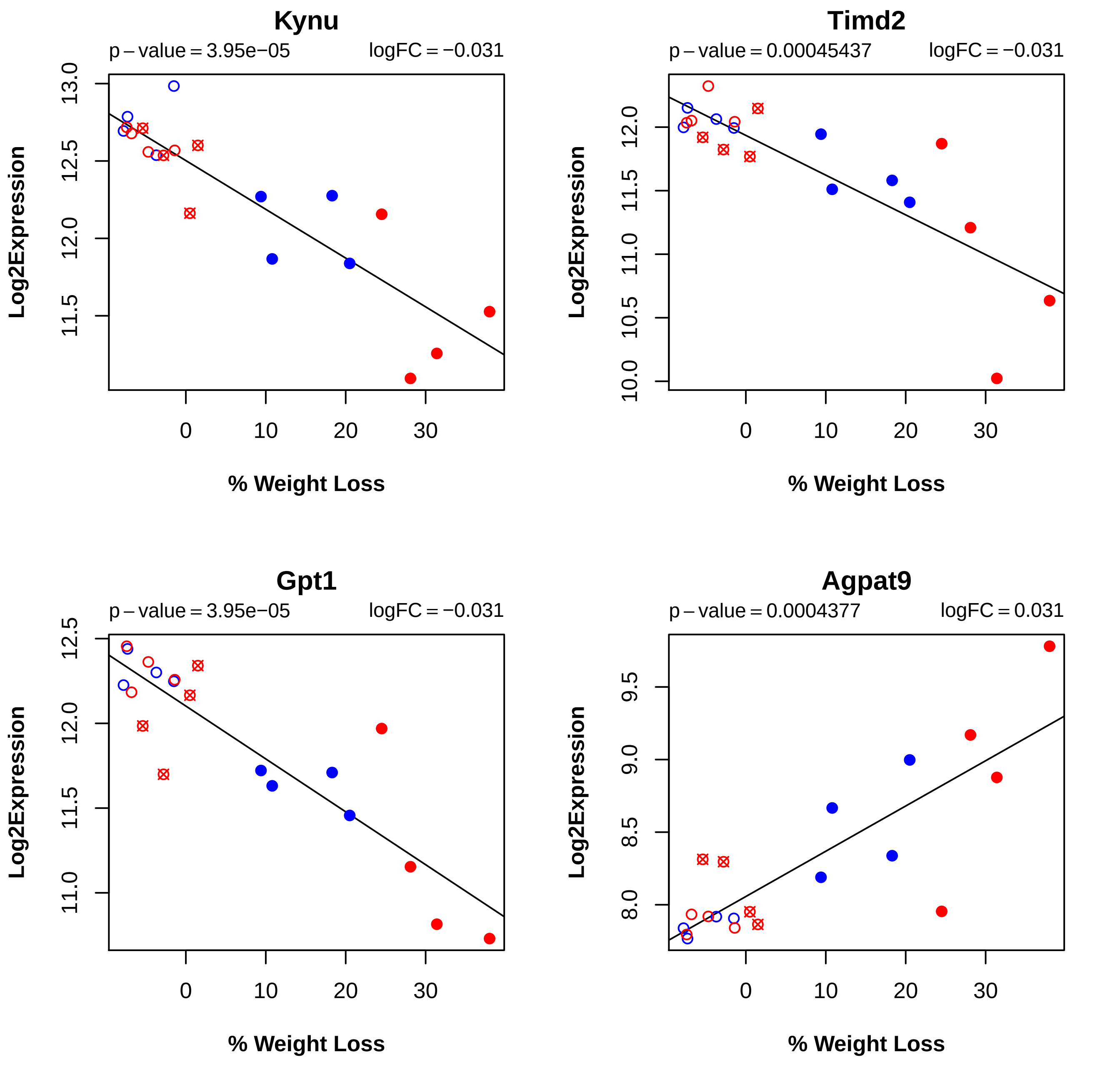

Kynu
p − value = 3.95e−05
logFC = −0.031
Log2Expression
% Weight Loss
Timd2
p − value = 0.00045437
logFC = −0.031
Log2Expression
% Weight Loss
Gpt1
p − value = 3.95e−05
logFC = −0.031
Log2Expression
% Weight Loss
Agpat9
p − value = 0.0004377
logFC = 0.031
Log2Expression
% Weight Loss

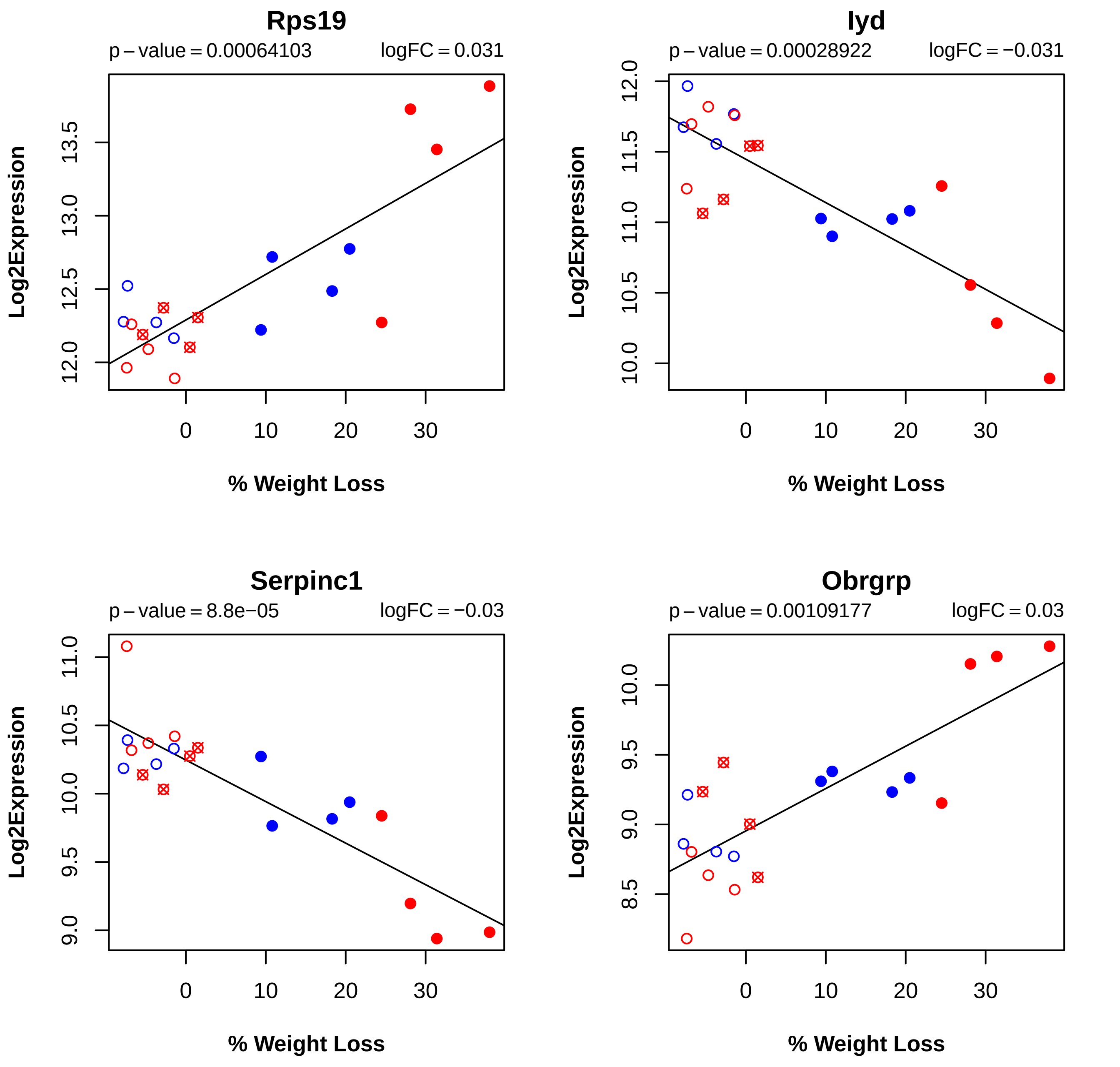
Rps19
p − value = 0.00064103
logFC = 0.031
Log2Expression
12.0
12.5
13.0
13.5
0
10
20
30
% Weight Loss
Iyd
p − value = 0.00028922
logFC = −0.031
Log2Expression
10.0
10.5
11.0
11.5
12.0
0
10
20
30
% Weight Loss
Serpinc1
p − value = 8.8e−05
logFC = −0.03
Log2Expression
9.0
9.5
10.0
10.5
11.0
0
10
20
30
% Weight Loss
Obrgrp
p − value = 0.00109177
logFC = 0.03
Log2Expression
8.5
9.0
9.5
10.0
0
10
20
30
% Weight Loss

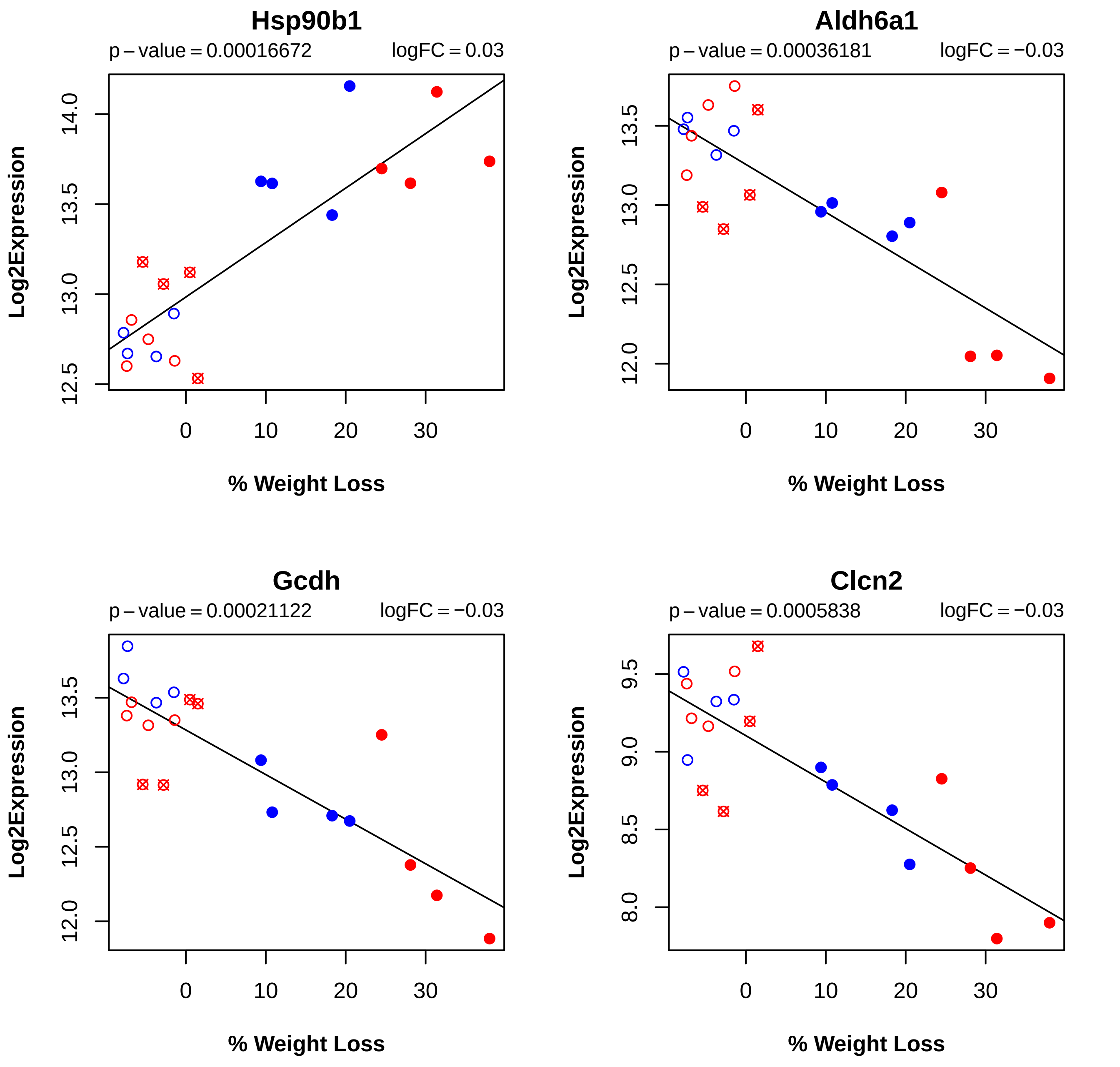

Hsp90b1
p − value = 0.00016672
logFC = 0.03
Log2Expression
% Weight Loss
Aldh6a1
p − value = 0.00036181
logFC = −0.03
Log2Expression
% Weight Loss
Gcdh
p − value = 0.00021122
logFC = −0.03
Log2Expression
% Weight Loss
Clcn2
p − value = 0.0005838
logFC = −0.03
Log2Expression
% Weight Loss

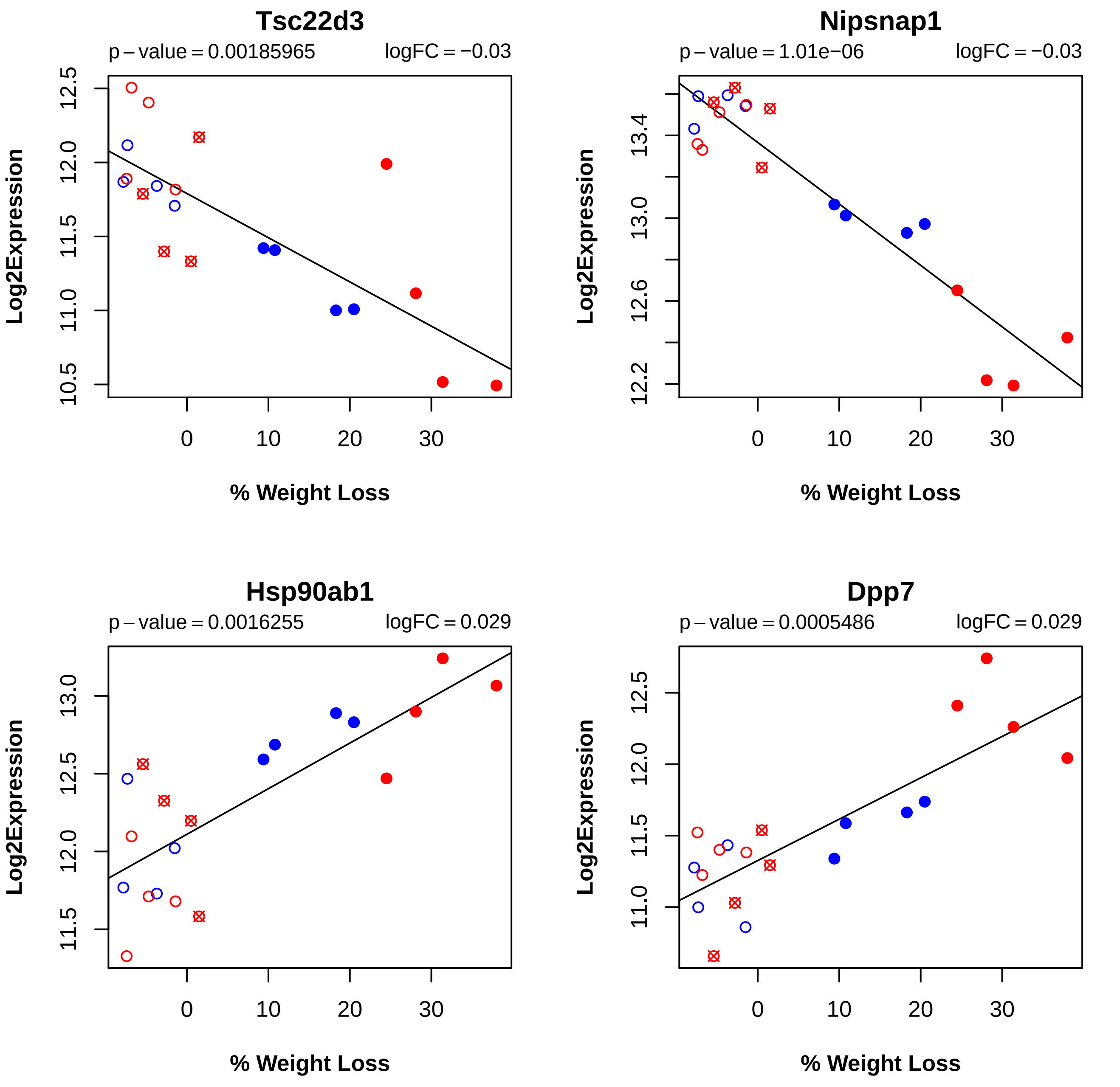
Tsc22d3
p − value = 0.00185965
logFC = −0.03
Log2Expression
% Weight Loss
Nipsnap1
p − value = 1.01e−06
logFC = −0.03
Log2Expression
% Weight Loss
Hsp90ab1
p − value = 0.0016255
logFC = 0.029
Log2Expression
% Weight Loss
Dpp7
p − value = 0.0005486
logFC = 0.029
Log2Expression
% Weight Loss

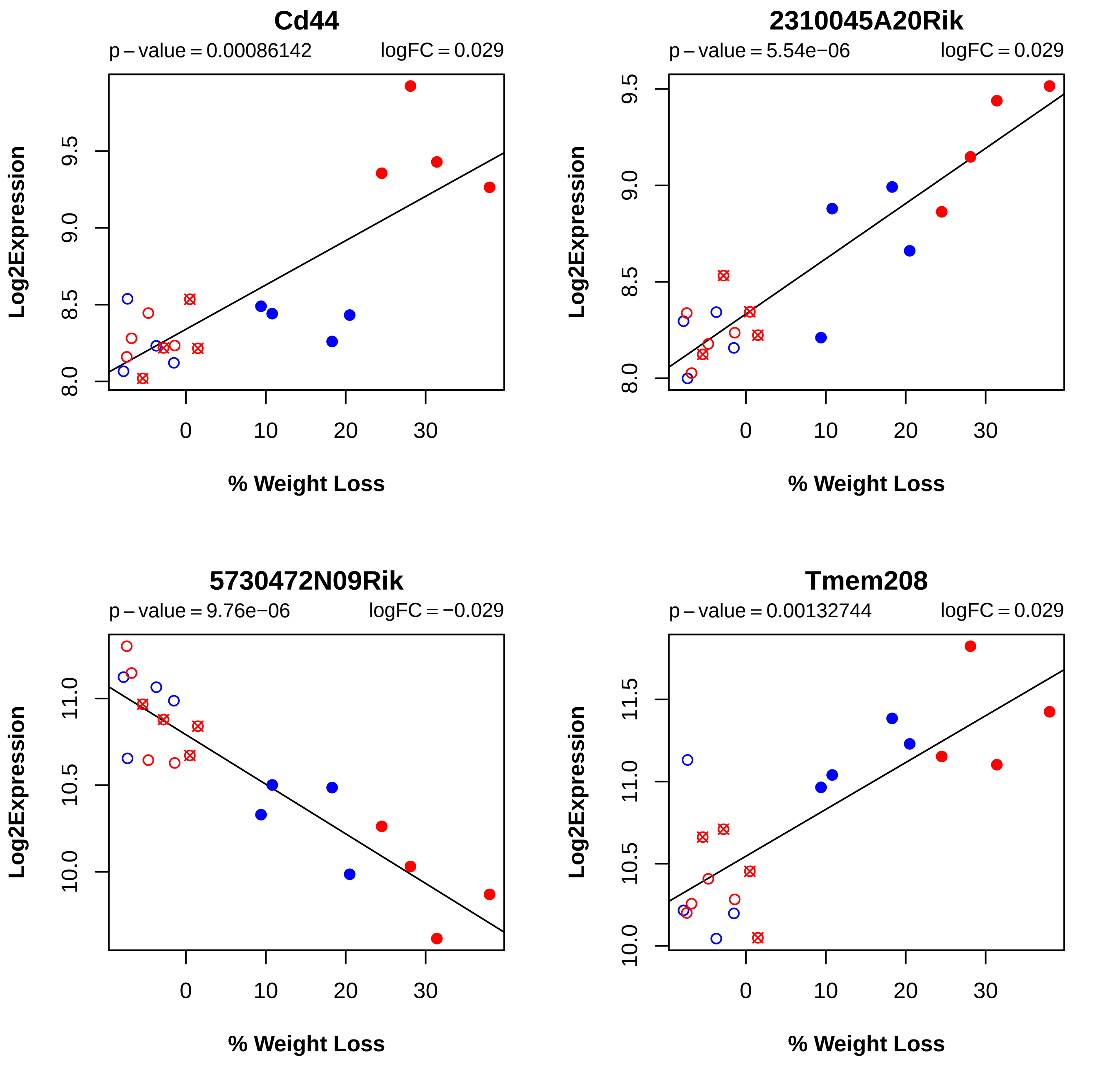

Cd44
p − value = 0.00086142
logFC = 0.029
2310045A20Rik
p − value = 5.54e−06
logFC = 0.029
5730472N09Rik
p − value = 9.76e−06
logFC = −0.029
Tmem208
p − value = 0.00132744
logFC = 0.029
Log2Expression
% Weight Loss
8.0
8.5
9.0
9.5
10.0
10.5
11.0
11.5
0
10
20
30

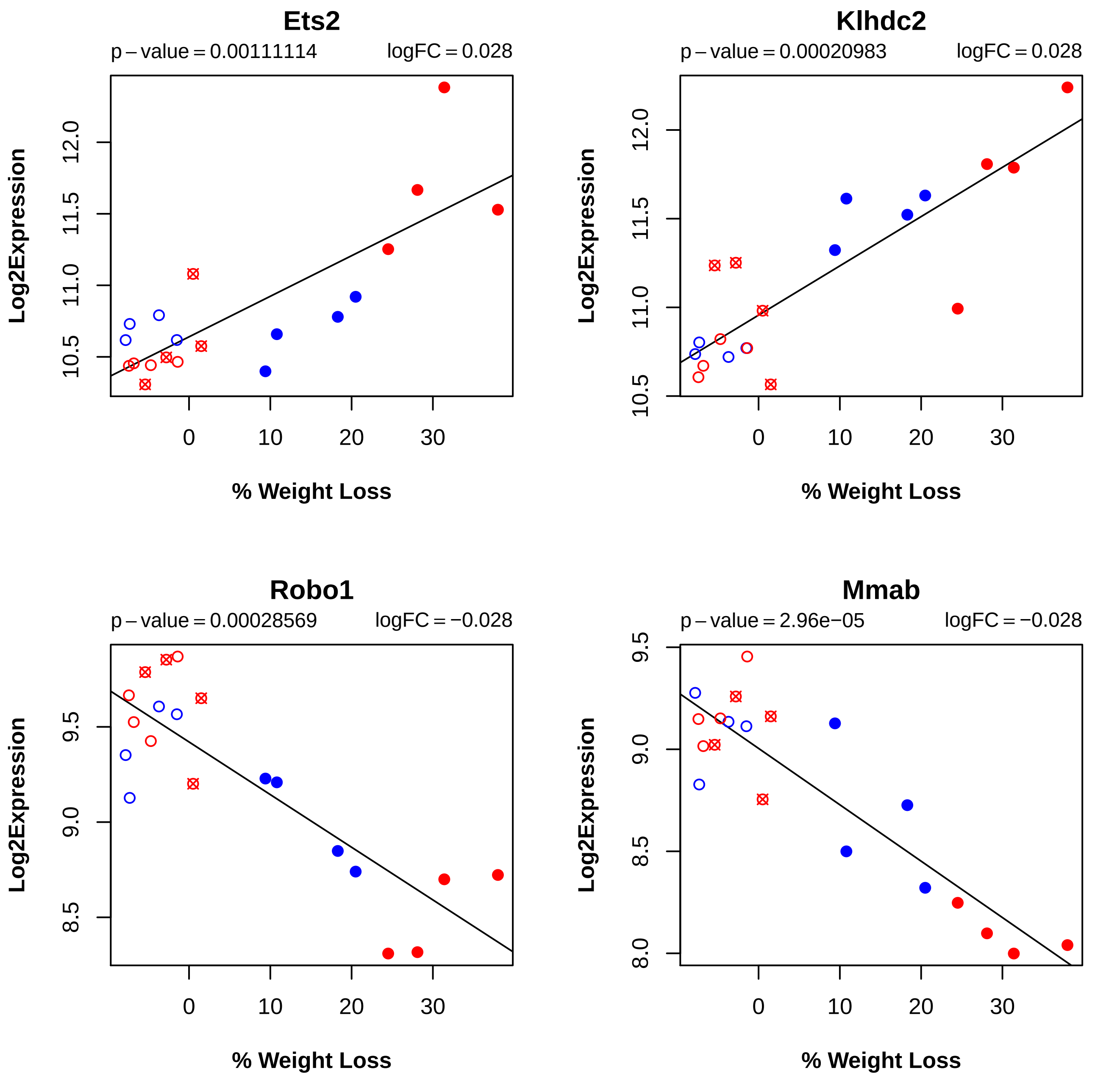

Ets2
p − value = 0.00111114
logFC = 0.028
Log2Expression
% Weight Loss
Klhdc2
p − value = 0.00020983
logFC = 0.028
Log2Expression
% Weight Loss
Robo1
p − value = 0.00028569
logFC = −0.028
Log2Expression
% Weight Loss
Mmab
p − value = 2.96e−05
logFC = −0.028
Log2Expression
% Weight Loss

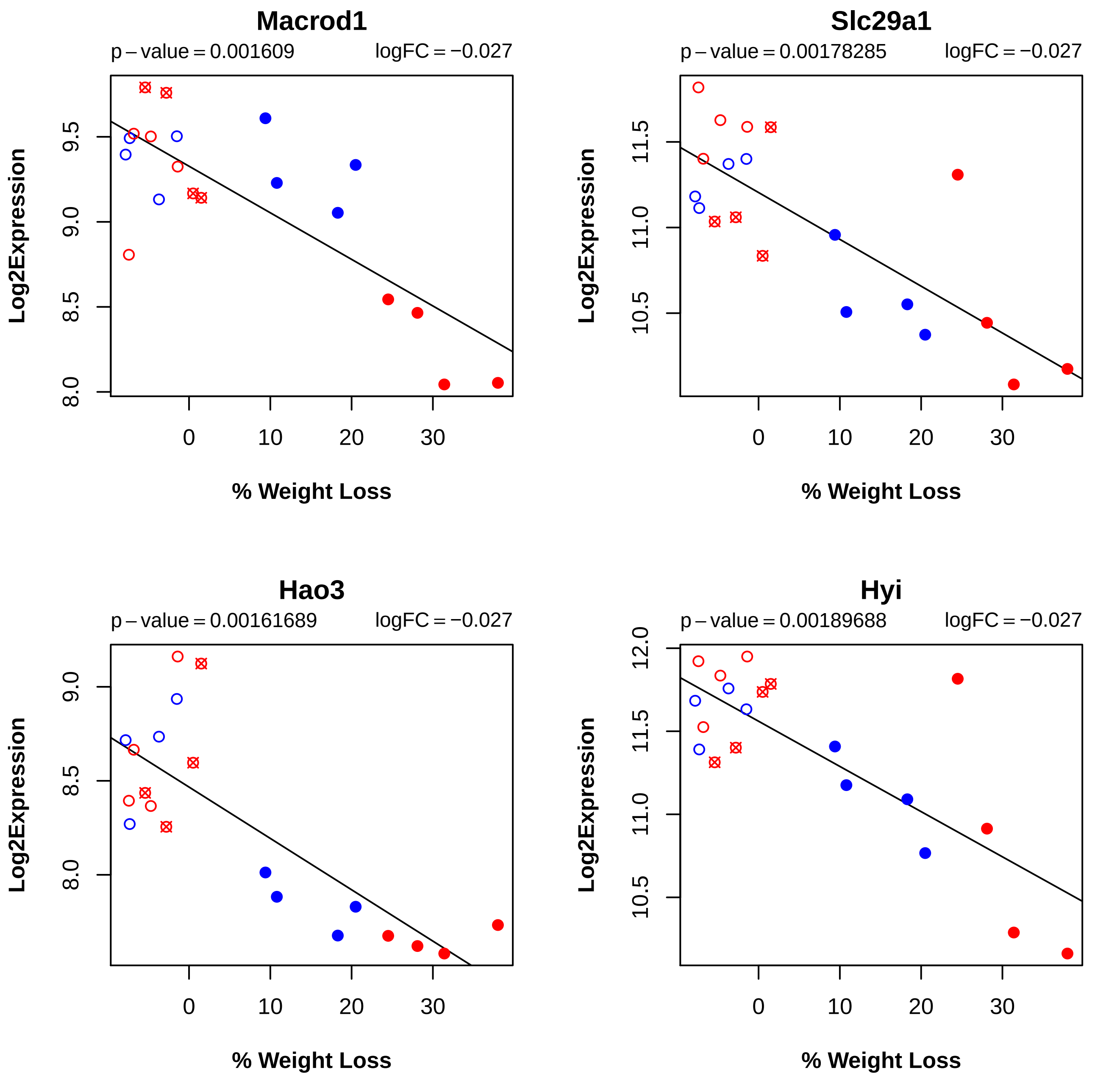
Macrod1
p − value = 0.001609
logFC = −0.027
Slc29a1
p − value = 0.00178285
logFC = −0.027
Hao3
p − value = 0.00161689
logFC = −0.027
Hyi
p − value = 0.00189688
logFC = −0.027
Log2Expression
% Weight Loss

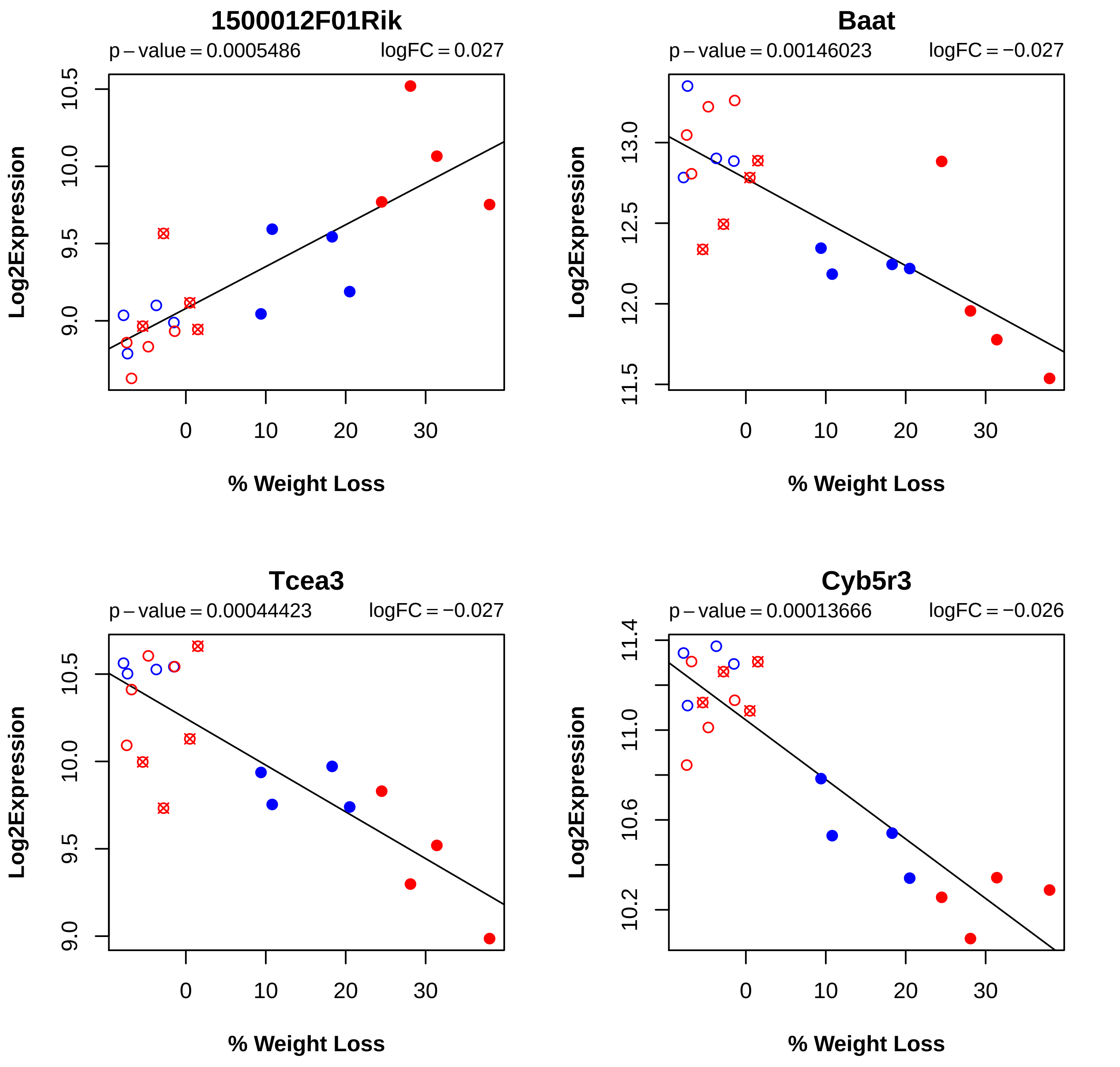
1500012F01Rik
p − value = 0.0005486
logFC = 0.027
Log2Expression
% Weight Loss
Baat
p − value = 0.00146023
logFC = −0.027
Log2Expression
% Weight Loss
Tcea3
p − value = 0.00044423
logFC = −0.027
Log2Expression
% Weight Loss
Cyb5r3
p − value = 0.00013666
logFC = −0.026
Log2Expression
% Weight Loss

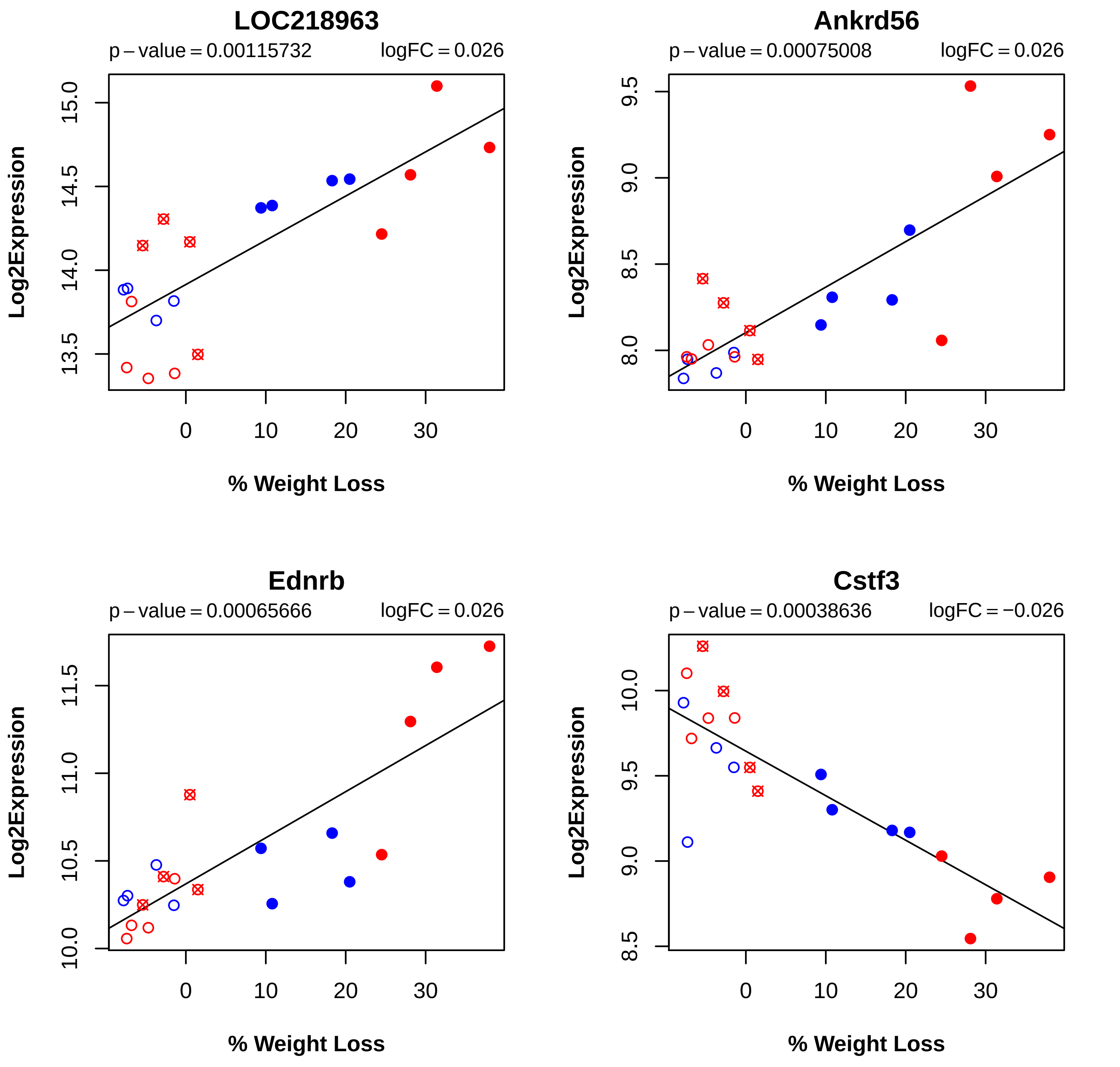
LOC218963
p – value = 0.00115732
logFC = 0.026
Log2Expression
% Weight Loss
Ankrd56
p – value = 0.00075008
logFC = 0.026
Log2Expression
% Weight Loss
Ednrb
p – value = 0.00065666
logFC = 0.026
Log2Expression
% Weight Loss
Cstf3
p – value = 0.00038636
logFC = −0.026
Log2Expression
% Weight Loss

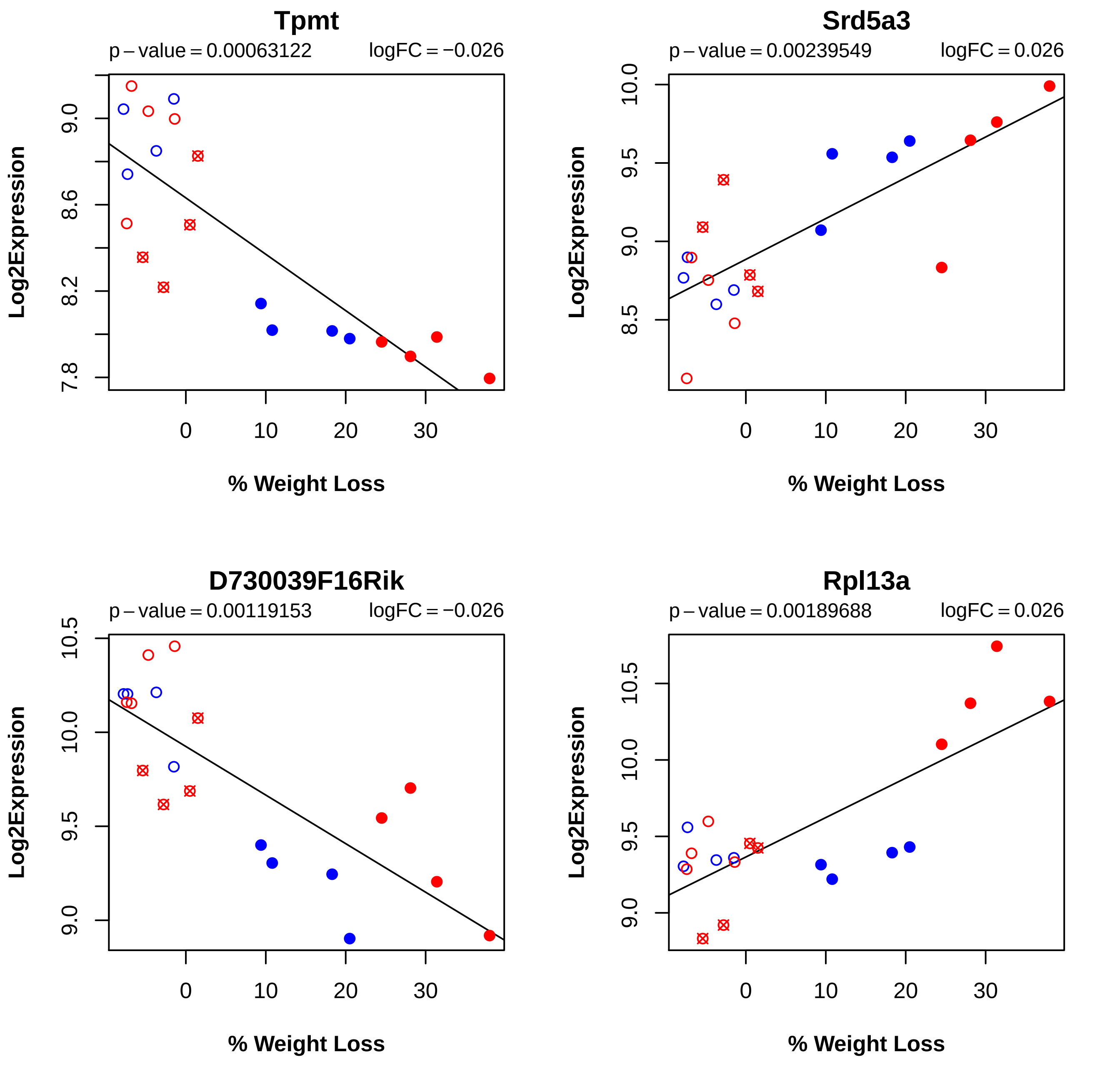

Tpmt
p – value = 0.00063122
logFC = −0.026
Srd5a3
p – value = 0.00239549
logFC = 0.026
D730039F16Rik
p – value = 0.00119153
logFC = −0.026
Rpl13a
p – value = 0.00189688
logFC = 0.026
Log2Expression
% Weight Loss

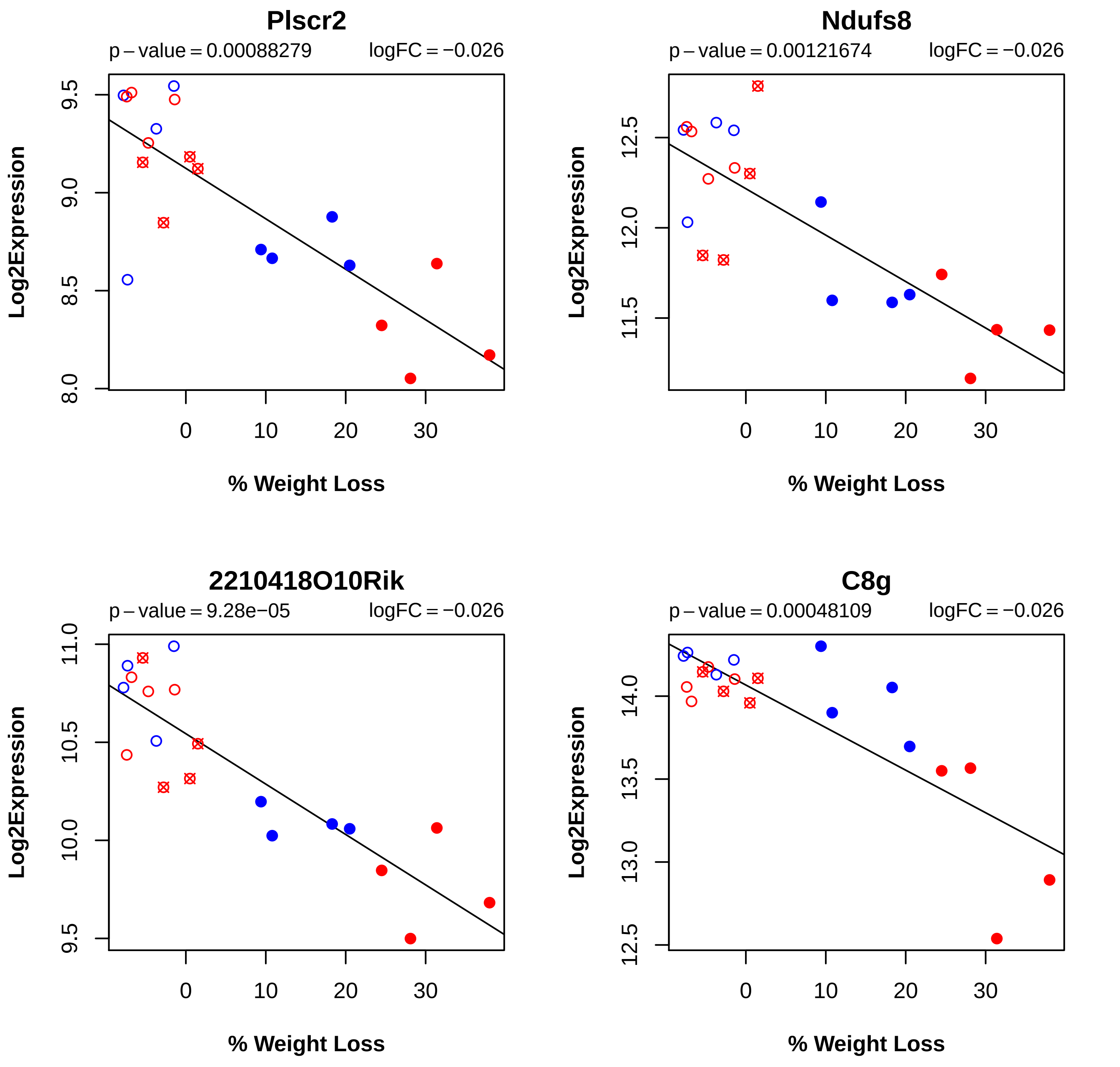
Plscr2
p − value = 0.00088279
logFC = −0.026
Log2Expression
% Weight Loss
Ndufs8
p − value = 0.00121674
logFC = −0.026
Log2Expression
% Weight Loss
2210418O10Rik
p − value = 9.28e−05
logFC = −0.026
Log2Expression
% Weight Loss
C8g
p − value = 0.00048109
logFC = −0.026
Log2Expression
% Weight Loss

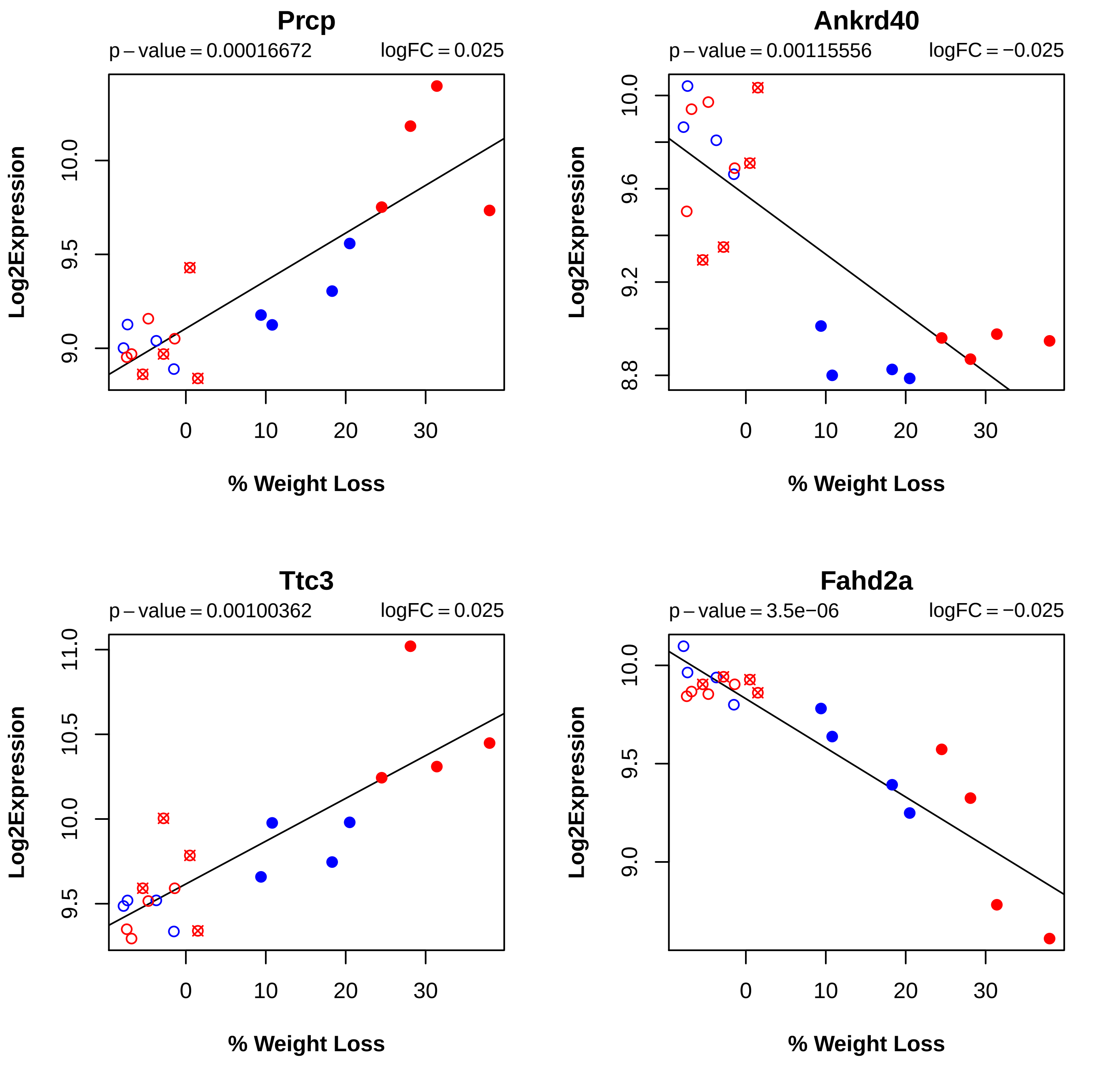

Prcp
p − value = 0.00016672
logFC = 0.025
Ankrd40
p − value = 0.00115556
logFC = −0.025
Ttc3
p − value = 0.00100362
logFC = 0.025
Fahd2a
p − value = 3.5e−06
logFC = −0.025
Log2Expression
% Weight Loss

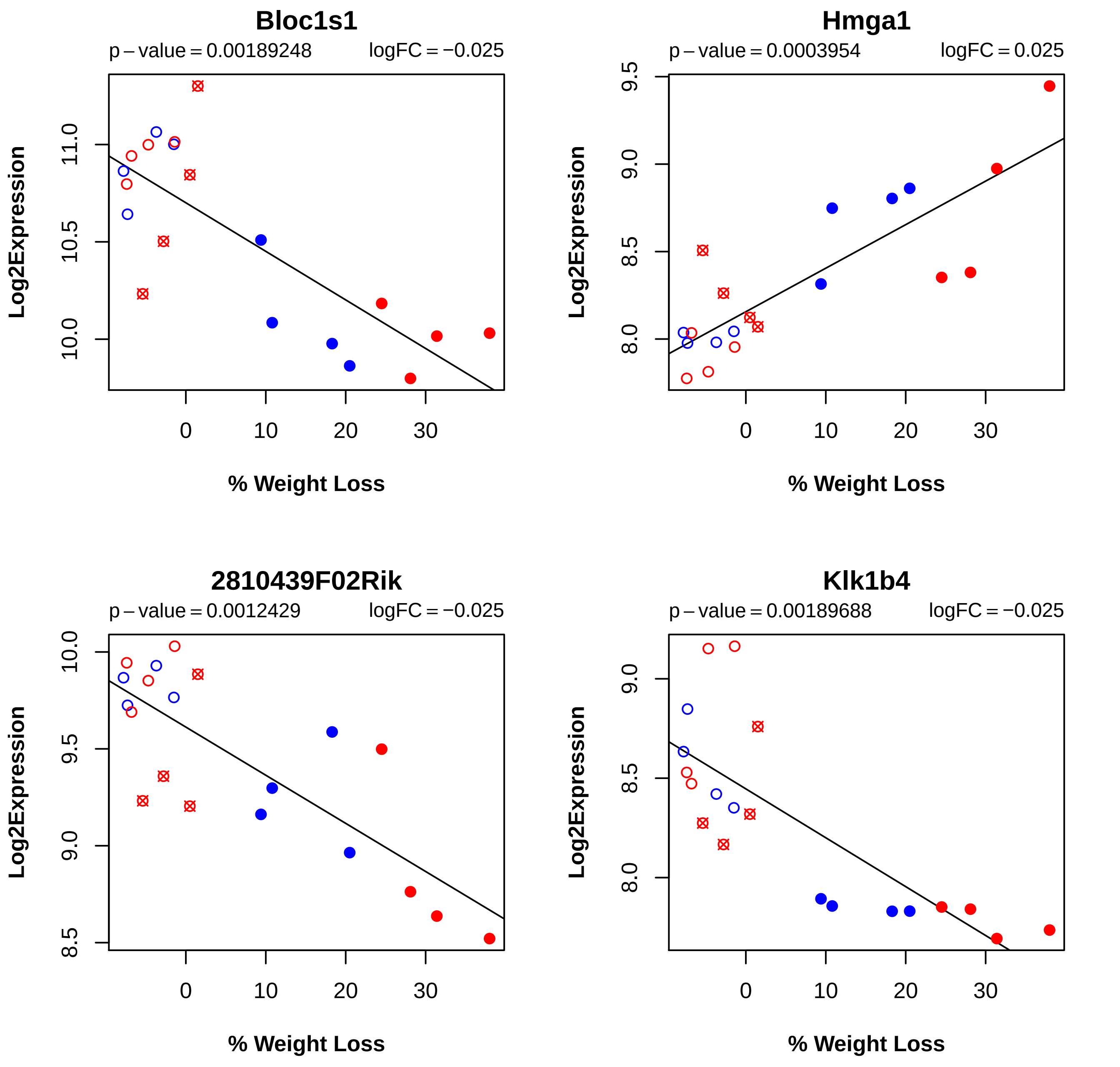

Bloc1s1
p – value = 0.00189248
logFC = −0.025
Log2Expression
% Weight Loss
Hmga1
p – value = 0.0003954
logFC = 0.025
Log2Expression
% Weight Loss
2810439F02Rik
p – value = 0.0012429
logFC = −0.025
Log2Expression
% Weight Loss
Klk1b4
p – value = 0.00189688
logFC = −0.025
Log2Expression
% Weight Loss

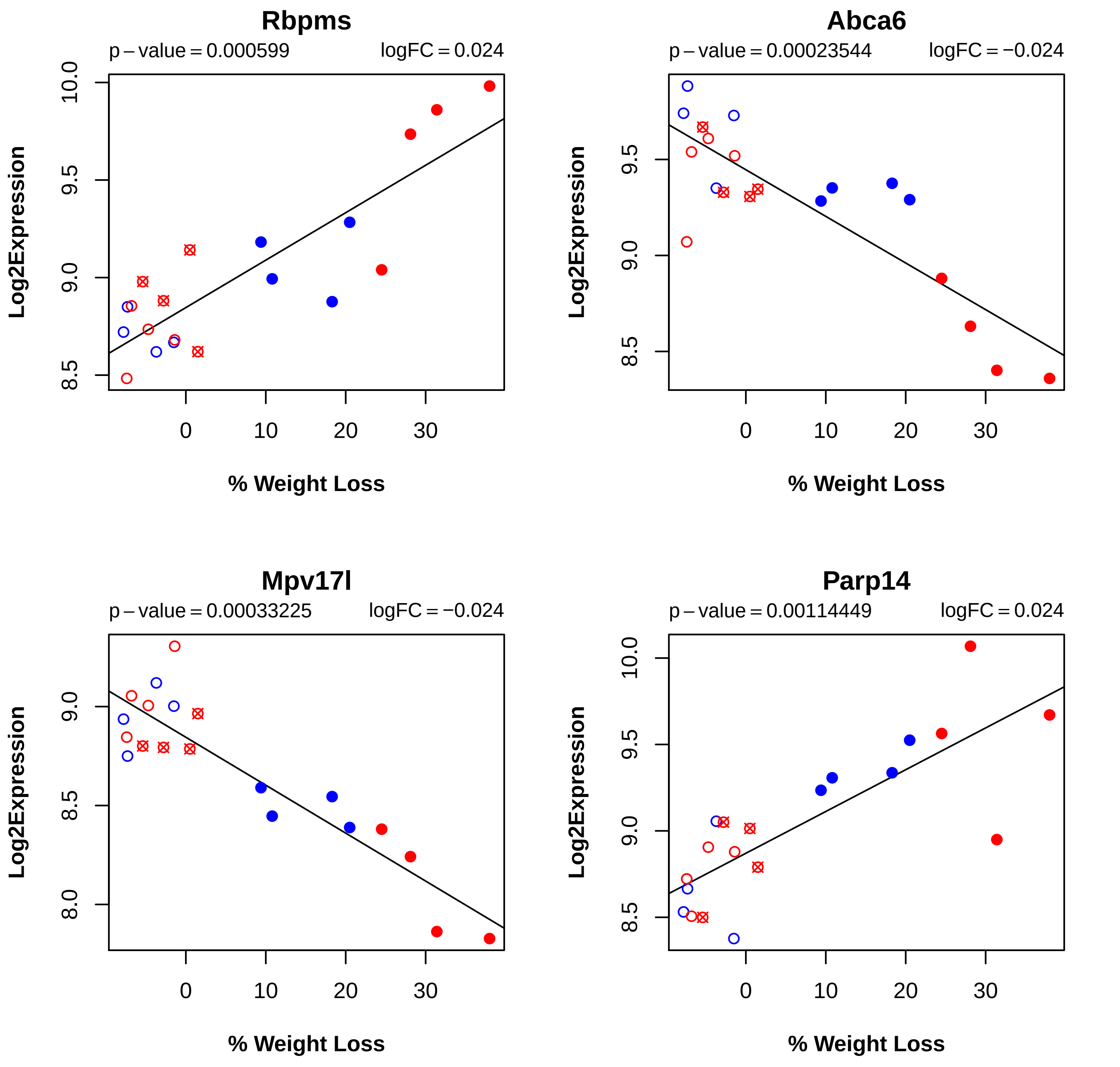
Rbpms
p – value = 0.000599
logFC = 0.024
Abca6
p – value = 0.00023544
logFC = −0.024
Mpv17l
p – value = 0.00033225
logFC = −0.024
Parp14
p – value = 0.00114449
logFC = 0.024
Log2Expression
% Weight Loss

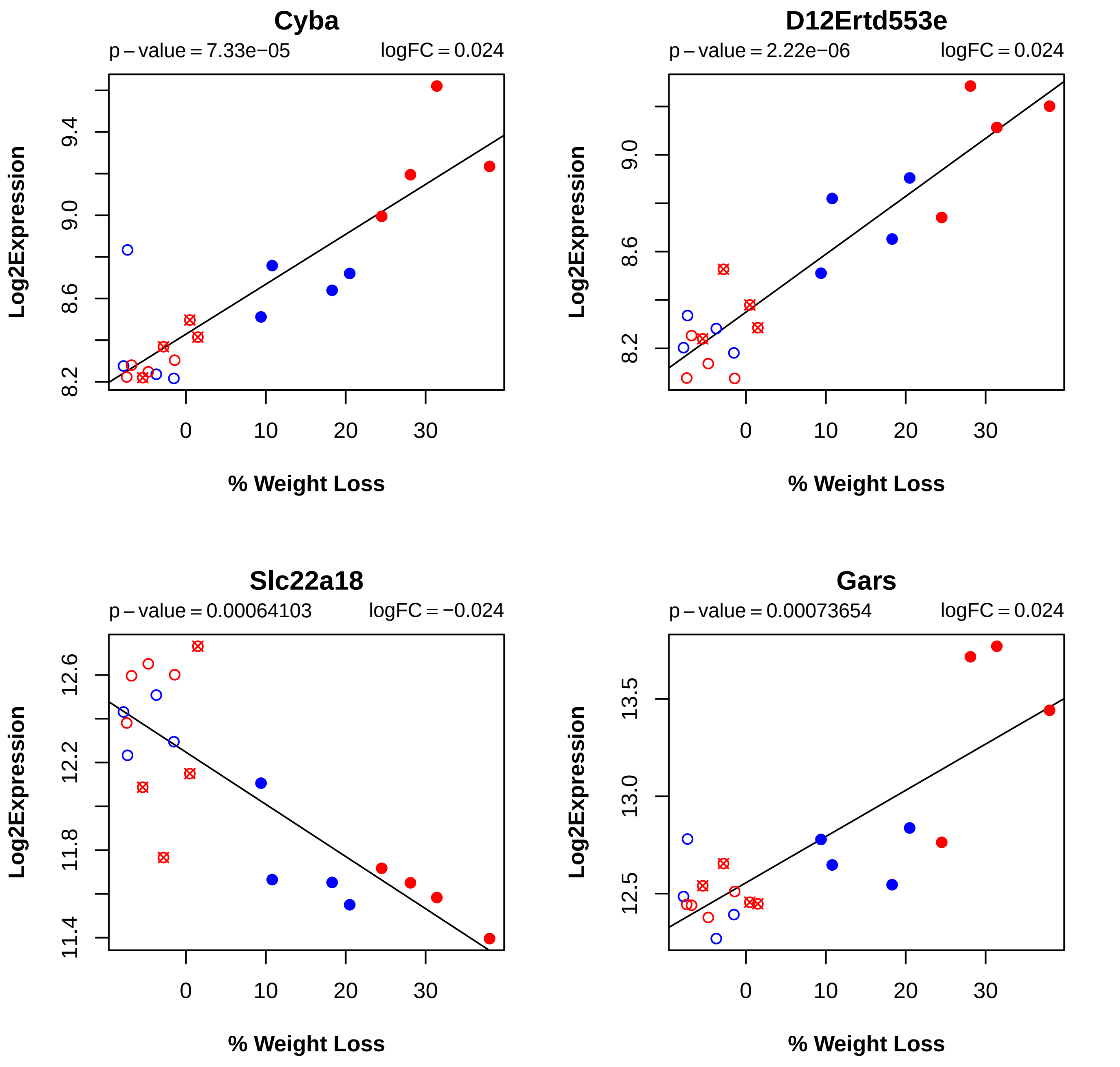
Cyba
p − value = 7.33e−05
logFC = 0.024
Log2Expression
8.2
8.6
9.0
9.4
0
10
20
30
% Weight Loss
D12Ertd553e
p − value = 2.22e−06
logFC = 0.024
Log2Expression
8.2
8.6
9.0
0
10
20
30
% Weight Loss
Slc22a18
p − value = 0.00064103
logFC = −0.024
Log2Expression
11.4
11.8
12.2
12.6
0
10
20
30
% Weight Loss
Gars
p − value = 0.00073654
logFC = 0.024
Log2Expression
12.5
13.0
13.5
0
10
20
30
% Weight Loss

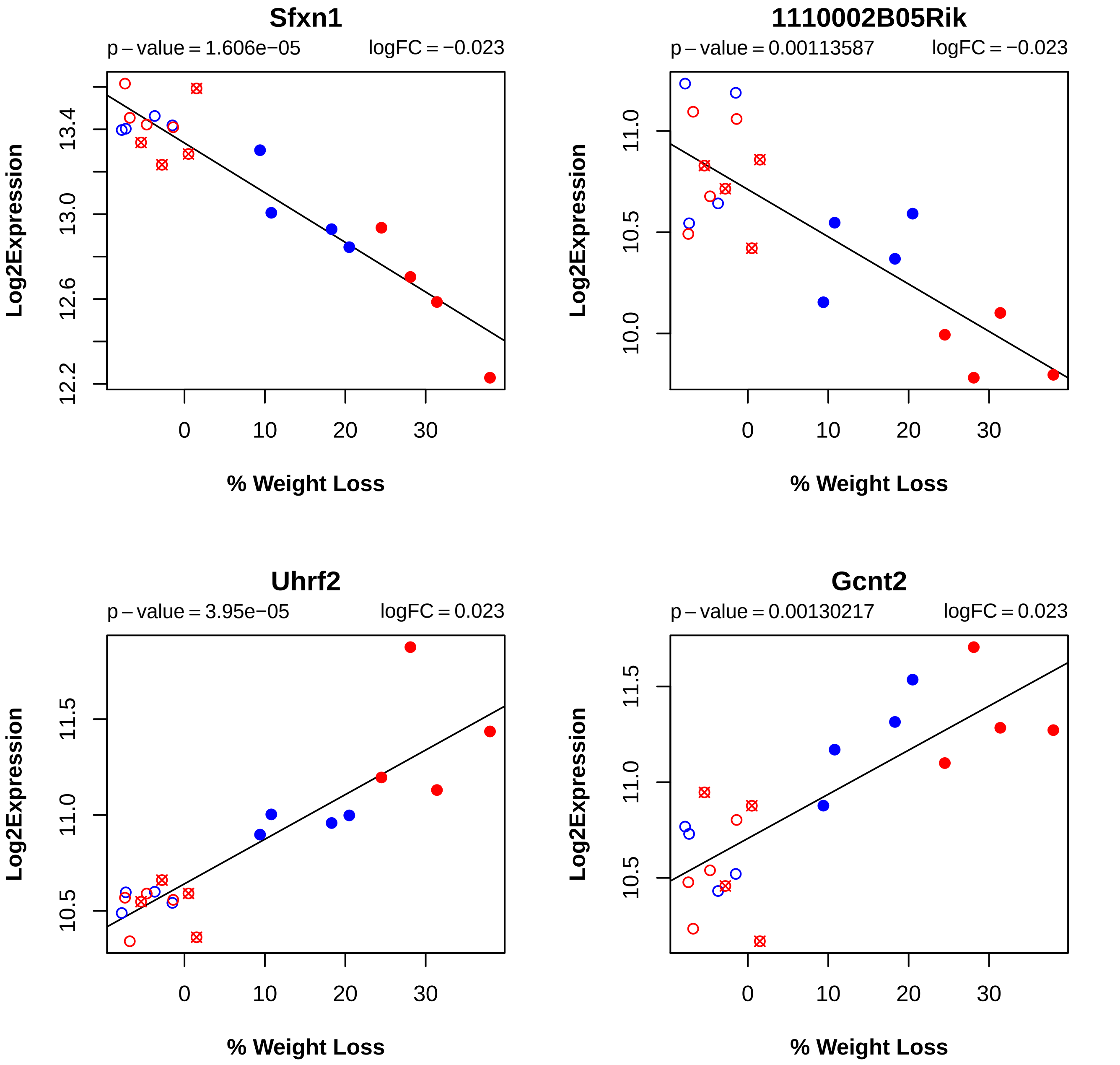

Sfxn1
p – value = 1.606e−05
logFC = −0.023
Log2Expression
12.2
12.6
13.0
13.4
0
10
20
30
% Weight Loss
1110002B05Rik
p – value = 0.00113587
logFC = −0.023
Log2Expression
10.0
10.5
11.0
0
10
20
30
% Weight Loss
Uhrf2
p – value = 3.95e−05
logFC = 0.023
Log2Expression
10.5
11.0
11.5
0
10
20
30
% Weight Loss
Gcnt2
p – value = 0.00130217
logFC = 0.023
Log2Expression
10.5
11.0
11.5
0
10
20
30
% Weight Loss

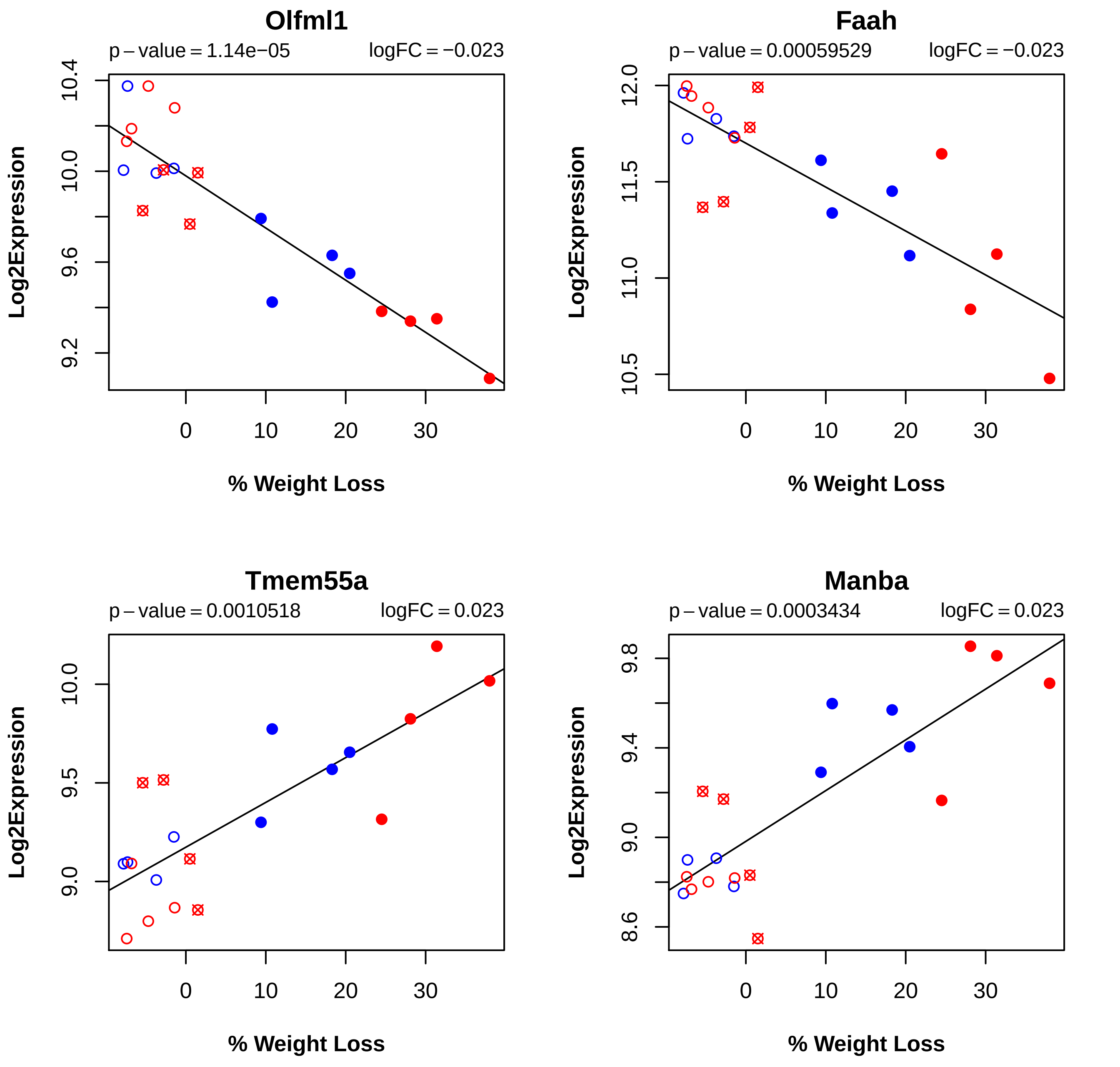

Olfml1
p − value = 1.14e−05
logFC = −0.023
Faah
p − value = 0.00059529
logFC = −0.023
Tmem55a
p − value = 0.0010518
logFC = 0.023
Manba
p − value = 0.0003434
logFC = 0.023
Log2Expression
% Weight Loss

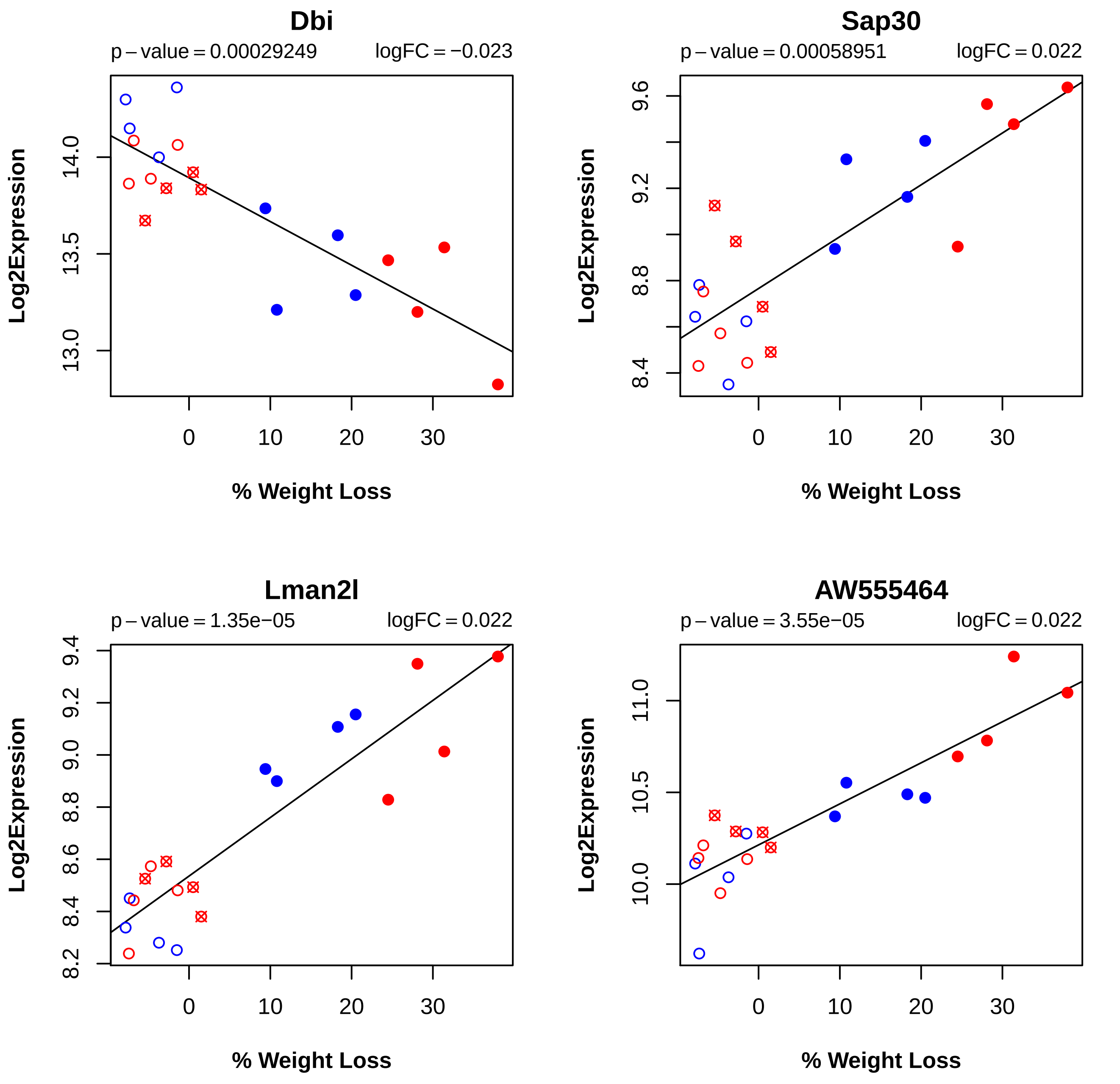

Dbi
p – value = 0.00029249
logFC = −0.023
Log2Expression
% Weight Loss
Sap30
p – value = 0.00058951
logFC = 0.022
Log2Expression
% Weight Loss
Lman2l
p – value = 1.35e−05
logFC = 0.022
Log2Expression
% Weight Loss
AW555464
p – value = 3.55e−05
logFC = 0.022
Log2Expression
% Weight Loss

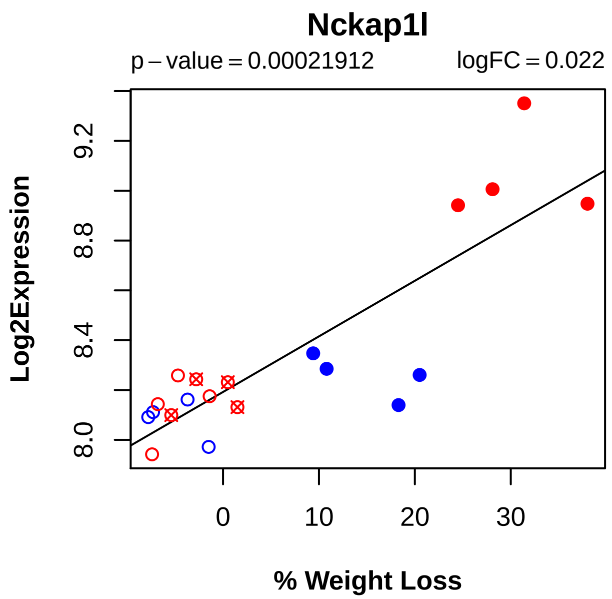
Nckap1l
p − value = 0.00021912
logFC = 0.022
Log2Expression
8.0
8.4
8.8
9.2
0
10
20
30
% Weight Loss

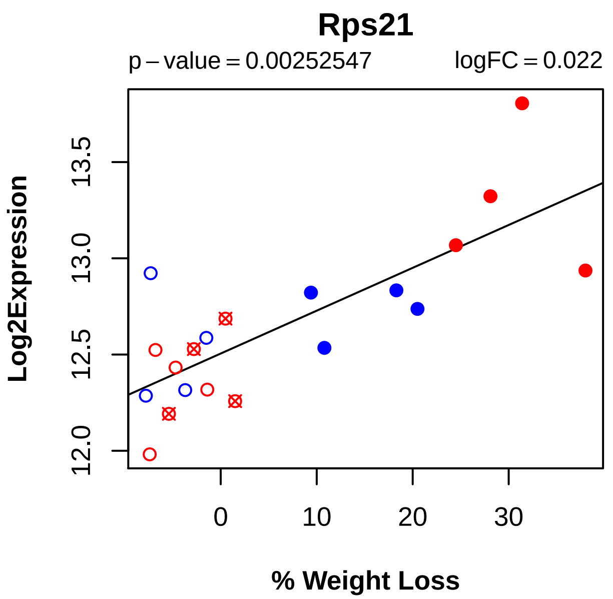
Rps21
p − value = 0.00252547
logFC = 0.022
Log2Expression
12.0
12.5
13.0
13.5
0
10
20
30
% Weight Loss

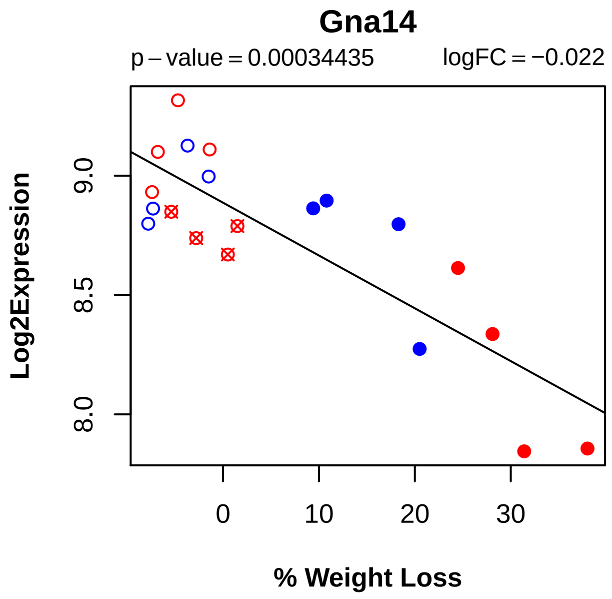
Gna14
p − value = 0.00034435
logFC = −0.022
Log2Expression
8.0
8.5
9.0
0
10
20
30
% Weight Loss

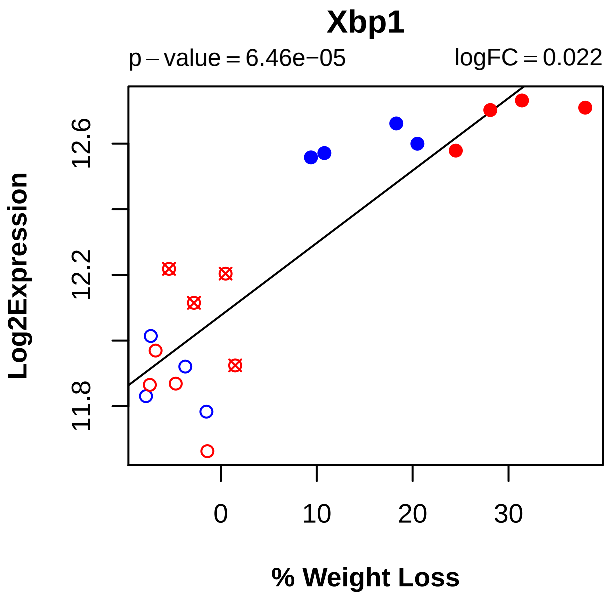
Xbp1
p − value = 6.46e−05
logFC = 0.022
Log2Expression
11.8
12.2
12.6
0
10
20
30
% Weight Loss

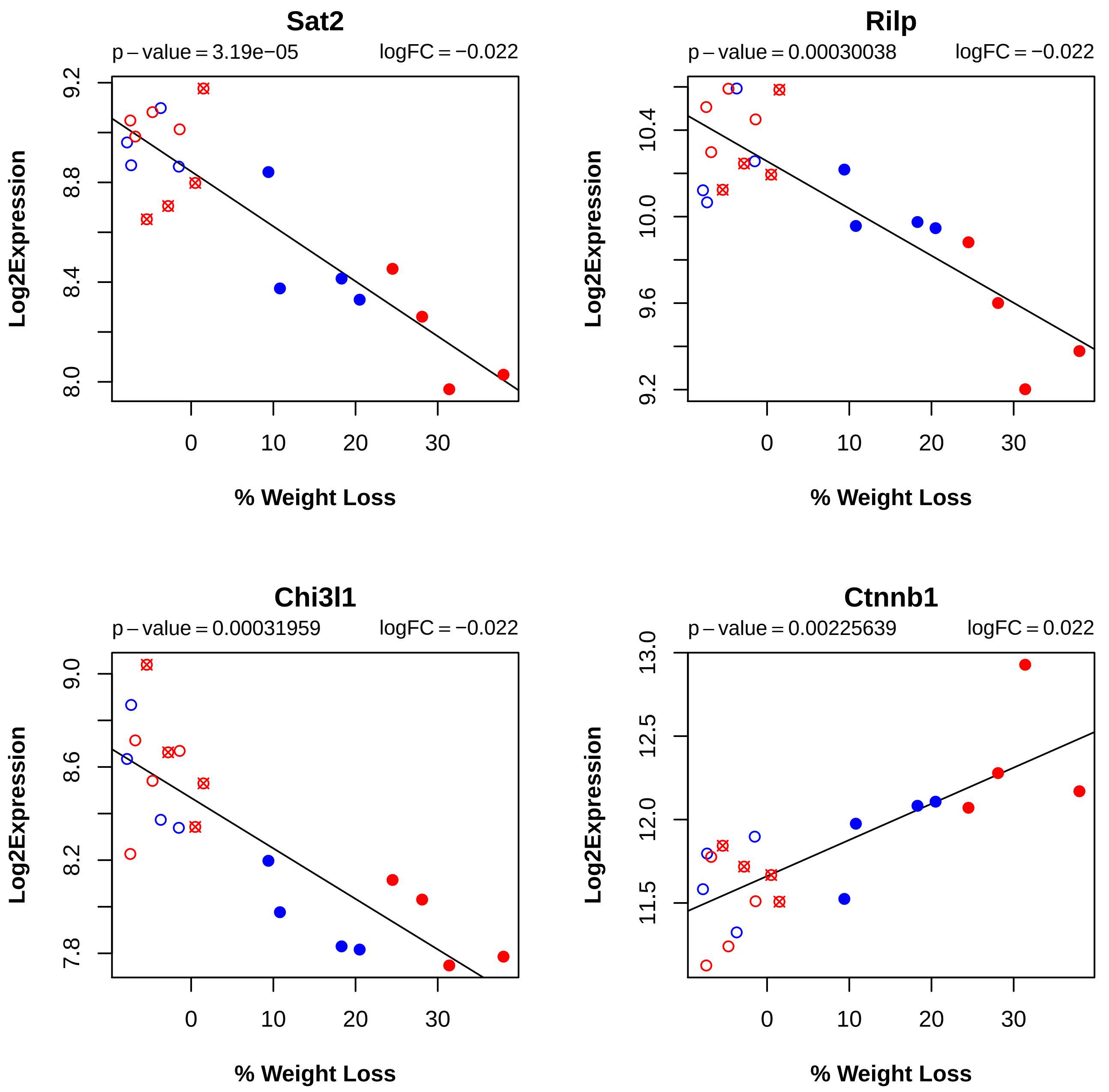
Sat2
p – value = 3.19e−05
logFC = −0.022
Log2Expression
% Weight Loss
Rilp
p – value = 0.00030038
logFC = −0.022
Log2Expression
% Weight Loss
Chi3l1
p – value = 0.00031959
logFC = −0.022
Log2Expression
% Weight Loss
Ctnnb1
p – value = 0.00225639
logFC = 0.022
Log2Expression
% Weight Loss

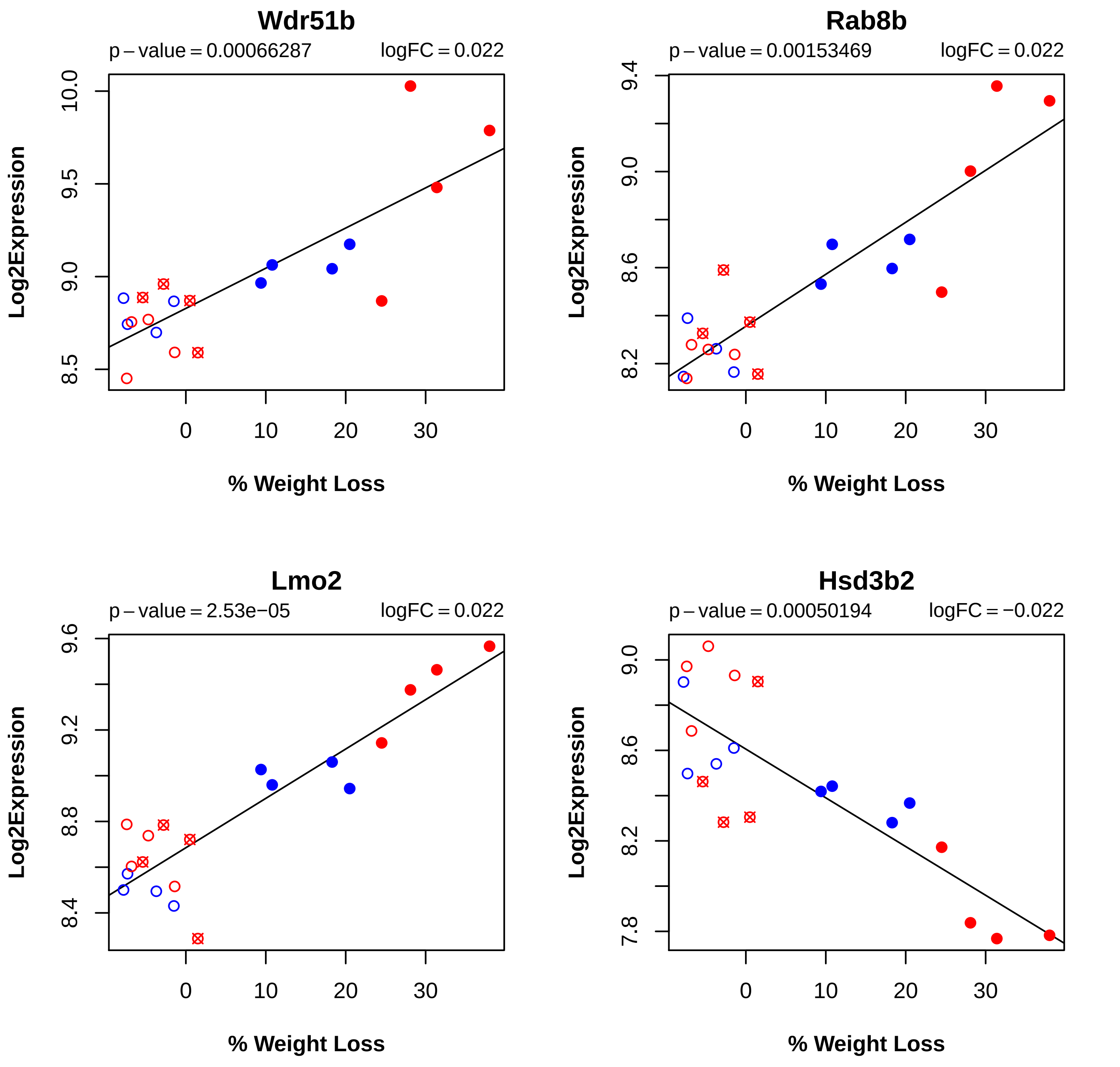

Wdr51b
p − value = 0.00066287
logFC = 0.022
Log2Expression
% Weight Loss
Rab8b
p − value = 0.00153469
logFC = 0.022
Log2Expression
% Weight Loss
Lmo2
p − value = 2.53e−05
logFC = 0.022
Log2Expression
% Weight Loss
Hsd3b2
p − value = 0.00050194
logFC = −0.022
Log2Expression
% Weight Loss

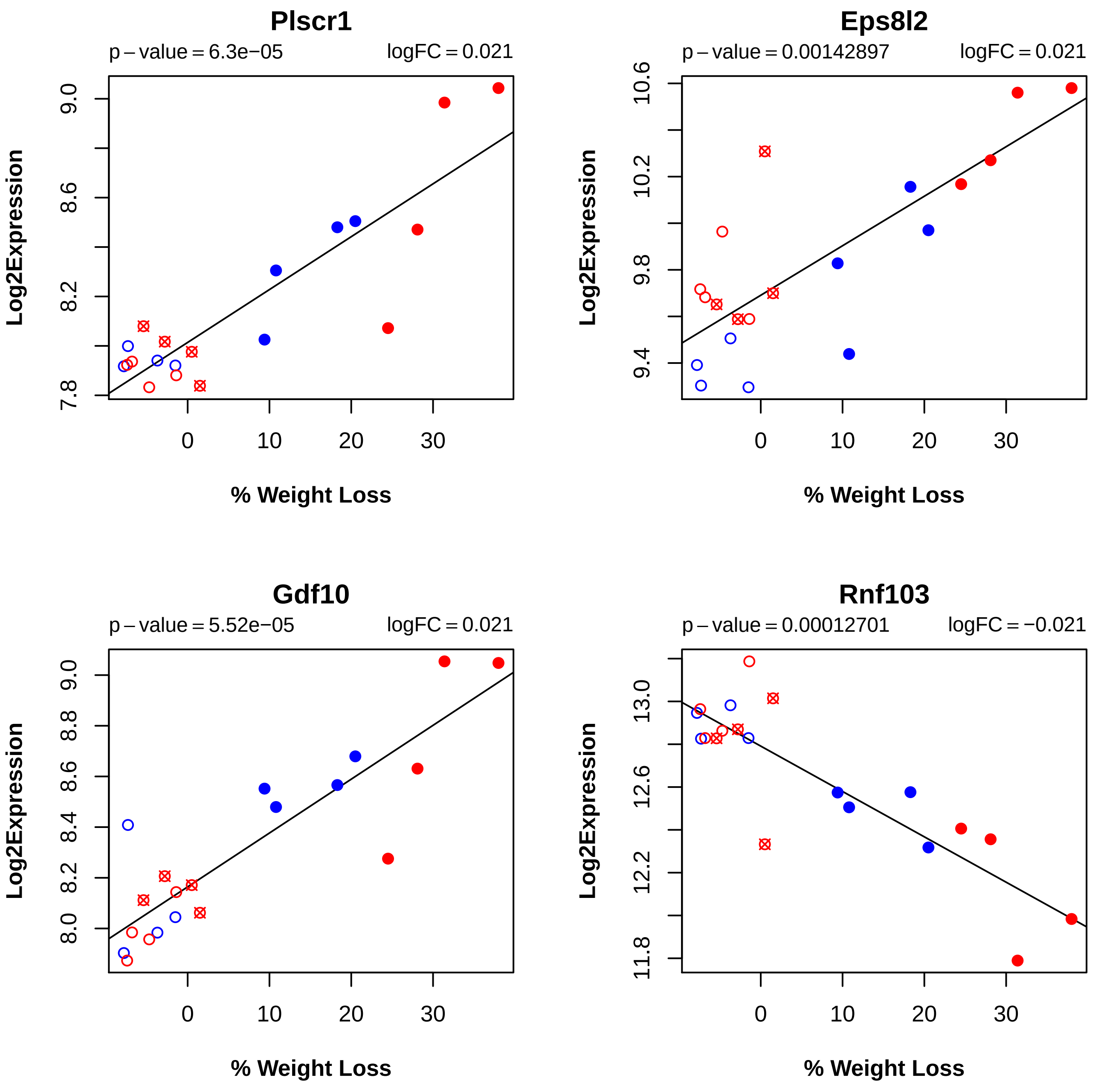
Plscr1
p – value = 6.3e−05
logFC = 0.021
Log2Expression
% Weight Loss
7.8
8.2
8.6
9.0
0
10
20
30
Eps8l2
p – value = 0.00142897
logFC = 0.021
Log2Expression
% Weight Loss
9.4
9.8
10.2
10.6
0
10
20
30
Gdf10
p – value = 5.52e−05
logFC = 0.021
Log2Expression
% Weight Loss
8.0
8.2
8.4
8.6
8.8
9.0
0
10
20
30
Rnf103
p – value = 0.00012701
logFC = −0.021
Log2Expression
% Weight Loss
11.8
12.2
12.6
13.0
0
10
20
30

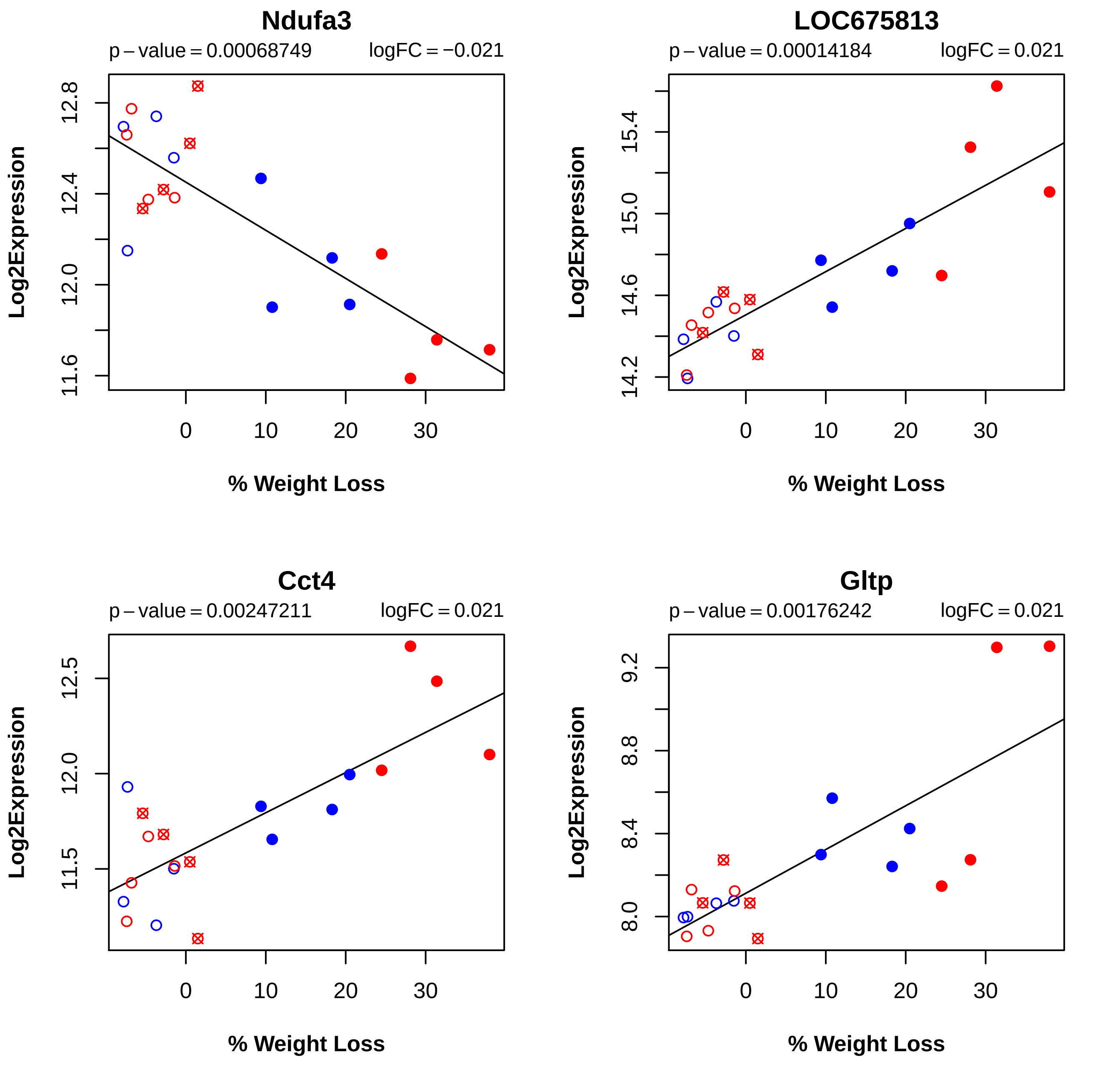

Ndufa3
p – value = 0.00068749
logFC = −0.021
Log2Expression
12.8
12.4
12.0
11.6
0
10
20
30
% Weight Loss
LOC675813
p – value = 0.00014184
logFC = 0.021
Log2Expression
15.4
15.0
14.6
14.2
0
10
20
30
% Weight Loss
Cct4
p – value = 0.00247211
logFC = 0.021
Log2Expression
12.5
12.0
11.5
0
10
20
30
% Weight Loss
Gltp
p – value = 0.00176242
logFC = 0.021
Log2Expression
9.2
8.8
8.4
8.0
0
10
20
30
% Weight Loss

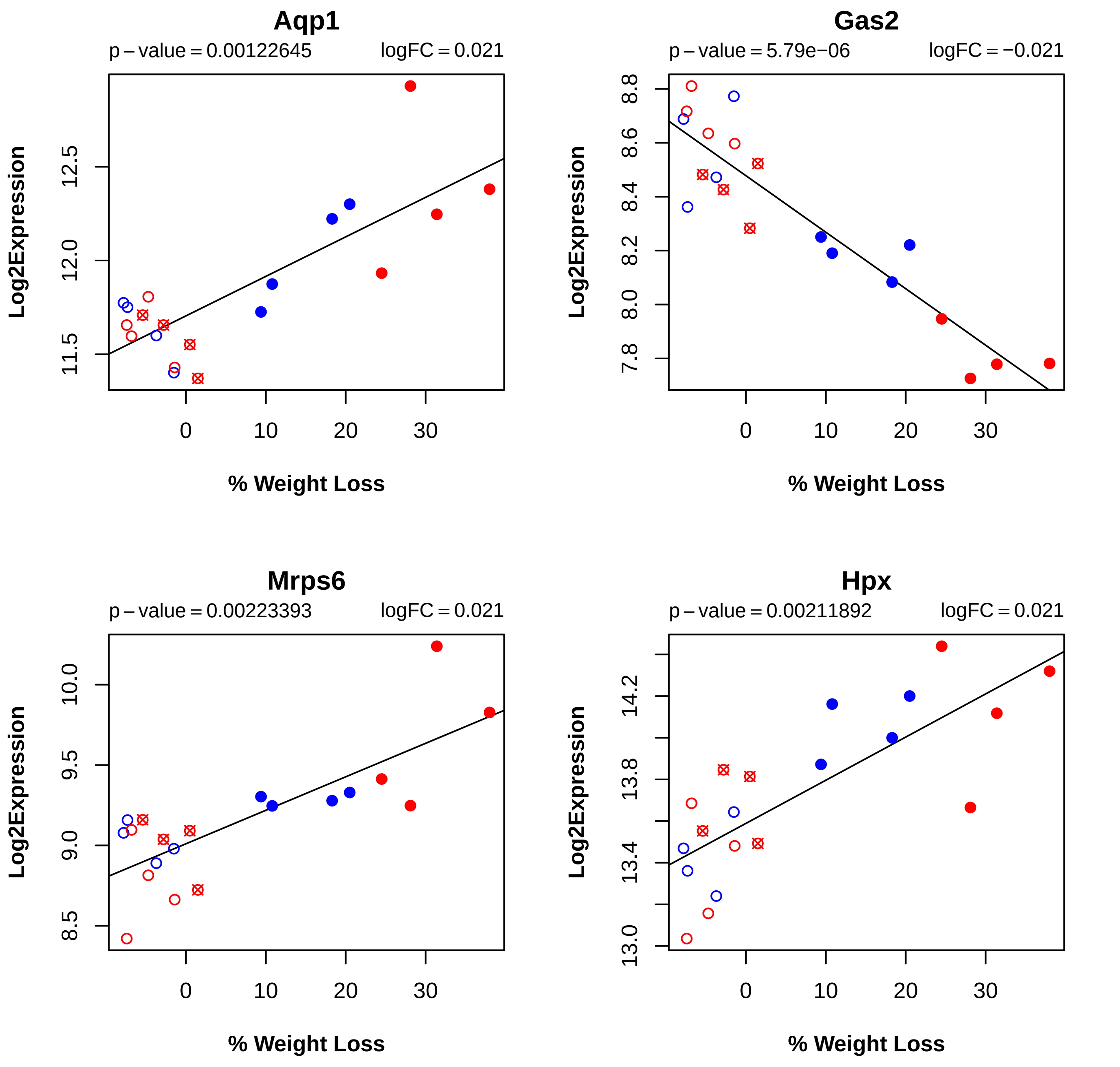

Aqp1
p – value = 0.00122645
logFC = 0.021
Log2Expression
11.5
12.0
12.5
0
10
20
30
% Weight Loss
Gas2
p – value = 5.79e−06
logFC = −0.021
Log2Expression
7.8
8.0
8.2
8.4
8.6
8.8
0
10
20
30
% Weight Loss
Mrps6
p – value = 0.00223393
logFC = 0.021
Log2Expression
8.5
9.0
9.5
10.0
0
10
20
30
% Weight Loss
Hpx
p – value = 0.00211892
logFC = 0.021
Log2Expression
13.0
13.4
13.8
14.2
0
10
20
30
% Weight Loss

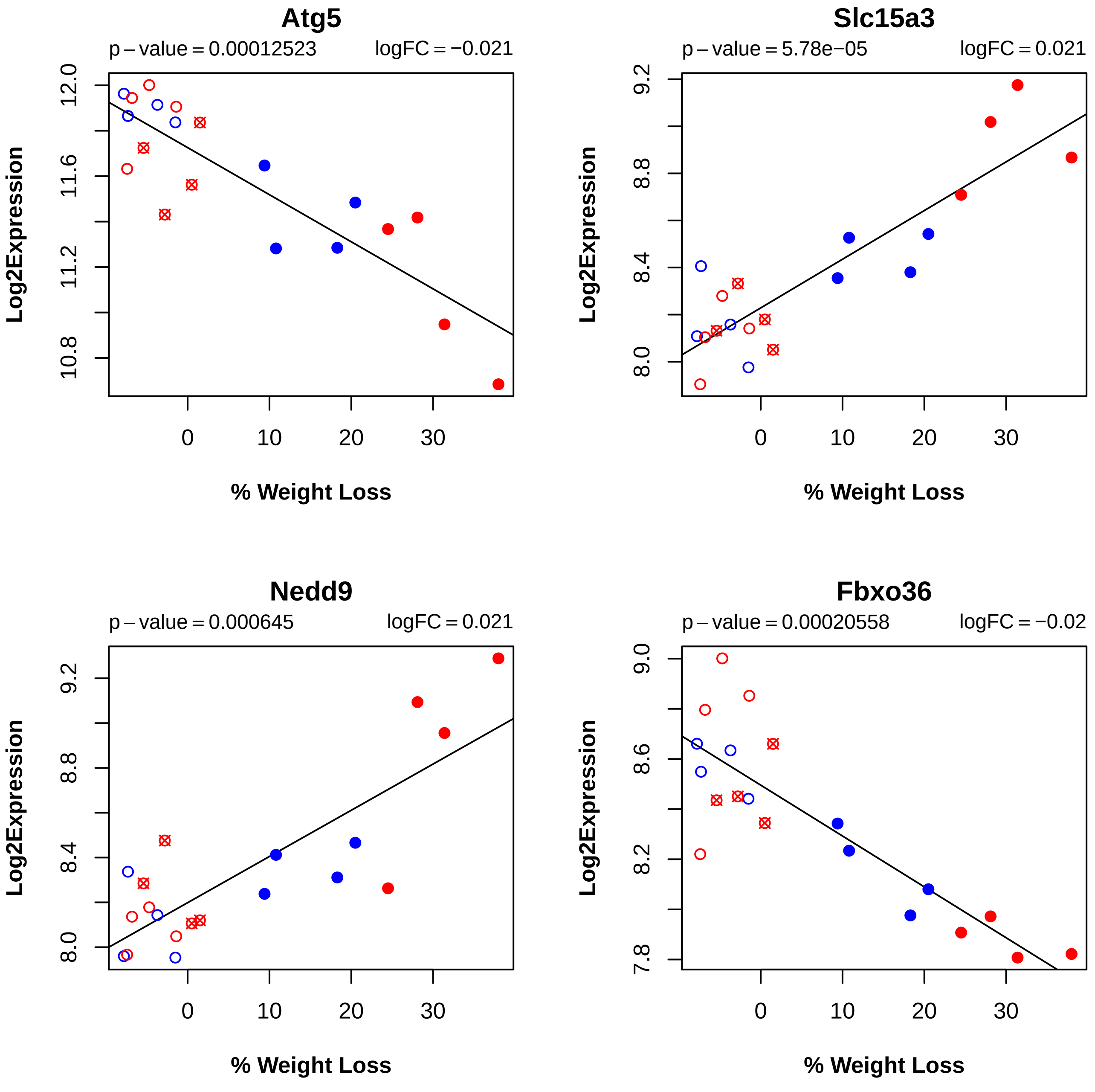

Atg5
p – value = 0.00012523
logFC = −0.021
Log2Expression
% Weight Loss
Slc15a3
p – value = 5.78e−05
logFC = 0.021
Log2Expression
% Weight Loss
Nedd9
p – value = 0.000645
logFC = 0.021
Log2Expression
% Weight Loss
Fbxo36
p – value = 0.00020558
logFC = −0.02
Log2Expression
% Weight Loss

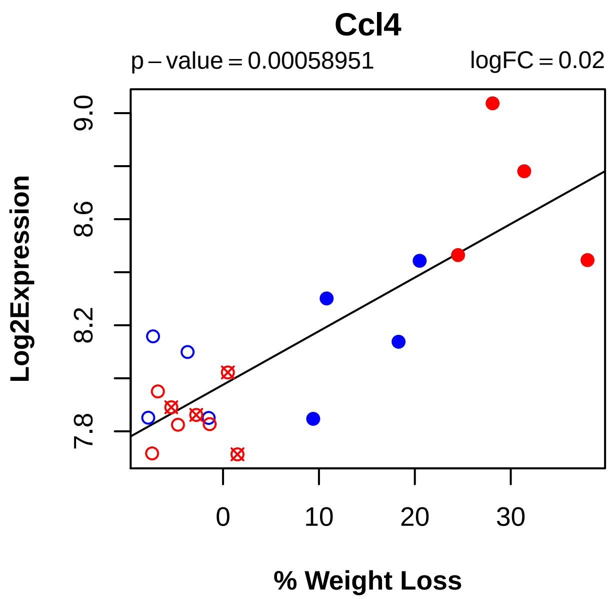
Ccl4
p − value = 0.00058951
logFC = 0.02
Log2Expression
9.0
8.6
8.2
7.8
0
10
20
30
% Weight Loss

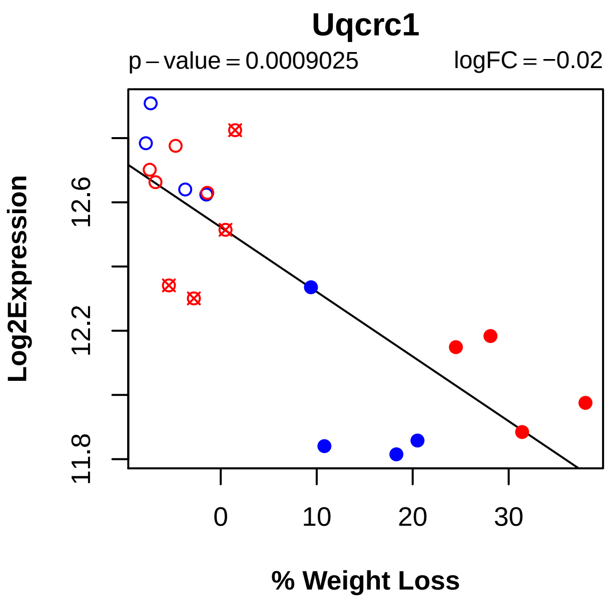
Uqcrc1
p − value = 0.0009025
logFC = −0.02
Log2Expression
12.6
12.2
11.8
0
10
20
30
% Weight Loss
