# Supplementary material for: Hepatic Gene Expression Profiling Reveals Key Pathways Involved in Leptin-Mediated Weight Loss in ob/ob Mice
Source: PLoS One. 2010 Aug 16;5(8):e12147. doi: 10.1371/journal.pone.0012147 (PMC2922341; doi:10.1371/journal.pone.0012147)

Gene Ontology Analyses - Supplemental Data

Using the GOTree Machine web application, the 214 differentially expressed genes were categorized as being associated with specific biological processes, molecular functions and cellular compartments. Statistically significant over represented biological processes were determined using the hypergeometric test.

Downregulated Cellular Compartments

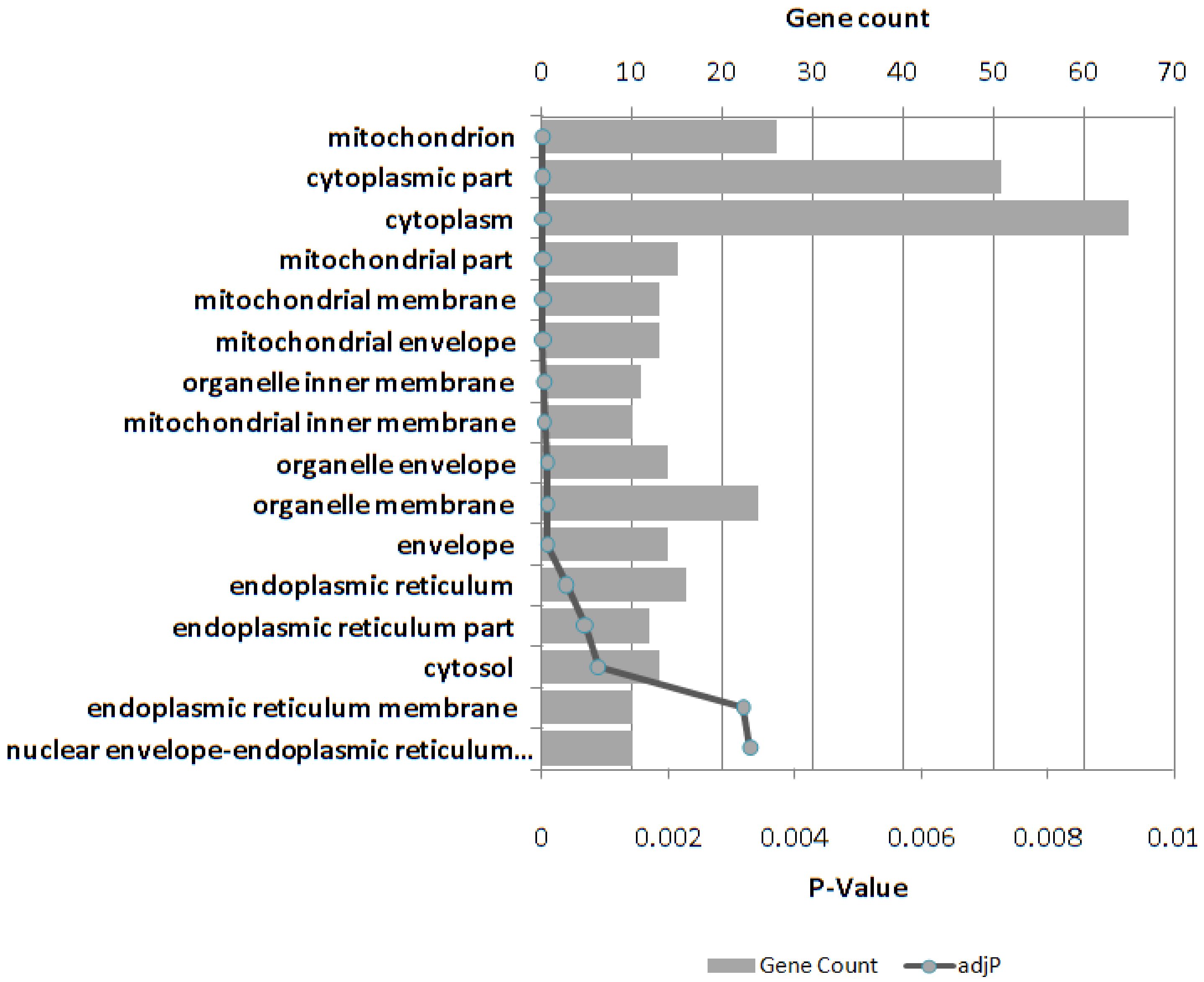

Upregulated Cellular Compartments

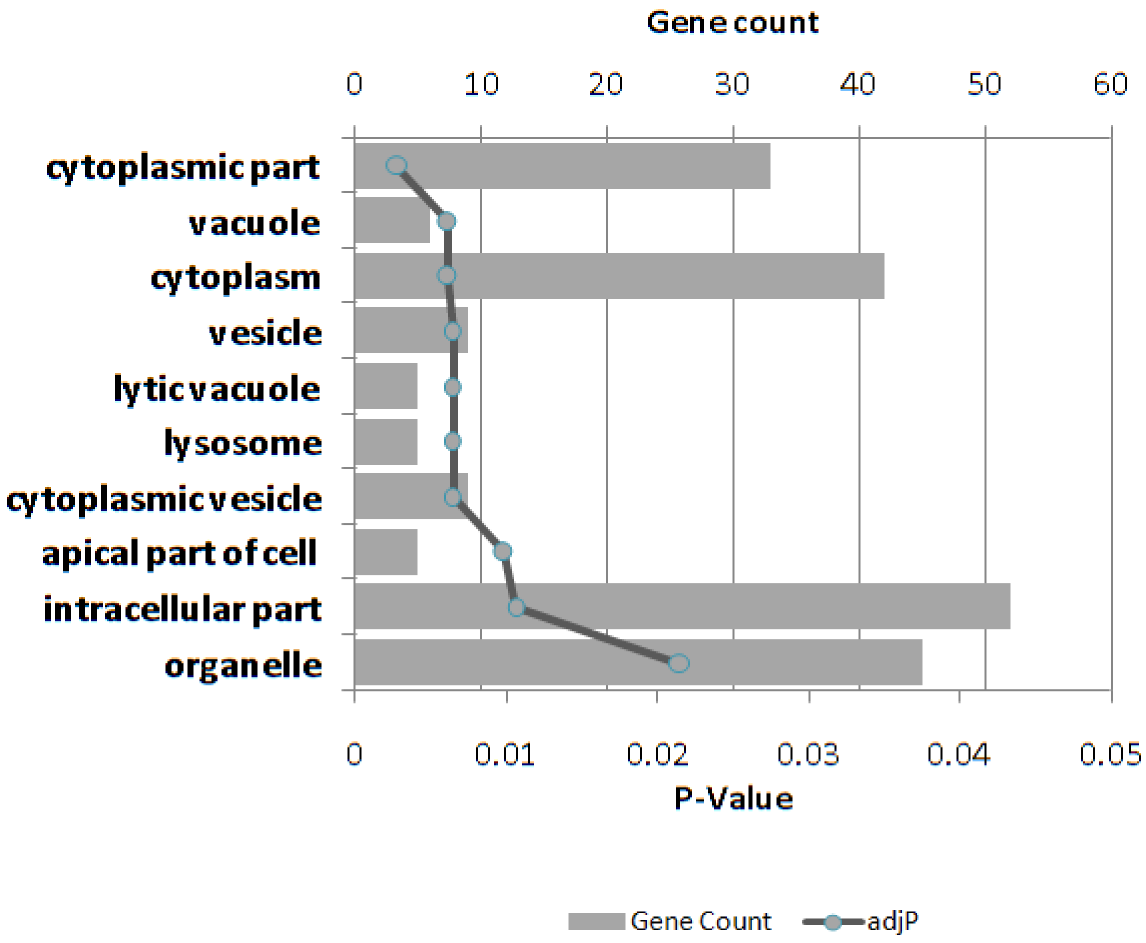

Downregulated Biological Processes

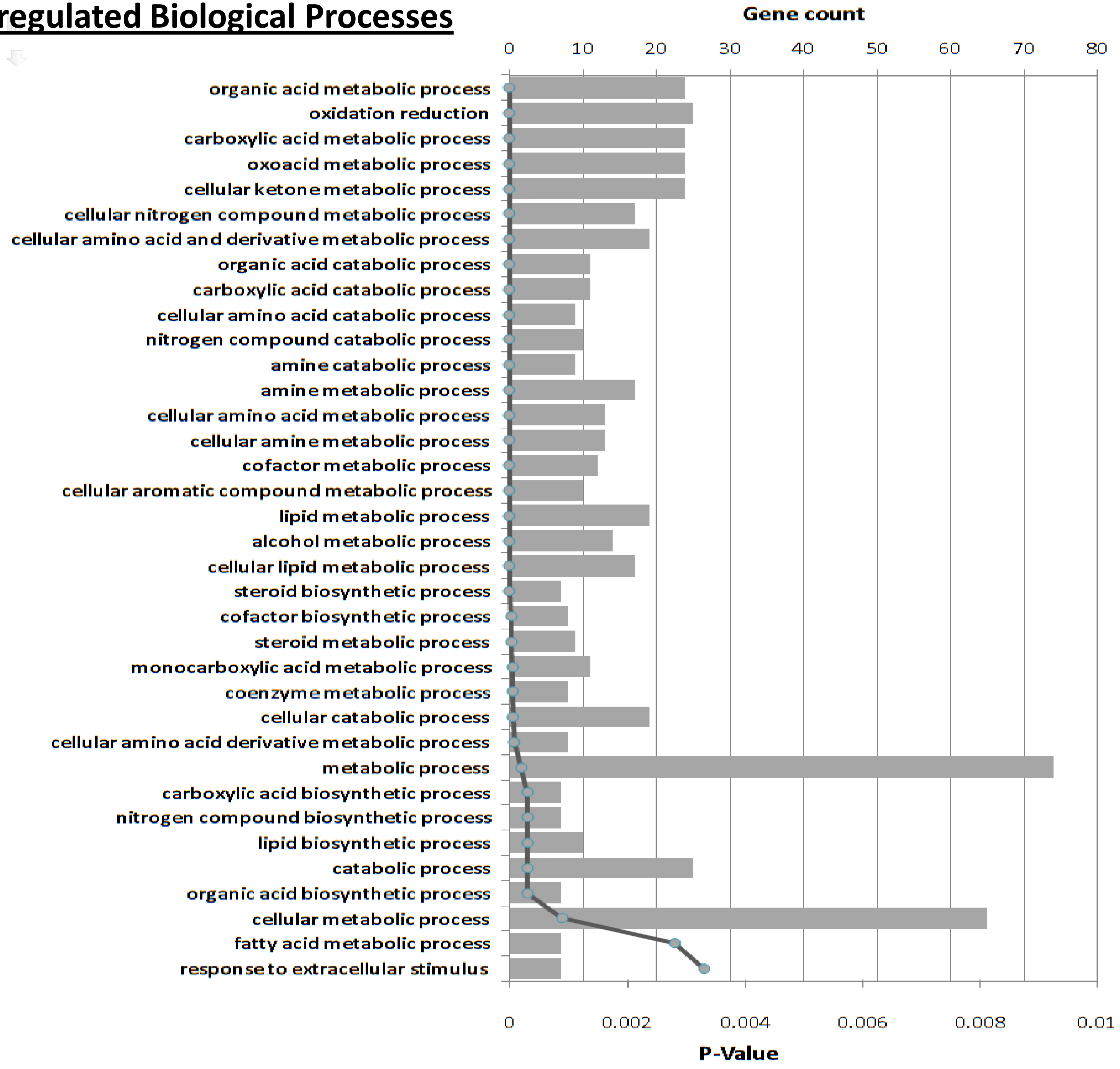

Upregulated Biological Processes

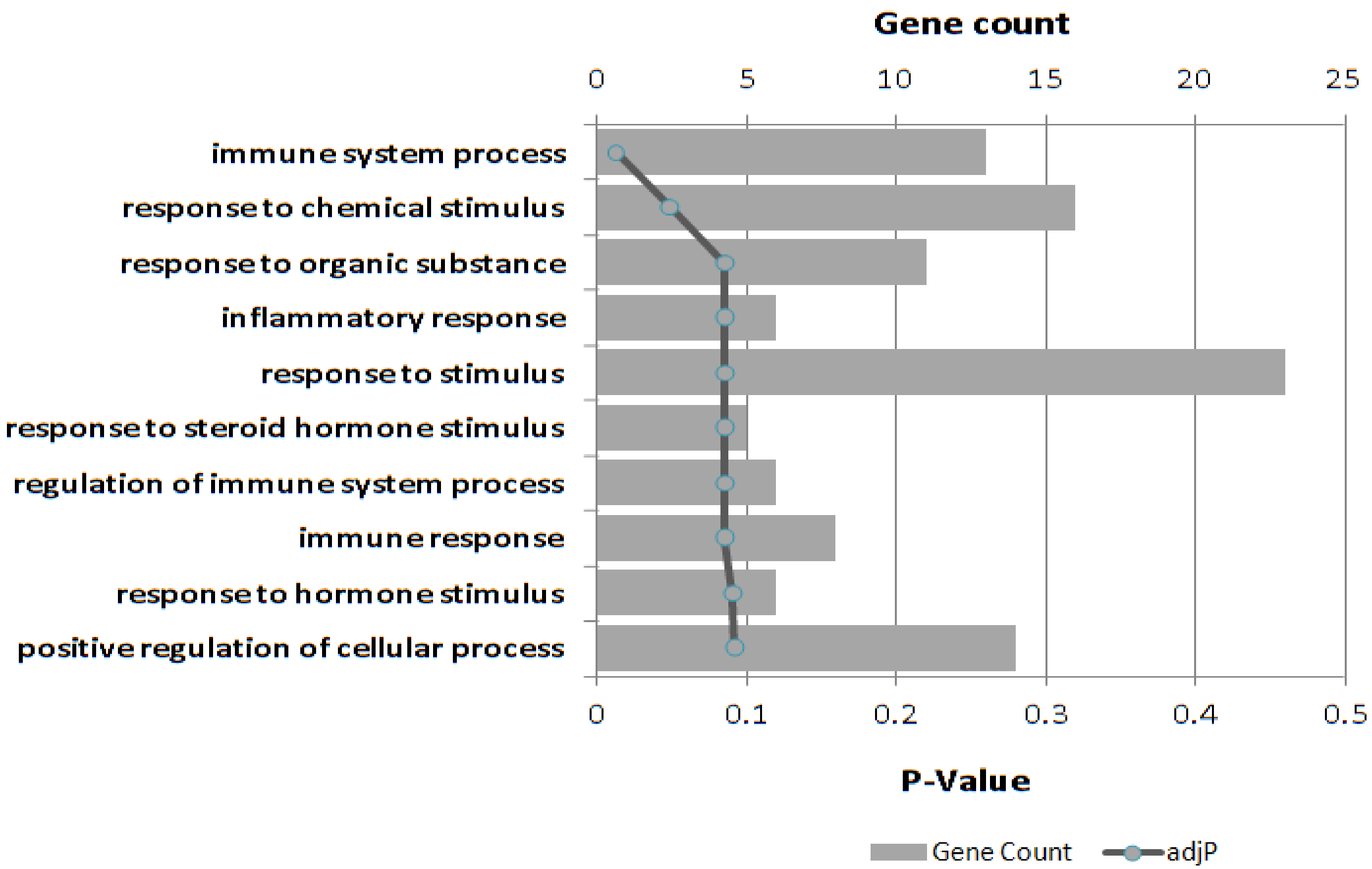

Downregulated Molecular Functions

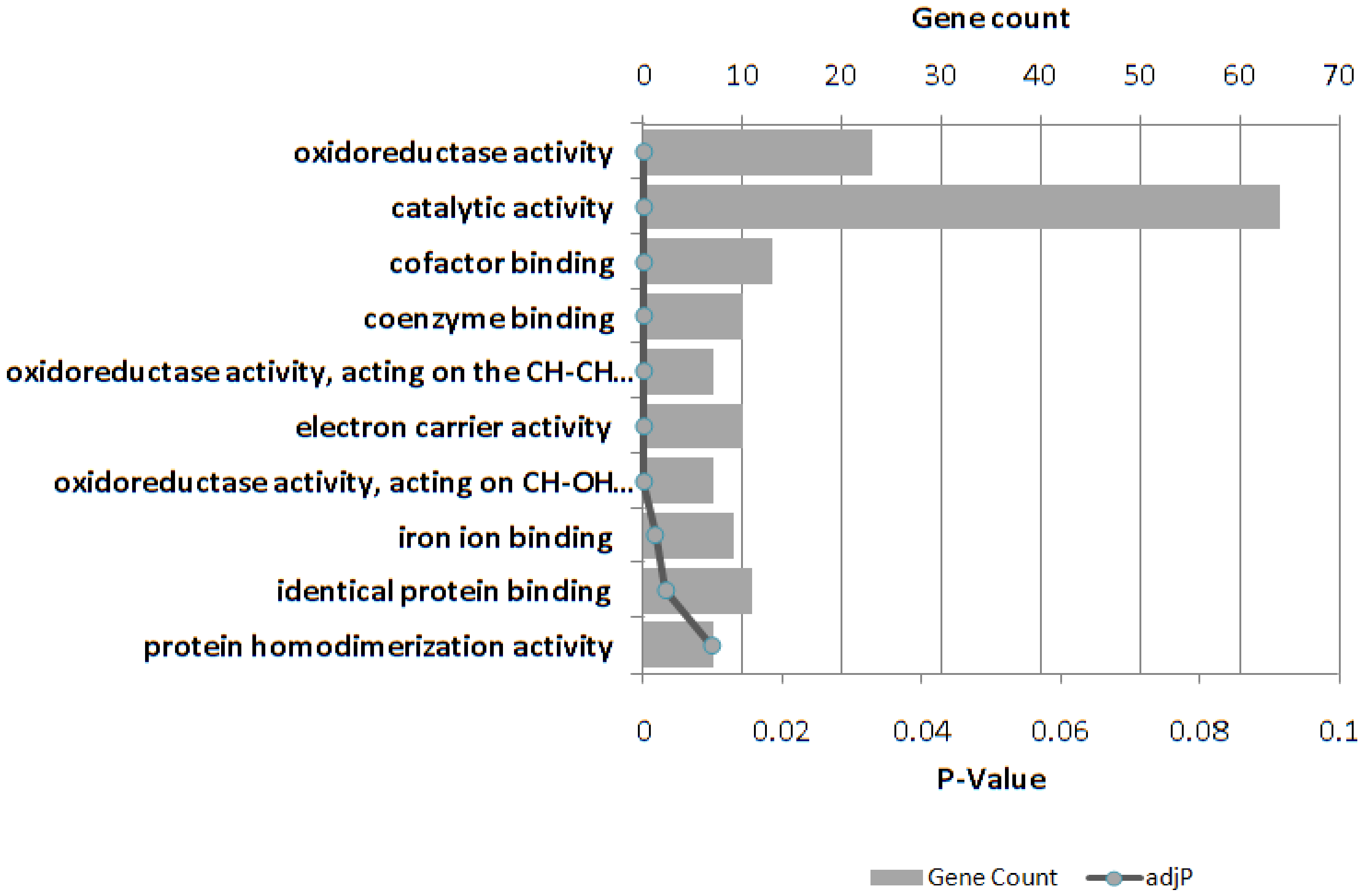

Upregulated Molecular Functions

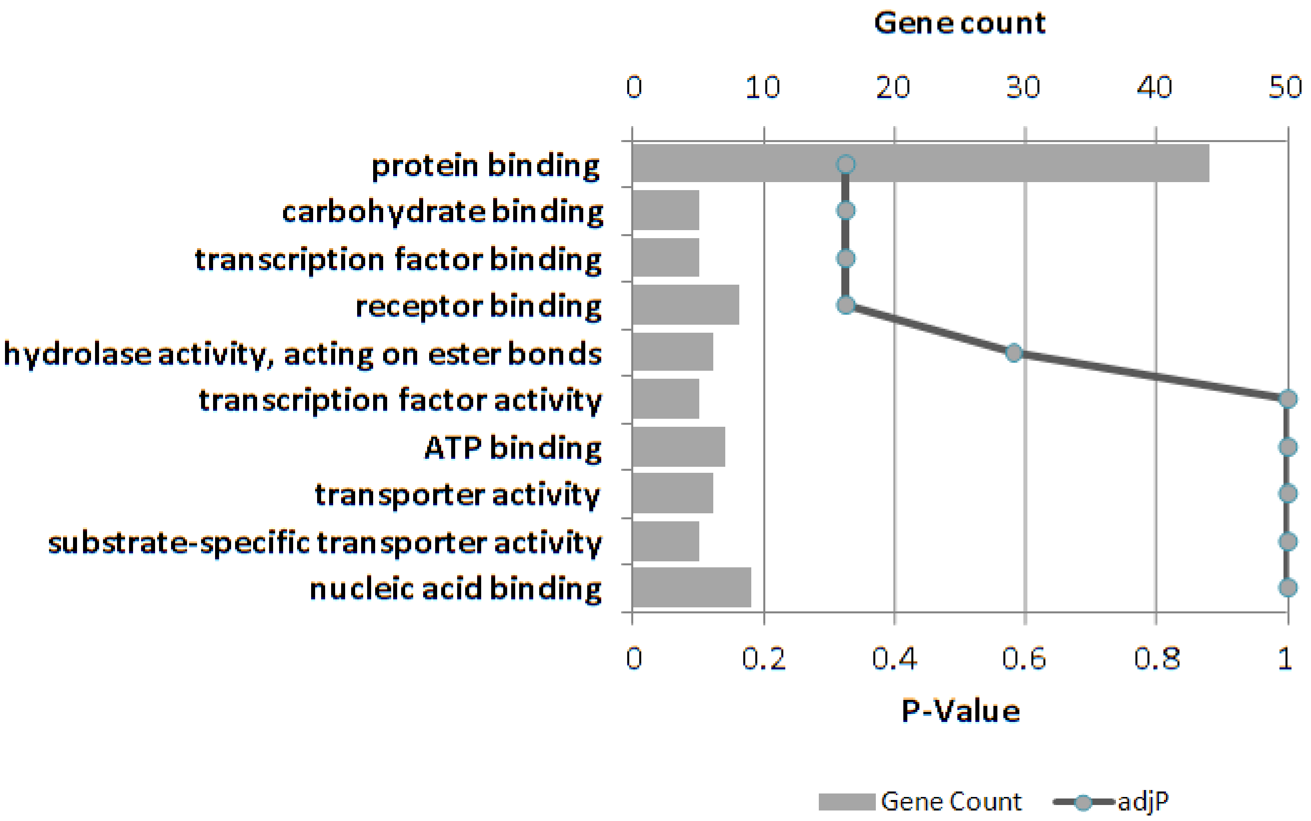

Supplement: File S3 — Gene Ontology.pdf. Biological processes, molecular functions and cellular compartments of hepatic genes regulated by leptin treatment. (0.24 MB PDF) [file pone.0012147.s003.pdf]
