# Supplementary material for: Hepatic Gene Expression Profiling Reveals Key Pathways Involved in Leptin-Mediated Weight Loss in ob/ob Mice
Source: PLoS One. 2010 Aug 16;5(8):e12147. doi: 10.1371/journal.pone.0012147 (PMC2922341; doi:10.1371/journal.pone.0012147)
Supplement: File S4 — Networks.pdf. Top molecular networks and their biological functions affected by leptin treatment. Network analysis was performed using Ingenuity Pathway Analysis software. (1.17 MB PDF) [file pone.0012147.s004.pdf]

S4: Top molecular networks and biological functions affected by leptin treatment.

ID	Gene in the network	Associated Network Functions	Score	# genes
1	26s Proteasome, ↓**ACAT2,** ↑**ACOT1 (includes EG:26897),**Actin, ↓**AES,** ↓**AHCY,** Akt, Ap1, ↑**AQP1,** ↓**ARG1,** ↓**ASS1,** ↓**ATG5 (includes EG:9474),**↓**BAAT,** BCR, Calmodulin, Calpain, Caspase, ↑**CCL4,**↑**CD44,** Ck2, Collagen type I, Collagen type IV, Creb, ↑**CTNNB1,** ↓**CYB5R3,**↑**CYBA,**↓**CYP17A1,**↓**CYP2C29,**Cytochrome c, ↓**DBI,**↑**DUSP6,**↑**EDNRB,** ↑**EGFR,**↑**EPS8L2,**ERK, ERK1/2, ↓**ES22,**↑**ETS2,**↓**FAAH,** ↑**FCER1G,**Fibrinogen, FOXS1, FSH, ↓**GAS2,** ↓**GCK,**↑**GCNT2,**glutathione transferase, ↓**GPT,** Growth hormone, GST, ↓**GSTA3,** ↓**GSTA5*,** ↓**GSTT1,** ↓**GSTT3,** ↓**HAMP,** hCG, Histone h3, ↑**HLA-DQB1,** ↑**HMGA1,** ↓**HMGCS1,** ↑**HPX,**↓**HSD17B10,**↓**HSD3B1,**Hsp90, ↑**HSP90AB1,**↑**HSP90B1,** IFN Beta, ↑**IGFBP2,**IgG, IL1, IL12 (complex), Immunoglobulin, Insulin, Interferon alpha, Jnk, ↓**KLK2,**↓**KYNU,**LDL, Lh, ↑**LMO2,**↑**LPL,**↑**LYZ,**↑**MAGED1,** Mapk, ↑**MAT2A,** Mek, Mmp, ↑**MMP12,** ↑**MRPS6 (includes EG:64968),**↓**MVD,** N-cor, ↓**NDUFA3,**↑**NEDD9,** NFkB (complex),↑**NUPR1,**P38 MAPK, palmitoyl-CoA hydrolase, ↓**PCSK9,**Pdgf, PDGF BB, peptidase, Pi3-kinase, PI3K, Pka, Pkc(s), PLA2, ↑**PLA2G12A,** ↓**PLA2G4F,** ↓**PLS3,** ↑**PLSCR1,** ↓**PPP1R1B,**↓**PSEN2,**↑**RAB8B,** Ras homolog, ↓**RILP,**↑**RPS11,** Rxr, ↑**SAP30,** ↓**SCD,** SENP5 (includes EG:205564),↑**SERPINB6,**↓**SERPINC1,**Shc, ↓**SLC25A1,** ↓**SLC29A1,** ↑**SLC40A1,** ↑**SOCS2,** STAT5a/b, Tgf beta, ↑**TGFBI,**↓**THRSP,**↑**TMEM55A,** ↓**TPMT,** ↓**TSC22D3,**↑**UNG,**↓**UQCRC1,**↑**USP18,** Vegf, ↑**XBP1,**↓**ZFP36**	Lipid Metabolism, Small Molecule Biochemistry, Cell-To-Cell Signaling and Interaction	145	84
2	↓**9130409I23RIK,**↓**AASS,** ↓**ABAT,** ↓**ACSS2,** AFP, ↑**AGPAT9,** AHR, ALDH16A1, ALDH3A2, ALDH5A1, ↓**ALDH6A1,** AMD1, aminomethyltransferase, ↓**ANKRD40,**APH1B, ARF4, ↓**ARG1,** BAMBI, beta-estradiol, ↑**C1QB,**CCBP2, ↑**CCT4,** CDH13, CDH15, CELSR2, CGB, cholesterol, ↓**CISD1,** CMA1, CNN2, COL16A1, CRK, ↓**CSTF3,** CTSD, CYB5B, CYP27A1, DHRS1, dimethylglycine dehydrogenase, ↓**DMGDH,**↓**DPYD,**↑**DUSP6,** ECM1, EIF4E, EIF4EBP1, ENPP1, ethanol, FASTK, FCAR, FGB, FZD7, ↓**GAMT,** GCC2, ↓**GCDH,**↑**GDF10,** ↓**GLS2,**↓**GNA14,**↑**GPNMB,**↓**GSTT1,** ↓**HAMP,** ↓**HAO2,** HGF, HPGD, HRAS, HSD17B7, IL5, IL6, IL13RA2, IL17F, INSIG2, KDELR3, KIAA0182, ↑**KLHDC2,**L-glutamic acid,↑**LEPROT,**LIF, LIMA1, LIMS1, LRRC8C, ↓**LSS,** MICAL1, MOGS, ↓**MPV17L,** MSR1, ↓**MVD,** MYC, NME3, ↓**NNMT,**NOVA1, NOX3, ↑**NUPR1,**oxidoreductase, PARP, ↑**PARP14,** ↓**PARVA,** PDLIM4, ↓**PLS3,** PRL, ↓**PRODH,** PTEN, ↑**RAB8B,** ↑**RBPMS,**↓**RCAN2,**↓**RDH16,** REG3G, ↓**RNF103,** ↓**ROBO1,**↑**RPL13A,** ↑**RPL27A,** ↑**RPS19,** ↑**RPS21,** ↓**SARDH,** ↑**SEL1L3,**↓**SELENBP1,** SEMA3C, ↑**SERPINA7,**↓**SFXN1,**↓**SIGMAR1,** SLC12A6, ↓**SLC25A1,** SLC2A4, ↓**SLC6A12,** ↓**SNHG11 (includes EG:319317),**↑**SNX10,**SOX4, SPCS2, SSBP2, STAT3, ↓**TECR,** TGFB1, TLR4, ↑**TMEM184B,** ↑**TMSB4X,** TNF, ↑**TTC3,** TWIST2, TXN2, ↓**UPP2,** VCL, VIM, WISP1	Lipid Metabolism, Small Molecule Biochemistry, Cell Cycle	93	60
3	↓**ABCA6,** ↓**ACAT2,** ACTR3, AIP, ALDH16A1, AMD1, ANAPC10, APOA4, ARL4C, ATP, ↓**BHMT,**↓**BLOC1S1,**C10ORF10, ↓**C14ORF147,**↓**C8G,** CABC1, CCDC59, CD244, CDC23 (includes EG:8697),CDH13, CELSR1, ↑**CHAC1,**↓**CHI3L1,**↓**CLCN2,**CLTCL1, Coup-Tf, ↓**CPPED1,**CTSD, CYB5A, CYP27A1, Cytochrome p450, DAD1, ↑**DBNDD2,**↓**DCXR,**↑**DPP7,** EEF1D, enoyl-CoA hydratase, Eotaxin, ↓**FAHD2A,** ↓**FAM158A,** ↓**FAM73B,** FGB, FOS, GAD1, ↑**GARS,** GAS7, GBP1 (includes EG:2633),glutamine, GRIK2, ↓**HAAO,** HADHA, ↑**HADHB,** HNF4A, ↓**HPD,** HTT, hydrolase, IFITM1, IFNA2, IFNG, IL11RA, KDM5A, leukotriene C4, ↑**LMAN2L,**LOC100129193, MDH1, ↓**MFSD2A,** MIR181A1, MIR205 (includes EG:406988),MIRN324, ↑**MMP12,** MSR1, MT1L, ↑**NCEH1,**↑**NCKAP1L,**ND3, ND4L, ↓**NDUFA3,**NDUFA9 (includes EG:4704),NDUFS1, NDUFS3, NDUFS4, NDUFS5, ↓**NDUFS8,**NDUFS6 (includes EG:4726),NDUFV2, ↓**NIPSNAP1,**↓**NNMT,**NPTX1, NR3C1, ↓**OLIG1,**↓**OTC,** PARP9, PCSK1, PCSK6, PDE4B, PIK3CD, PLAGL1, ↓**PLSCR2,** PNPT1, PPARGC1B, ↑**PRCP,** PSMA5, ↓**RAET1B,** REXO2, ↑**RPL31,**↓**SAT2,** ↓**SDS,** SEC11A, SHH, ↑**SLC15A3,** ↓**SLC22A18,** ↓**SLC29A1,** ↓**SLC38A4,**↓**SLC44A1,**↓**SLCO2B1,**SP2, SPCS3, SRP54, SUPT7L, TADA1, TBC1D17, ↓**TCEA3,** TCF7, THRB, TICAM2, ↓**TIMD2,** TLR1, TMEM158,↑**TMEM208,** TNFAIP6, TNFRSF8, TNFSF9, TNFSF14, TRAF6, TTC35, TTRAP, ↑**UHRF2,** ULBP2, ↑**USP18,** ↑**WBP5**	Cell Morphology, Cellular Compromise, Genetic Disorder	71	49

Network-2

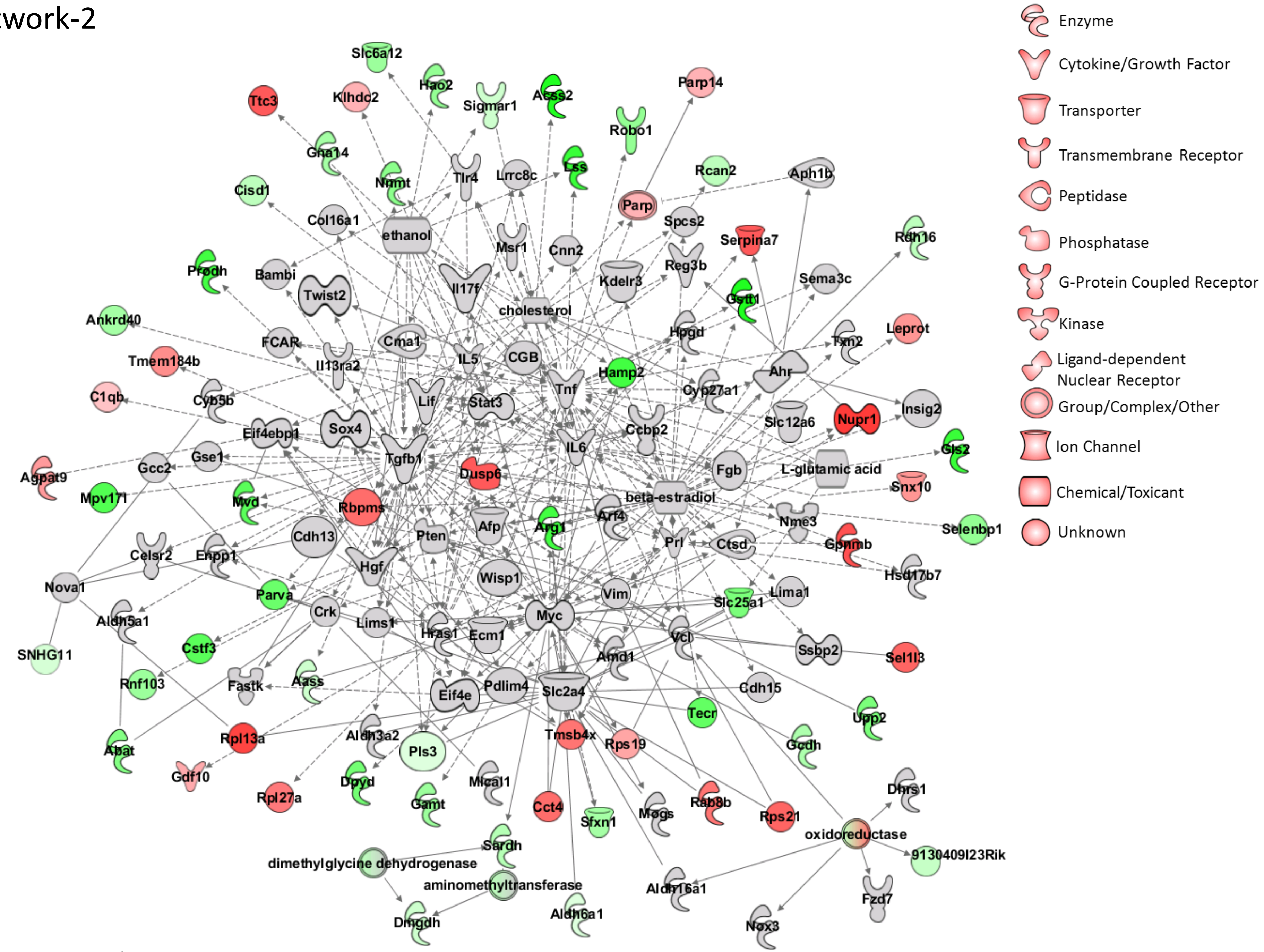

Sharma *et al* , Figure-S4A

Network-3

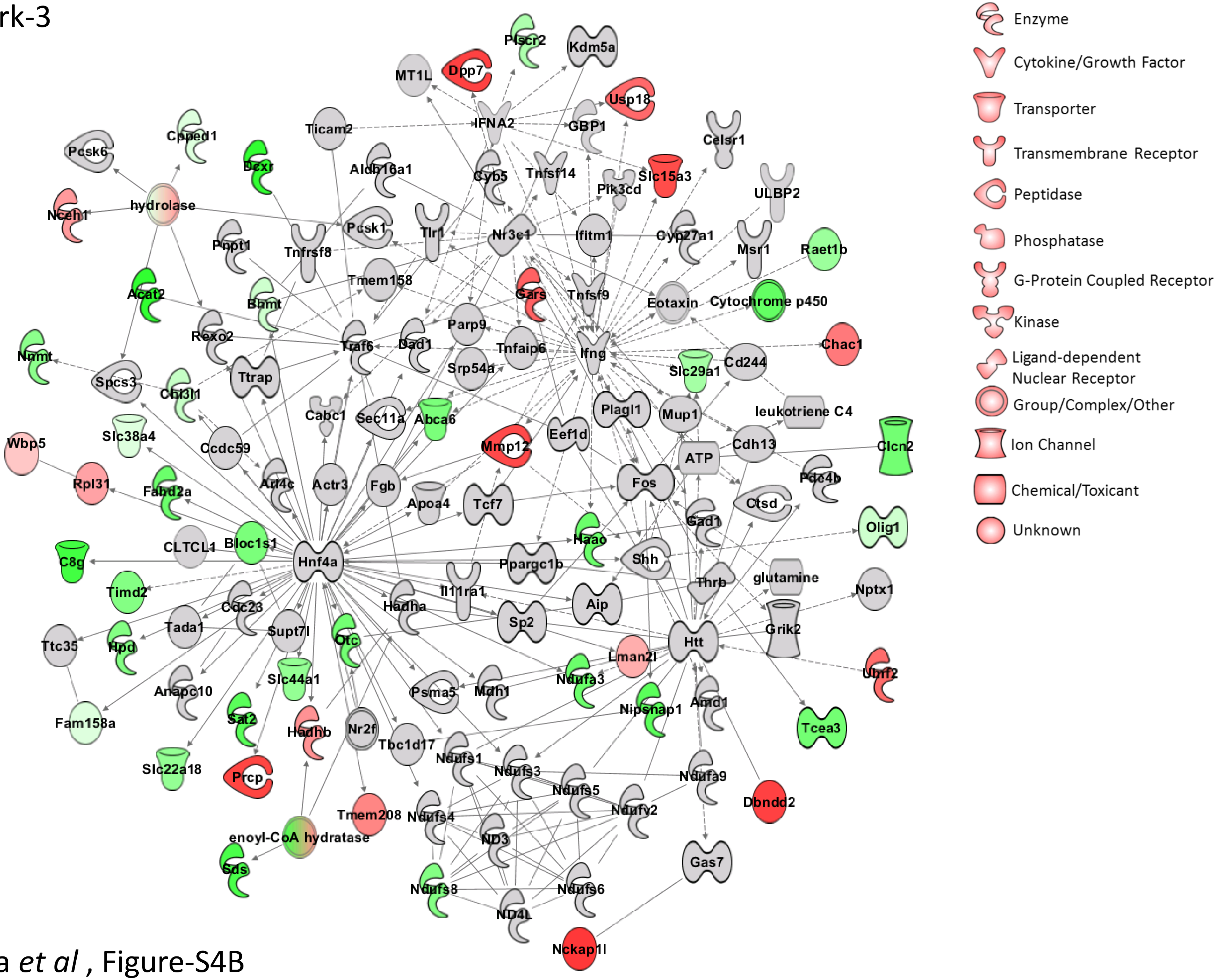

Sharma *et al* , Figure-S4B
