# Supplementary material for: Hepatic Gene Expression Profiling Reveals Key Pathways Involved in Leptin-Mediated Weight Loss in ob/ob Mice
Source: PLoS One. 2010 Aug 16;5(8):e12147. doi: 10.1371/journal.pone.0012147 (PMC2922341; doi:10.1371/journal.pone.0012147)
Supplement: Table S1 — Differentially expressed genes could be sub-divided into two major groups: 116 genes of Cluster-1 and Cluster-2 (group-A), and 98 genes of Cluster-3 and Cluster-4 (group-B). These two groups of genes were separately analyzed and were mapped to cellular compartments and biological functions. (0.08 MB DOC) [file pone.0012147.s005.doc]

**Table S1: Comparison of 116 genes of Cluster-1 & Cluster-2, with 98 genes of Cluster-3 & Cluster-4.**

| **Cellular Compartments:** | | | |
| --- | --- | --- | --- |
| **Group-A (Cluster-1 and 2)** | **Group-B (Cluster-3 and 4)** | | |
| **Mitochondrion ( 19 ):**  Ndufs8, Gck, Slc25a1, Otc, Sardh, Cisd1, Sfxn1, Kynu, Gcdh, Aldh6a1, Dbi, Dmgdh, Hsd17b10, Aass, Nipsnap1, Abat, Cyba, Slc25a33, Hsp90ab1 | **Mitochondrion ( 14 ):**  Ndufa3, Macrod1, Ass1, Mmab, Uqcrc1, Cyb5r3, Gls2, Cyp17a1, Prodh, Hsd3b2, Haao Hadhb, Ung, Mrps6 | | |
| **Cytosol ( 10 ):**  Rps11, Pla2g4f, Kynu, Gck, Tpmt, Rpl27a, Rps19, Dpyd, Tmsb4x, Ahcy | **Cytosol ( 10 ):**  Selenbp2, Mvd, Bloc1s1, Cyb5r3, Psen2, Zfp36, Fn3k, Cct4, Dpp7, Ctnnb1 | | |
| **Endoplasmic reticulum ( 11 ):**  Faah, 1110002B05Rik, Abca6, Es22, Hsd17b10, Oprs1, Rdh16, Aadacl1, Hsp90b1, Lman2l, Srd5a3 | **Endoplasmic reticulum ( 11 ):**  Cyp2c29, Scd1, Hpd, Gpsn2, Cyb5r3, Psen2, Cyp17a1, Pcsk9, Hsd3b2, Agpat9, Pla2g12a | | |
| **Plasma membrane (**Not enriched) | **Plasma membrane ( 6 )**  Psen2, C8g, Slc29a1, Slc6a12, Aqp1, Gpnmb | | |
| **Vacuole /lysosome (**Not enriched) | **Vacuole /lysosome ( 5 )**  Rilp, Ttc3, Dpp7, Slc15a3, Prcp | | |
| **Biological processes enriched in both groups:** | | | |
| **Biological Process** | | **Group-A (# genes)** | **Group-B (# genes)** |
| Amine metabolic process | | 13 | 8 |
| Carboxylic acid biosynthetic process | | 5 | 5 |
| Carboxylic acid catabolic process | | 9 | 4 |
| Carboxylic acid metabolic process | | 19 | 12 |
| Cellular amine metabolic process | | 10 | 7 |
| Cellular amino acid &derivative metabolic process | | 14 | 10 |
| Cellular amino acid metabolic process | | 10 | 7 |
| Cellular ketone metabolic process | | 19 | 12 |
| Cellular metabolic process | | 60 | 49 |
| Cellular nitrogen compound metabolic process | | 15 | 7 |
| Cofactor biosynthetic process | | 4 | 4 |
| Cofactor metabolic process | | 8 | 7 |
| Lipid metabolic process | | 12 | 14 |
| Metabolic process | | 65 | 59 |
| Organic acid biosynthetic process | | 5 | 5 |
| Organic acid catabolic process | | 9 | 4 |
| Organic acid metabolic process | | 19 | 12 |
| Oxidation reduction | | 15 | 13 |
| Oxoacid metabolic process | | 19 | 12 |
| Response to chemical stimulus | | 17 | 18 |
| **Biological processes enriched only in group A (Cluster-1 & Cluster-2)** | | | |
| **Biological Process** | | | **# genes** |
| Amine catabolic process | | | 7 |
| Catabolic process | | | 22 |
| Cellular amino acid catabolic process | | | 7 |
| Cellular aromatic compound metabolic process | | | 10 |
| Cellular catabolic process | | | 17 |
| Fatty acid metabolic process | | | 6 |
| Heterocycle catabolic process | | | 4 |
| Homeostasis of number of cells | | | 4 |
| Innate immune response | | | 5 |
| Lymphocyte mediated immunity | | | 4 |
| Monocarboxylic acid metabolic process | | | 9 |
| Nitrogen compound biosynthetic process | | | 7 |
| Nitrogen compound catabolic process | | | 8 |
| Nucleobase, nucleoside and nucleotide metabolic process | | | 7 |
| Peptide metabolic process | | | 4 |
| Response to extracellular stimulus | | | 6 |
| Response to metal ion | | | 4 |
| Response to nutrient levels | | | 5 |
| Sulfur metabolic process | | | 5 |
| **Biological processes enriched only in group B (Cluster-3 & Cluster-4)** | | | |
| **Biological Process** | | | **# genes** |
| Cellular lipid metabolic process | | | 13 |
| Cholesterol metabolic process | | | 4 |
| Coenzyme metabolic process | | | 6 |
| Glutamine family amino acid metabolic process | | | 4 |
| Inflammatory response | | | 6 |
| Lipid biosynthetic process | | | 9 |
| Myeloid cell differentiation | | | 4 |
| Steroid biosynthetic process | | | 6 |
| Steroid metabolic process | | | 7 |
| Sterol metabolic process | | | 4 |
